# Crosstalk in the brain tumor microenvironment: mechanisms, therapeutic strategies, and clinical advances

**DOI:** 10.1016/j.mmr.2026.100047

**Published:** 2026-06-20

**Authors:** Gui-Long Tanzhu, Liu Chen, Jiao-Yang Ning, Gang Xiao, Su Wen, Ce Wang, Hao Wen, Ke-Qin Zhang, Jie Yang, Mu-Yuan Jia, Hai-Bin Deng, Thomas Michael Marti, Paul R Lockman, Rong-Rong Zhou

**Affiliations:** a Department of Oncology, Xiangya Hospital, Central South University, Changsha 410000, China; b Department of Radiation Oncology, Hunan Cancer Hospital/Affiliated Hospital of Xiangya School of Medicine, Central South University, Changsha 410000, China; c Key Laboratory of Translational Radiation Oncology, Department of Radiation Oncology, Hunan Cancer Hospital, Changsha 410000, China; d Department of Radiology, China-Japan Friendship Hospital, Beijing 100029, China; e Department of Nuclear Medicine, the Third Xiangya Hospital, Central South University, Changsha 410000, China; f Department of Dermatology, Xiangya Hospital, Central South University, Changsha 410000, China; g Department of Neurosurgery, Xiangya Hospital, Central South University, Changsha 410000, China; h National Clinical Research Center for Geriatric Disorders, Xiangya Hospital, Central South University, Changsha 410000, China; i Department of General Thoracic Surgery, Inselspital, Bern University Hospital, University of Bern, Bern 3008, Switzerland; j Department for BioMedical Research, University of Bern, Bern 3008, Switzerland; k Department of Neuroscience, West Virginia University School of Medicine, Rockefeller Neuroscience Institute, Morgantown, WV 26505, USA; l Department of Pharmaceutical Sciences, West Virginia University School of Pharmacy, Morgantown, WV 26505, USA; m Xiangya Lung Cancer Center, Xiangya Hospital, Central South University, Changsha 410000, China

**Keywords:** Brain tumor, Tumor microenvironment, Immunotherapy, Targeted therapy, Single-cell sequencing, Spatial transcriptomics

## Abstract

Brain tumors, including primary intracranial tumors and brain metastases (BrMs), represent a major threat to human health and are associated with extremely poor prognoses. The brain tumor microenvironment (BTME) is a complex ecosystem composed of various elements, including tumor cells, immune cells, neurons, the vascular system, the extracellular matrix, and cytokines. These elements not only coexist spatially but are also functionally connected, interacting through intricate networks that collectively influence tumor initiation, progression, and therapeutic efficacy. In recent years, the application of novel technologies such as single-cell and spatial transcriptomics has uncovered complex cellular heterogeneity and spatial organization within the BTME. This review comprehensively summarizes the key components and functions of the BTME in tumor development and therapy, the mechanisms underlying intercellular interactions, with an emphasis on their clinical potential and challenges.

## Background

Brain tumors, including brain metastases (BrMs) and primary intracranial tumors, pose serious threats to human health and are associated with poor prognoses [1], [2], [3]. Epidemiological data indicate that the annual incidence of primary intracranial tumors is 6–10 cases per 100,000 people [4], [5], whereas the annual incidence of BrMs is even higher, at 8–10 cases per 100,000 people [6]. Gliomas are the most prevalent type of primary intracranial tumors, accounting for 20%–30% of all brain tumor cases. In particular, malignant gliomas, such as glioblastoma multiforme (GBM), have exceptionally poor prognoses, with a 5-year survival rate of less than 10% [7], [8]. BrMs most commonly originate from solid tumors such as lung cancer, breast cancer, and melanoma. The incidence of BrMs continues to increase alongside the rising prevalence of these primary tumors [9], [10]. Brain tumors pose significant global public health challenges due to their high incidence and poor survival outcomes [11], [12]. Although conventional treatments such as surgery, radiotherapy, and chemotherapy have improved survival to some extent, their overall efficacy remains limited.

The understanding of the brain’s immune microenvironment and lymphatic drainage system has undergone a transformative evolution. Early studies by Shirai [13] and Medawar [14] in the early to mid-20th century established the concept of “brain immune privilege”, which proposed that the blood-brain barrier (BBB) isolates the central nervous system (CNS) from the peripheral immune system [15], [16]. Since the 1980s, this view has been progressively revised, with the identification of microglia as the resident immune cells that monitor the brain microenvironment and play critical roles in neurodegenerative diseases [17]. In 2012, Iliff *et al*. [18] introduced the concept of the glymphatic system, a brain clearance pathway analogous to the lymphatic system. Subsequent work by Rasmussen *et al*. [19] revealed that astrocytes mediate cerebrospinal fluid-interstitial fluid exchange via aquaporin-4 (AQP4) water channels, providing a mechanistic basis for metabolic waste clearance in the brain. In 2015, a landmark study by Louveau *et al.* [20] provided evidence of functional lymphatic vessels in the meninges, establishing a direct anatomical connection between the brain and the peripheral immune system and overturning the long-held belief that the brain lacks lymphatic drainage. In recent years, the advances of *in vivo* imaging, single-cell sequencing, and other advanced technologies have elucidated the roles of meningeal lymphatic vessels and the glymphatic system in various neurological diseases, such as Alzheimer’s disease, multiple sclerosis, stroke, and brain tumor [21], [22], [23], [24]. These discoveries have reshaped our understanding of neuroimmune interactions and laid a theoretical foundation for developing novel therapies [25].

Traditional cancer research has focused primarily on tumor cells themselves. However, accumulating evidence indicates that the tumor microenvironment (TME) plays a critical role in tumor initiation, progression, and therapeutic resistance [26], [27], [28]. In GBM, tumor evolution is driven mainly by microenvironmental reprogramming rather than by intrinsic molecular evolution of tumor cells [29]. The brain TME (BTME) is a highly heterogeneous and dynamically evolving ecosystem composed of tumor cells, immune cells, neurons, the vascular system, the extracellular matrix (ECM), and cytokines [30]. These components interact through complex signaling networks that collectively shape tumor biology [31]. For example, tumor-associated macrophages (TAMs) promote tumor cell proliferation by secreting growth factors while concurrently suppressing anti-tumor immune responses [32], [33], [34]. Abnormal tumor vasculature not only supplies nutrients for tumor growth but also impedes effective drug delivery and immune cell infiltration [35], [36]. Moreover, ECM remodeling influences the migratory and invasive properties of tumor cells [37]. Therefore, a comprehensive exploration of the BTME is essential for elucidating the molecular mechanisms of tumor development and identifying new therapeutic targets [38]. Moreover, therapies targeting the BTME, including immunotherapy and targeted therapy, have shown promising results in clinical trials and are emerging as innovative treatment strategies [39], [40].

In recent years, multi-omics technologies have further revealed the dynamic evolution and spatial specificity of cellular states within the BTME [9], [41], [42]. For instance, in GBM, tumor cells and myeloid cells have been shown to form stable interaction networks mediated by specific ligand-receptor pairs, such as secreted phosphoprotein 1 (SPP1)-CD44, which promote invasive tumor phenotypes and contribute to the establishment of an immunosuppressive microenvironment [43], [44]. Furthermore, exhaustion of regulatory T cells (Tregs) has been reported to enhance myeloid cell activation and improve the responsiveness of GBM to programmed cell death protein 1 (PD-1) blockade and tumor-targeting antibodies [45]. Metabolic alterations within the BTME, including the accumulation of lactate and kynurenine, can also directly modulate the functions of T cells and macrophages, constituting a novel form of “metabolic checkpoint” [46]. These findings highlight the therapeutic potential of targeting cellular interactions and metabolic pathways within the BTME.

**Fig. 1:**
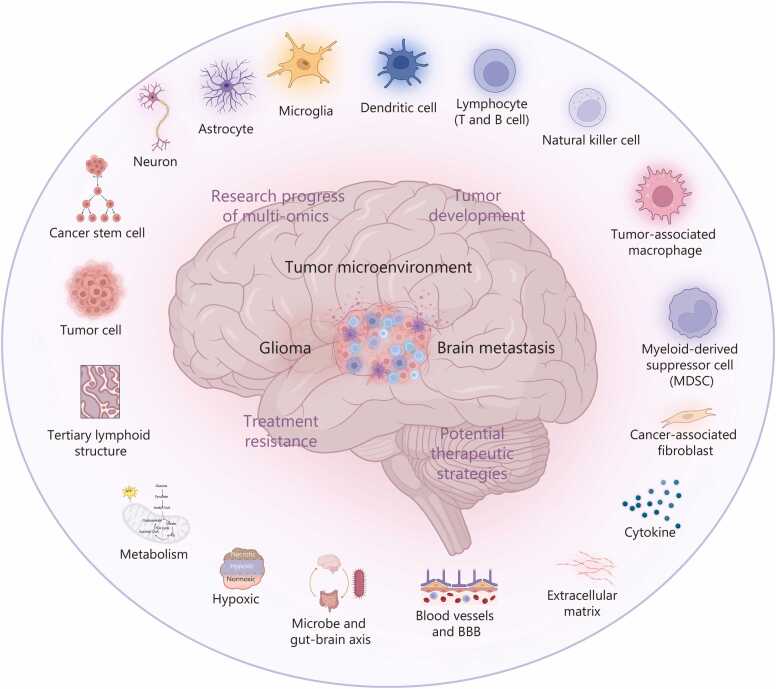
**Crosstalk among various components of BTME.** The manuscript primarily focuses on various cellular and non-cellular components of BTME, with particular emphasis on the modules “research progress of multi-omics”, “tumor development”, “treatment resistance” and “potential therapeutic strategies”. “Research progress in multi-omics” refers to advancements in transcriptomics, including bulk RNA-seq, single-cell RNA-seq, spatial RNA-seq, and novel imaging techniques. “Tumor development” primarily investigates the roles and mechanisms of the interplay between the tumor and BTME during tumorigenesis and progression. “Treatment resistance” mainly refers to how the tumor microenvironment interaction leads to chemotherapy resistance, immunotherapy resistance, and other forms of resistance. “Potential therapeutic strategies” explore whether targeting BTME can inhibit tumor progression and reverse treatment resistance. BTME. Brain tumor microenvironment; BBB. Blood-brain barrier.

This review summarizes recent advances in BTME research, explores its potential implications for cancer therapy, and discusses future directions. We systematically delineate the core cellular and molecular constituents of the BTME, including tumor cells, immune populations, neurons, the vascular system, the ECM, and cytokine networks. Subsequently, we integrate dynamic and reciprocal interactions that govern tumor evolution, with particular emphasis on the context-dependent and bidirectional functions of these components in orchestrating tumor progression, immune evasion, and therapeutic resistance. We further highlight emerging paradigms, such as the gut-brain axis and tertiary lymphoid structures (TLSs), alongside recent advances in multimodal and spatially resolved technologies that are reshaping our understanding of BTME complexity. In parallel, we synthesize current BTME-targeted therapeutic strategies, including targeted therapy, immunotherapy, and combination approaches. Finally, we discuss key challenges and future directions for translating BTME-focused insights into clinical practice **(**Fig. 1**)**.

## Composition and dynamics of the BTME

The BTME is composed of various cells, the vascular system, and ECM components. Under physiological conditions, these components maintain brain homeostasis. However, during tumorigenesis, they interact with tumor cells to influence tumor progression and therapeutic resistance. Here, we discuss the roles and dynamic changes in BTME, including tumor cells, cancer stem cells (CSCs), neurons, glial cells, immune cells, cancer-associated fibroblasts (CAFs), the vascular system, ECM, and cytokines in brain tumor [47]. The roles of TLSs and the gut-brain axis are also described.

### Tumor cells

**Fig. 2:**
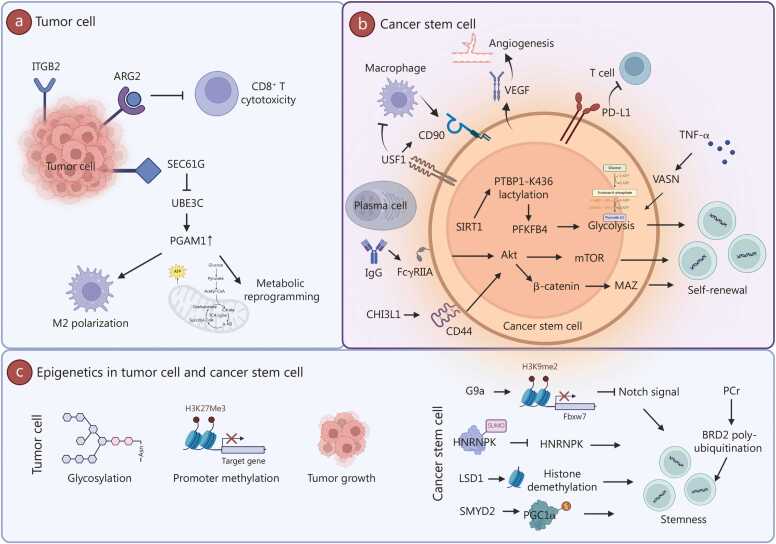
**Multilayered regulation of brain tumor progression: gene expression, stemness maintenance, and epigenetic remodelling. a** Gene regulation in tumor cells. ITGB2 regulates glioma cell proliferation and migration. Elevated ARG2 expression is associated with reduced CD8^+^ T cell cytotoxicity and decreased T cell infiltration in breast cancer BrM. SEC61G promotes BrM by antagonizing UBE3C, preventing the proteasomal degradation of PGAM1. Stabilized PGAM1 promotes glycolysis and modulates oxidative phosphorylation, driving metabolic reprogramming that facilitates brain colonization. SEC61G also reshapes TIME by promoting M2 polarization. **b** Maintenance of tumor stemness and microenvironmental interactions. VASN mediates TNF-α-induced GSC self-renewal by promoting glycolysis. SIRT1 induces K436 lactylation of PTBP1, reducing its ubiquitination and stabilizing *PFKFB4* mRNA, further increasing glycolytic activity and supporting GSC maintenance. PCr promotes GSC growth by inhibiting BRD2 polyubiquitination. Plasma cell-derived IgG stimulates GSC proliferation via FcγRIIA-protein kinase B (Akt)- mTOR signalling axis. CHI3L1 activates CD44-Akt-β-catenin signalling, induces MAZ-dependent CD44 expression and forms a pro-mesenchymal positive feedback loop. USF1 promotes GSC stemness through CD90 upregulation, facilitating TAMs adhesion and immunosuppressive polarization. GSCs secrete VEGF to promote angiogenesis and suppress T cell function via PD‑L1 expression. **c** Epigenetic regulation in tumor cells and cancer stem cells. Loss of H3K27me3 enhances MMP3 expression and drives breast cancer BrM. Epigenetic regulation plays a critical role in GSC heterogeneity. G9a suppresses Fbxw7 through H3K9me2 to modulate Notch signalling. SUMOylation alters HNRNPK transcriptional activity. LSD1-mediated histone demethylation activates metastasis-associated genes. SMYD2-induced methylation of PGC1α contributes to GSC maintenance. ARG2. Arginase 2; BrM. Brain metastases; SEC61G. SEC61 translocon subunit gamma; UBE3C. Ubiquitin protein ligase E3C; PGAM1. Phosphoglycerate mutase 1; TIME. Tumor immune microenvironment; VASN. Vasorin; TNF. Tumor necrosis factor; GSC. Glioma stem cell; SIRT1. Sirtuin 1; PTBP1. Polypyrimidine tract-binding protein 1; PFKFB4. 6-phosphofructo-2-kinase/fructose-2,6-biphosphatase 4; PCr. Phosphocreatine; BRD2. Bromodomain-containing protein 2; IgG. Immunoglobulin G; FcγRIIA. Fc gamma receptor IIA; mTOR. Mammalian target of rapamycin; CHI3L1. Chitinase 3-like 1; MAZ. MYC-associated zinc finger protein; USF1. Upstream transcription factor 1; TAMs. Tumor-associated macrophages; VEGF. Vascular endothelial growth factor; PD-L1. Programmed death-ligand 1; H3K27me3. Histone H3 lysine 27 trimethylation; MMP3. Matrix metalloproteinase 3; Fbxw7. F-box and WD repeat domain-containing 7; H3K9me2. H3 lysine 9 dimethylation; HNRNPK. Heterogeneous nuclear ribonucleoprotein K; LSD1. Lysine-specific demethylase 1; SMYD2. SET and MYND domain-containing protein 2; PGC1α. Peroxisome proliferator-activated receptor gamma coactivator 1-alpha; ITGB2. Integrin subunit β 2.

Tumor heterogeneity exists both among individuals and within a single tumor, posing major challenges for diagnosis and treatment. Intratumoral heterogeneity is a defining feature of brain tumors and is particularly prominent in GBM [48]. Single-cell sequencing has revealed multiple subpopulations of tumor cells, characterized by distinct transcriptional profiles and functional properties [49]. Highly heterogeneous GBM tumor cells can also be classified into mesenchymal-like, astrocyte-like (AC-like), oligodendrocyte precursor cell-like (OPC-like), and neural progenitor cell-like (NPC-like) subtypes [43], [49]. Several key genes, including arginase 2 (*ARG2*), SEC61 translocon subunit gamma (*SEC61G)*, and integrin subunit beta 2 (*ITGB2)*, have been identified as critical regulators of tumor progression. Upregulation of ARG2 is associated with the functional suppression of T cells [50]. SEC61G promotes glycolysis and modulates oxidative phosphorylation by stabilizing key glycolytic enzyme phosphoglycerate mutase 1 (PGAM1) through ubiquitin protein ligase E3C (UBE3C) inhibition [51]. Moreover, SEC61G contributes to BTME immunosuppression by promoting M2 polarization of microglia, increasing interleukin (IL)‑6 and IL-10 secretion, and inhibiting TLSs maturation [51]. ITGB2 regulates glioma cell proliferation and migration by modulating macrophage recruitment and polarization, further highlighting the bidirectional interactions between tumor cells and the BTME [52], [53] **(**Fig. 2**a)**.

Epigenetics also plays a critical role in brain tumor development. Westphal *et al*. [54] identified 38 epigenetic regulators as unique epigenetic markers of intracranial melanoma. In BrMs from breast cancer, aberrant protein glycosylation has been implicated, with a significant increase in highly diallylated N-glycans such as HexNAc_5_Hex_6_DeoxyHex_1_NeuAc_3_ sialylated [55]. Mechanistically, loss of the histone methyltransferases lysine methyltransferase 2 C (*KMT2C*) and lysine methyltransferase 2D (*KMT2D*) promotes KDM6A-mediated demethylation of *H3K27me3*, leading to the upregulation of matrix metalloproteinase-3 (MMP3) and facilitating brain-specific metastasis of breast cancer [53]. In summary, these findings reveal a regulatory network underlying the epigenetic and molecular heterogeneity of brain tumors, offering new insights into their pathogenesis **(**Fig. 2**c)**.

### Cancer stem cells

Cancer stem cells (CSCs) represent a critical subpopulation of tumor cells with stem cell-like properties, including self-renewal and multipotent differentiation capacity, enabling them to drive tumorigenesis, recurrence, and therapeutic resistance [56], [57], [58]. Glioma stem cells (GSCs) are considered critical contributors to tumor progression [56]. GSCs are commonly categorized into four subtypes, including proneural, neural, classical, and mesenchymal GSCs [59], [60]. Specific GSC targets include mesenchyme homeobox 2 (MEOX2) in classical GSCs and serglycin (SRGN) in mesenchymal GSCs. The MEOX2-NOTCH and SRGN-nuclear factor κB (NF-κB) pathways are essential for maintaining the stemness of classical and mesenchymal GSCs, respectively [61]. CSC subtype switching is a complex process influenced by both intrinsic tumor programs and extrinsic microenvironmental cues. Glycoprotein NMB (GPNMB), highly expressed in macrophages, promotes proneural-mesenchymal transition (PMT) and impedes T cell activation in GBM [62]. Macrophage-derived oncostatin M (OSM) interacts with glycoprotein 130 (GP130) on GBM cells, which activates signal transducer and activator of transcription 3 (STAT3) signaling and induces a transition of GBM cells into mesenchymal-like states [63]. Mesenchymal GSCs exhibit enhanced malignancy and radioresistance, which are closely associated with the immunosuppressive BTME [59] **(**Fig. 2**b)**.

The maintenance of GSC stemness is governed by a multilayered regulatory network involving hypoxic microenvironment [64], epigenetic modifications such as SET and MYND domain‑containing protein 2 (SMYD2)-mediated the peroxisome proliferator-activated receptor gamma 1α (PGC1α) methylation [65], metabolic reprogramming including vasorin (VASN)-enhanced glycolysis [66], and BTME modulation via nonclassical NF-κB (RELB/p50) activation [67]. In GSC, phosphocreatine (PCr) inhibits the bromodomain‑containing protein 2 (BRD2) polyubiquitination through interaction with the E3 ubiquitin ligase speckle type BTB/POZ protein (SPOP), ultimately promoting tumor growth [68]. Sirtuin 1 (SIRT1)-induced lactylation of the polypyrimidine tract-binding protein 1 (PTBP1) stabilizes 6-phosphofructo-2-kinase/fructose-2,6-bisphosphatase 4 (*PFKFB4*) mRNA, enhancing glycolytic flux and highlighting the interplay between metabolic and epigenetic regulation in maintaining GSC stemness [69].

The interaction between GSCs and the BTME forms a complex regulatory network to sustain tumor stemness and progression. GSCs maintain autonomous stemness by secreting factors such as IgG and chitinase 3-like 1 (CHI3L1) and activating signaling pathways including Fc gamma receptor IIA (FcγRIIA)-protein kinase B (Akt)-mammalian target of rapamycin (mTOR) [70] and Akt/β-catenin [71]. Concurrently, BTME components, including pericytes and TAMs, support GSCs via cytokine secretion and intercellular interactions [71], [72]. Notably, the upstream stimulating factor 1 (USF1)/CD90 signaling axis directly regulates GSC stemness and mediates crosstalk with TAMs, forming a positive feedback loop that promotes tumor proliferation [72]. In addition, GSCs secrete vascular endothelial growth factor (VEGF) to promote angiogenesis and simultaneously express programmed death‑ligand 1 (PD-L1) to suppress T cell activity [73]. In BrMs, CD44^+^ CSCs activate Wnt7-β-catenin signaling pathway via the G protein‑coupled receptor 124 (GPR124) receptor, promoting transendothelial migration and lung cancer BrM [74].

Epigenetic regulation is a key determinant of GSC heterogeneity. Histone methyltransferase 2 (G9a) suppresses F-box and WD repeat domain containing 7 (Fbxw7) transcription by H3K9me2 modification, which subsequently influences the Notch signaling pathway and decreases stemness in GSCs [75]. The self-renewal of GSCs is sustained by DARS1-AS1/YBX1-controlled posttranscriptional circuits [76]. SUMOylation of the K422 residue of HNRNPK interferes with its DNA binding ability, reducing transcription and promoting transition between GSCs [77]. Lysine-specific demethylase 1 (LSD1) drives H3K9me2 demethylation, promotes RNF135 transcription, and enhances tumor stemness [78]. These findings provide new perspectives for understanding the molecular characteristics of GSCs **(**Fig. 2**c)**.

Key biomarkers of tumors and CSCs, as well as phenotypic transformation between GSCs, may provide therapeutic strategies [61], [79], [80]. Furthermore, innovative dual stem cell therapy approaches that integrate oncolytic viruses and gene editing technologies have shown promise. For example, a strategy combining a stem cell line carrying an oncolytic herpes simplex virus (oHSV) with a CRISPR-Cas9-engineered stem cell line lacking the nectin-1 receptor has demonstrated enhanced anti-tumor efficacy through improved viral sensitivity and secretion of immunoregulatory factors such as granulocyte-macrophage colony-stimulating factor (GM-CSF) [81]. Overall, recent in-depth research into tumor cells or CSCs themselves or their interactions with TME has led to the discovery of different targets, encouraging researchers to translate these findings into clinical applications [82], [83], [84].

### Neurons

**Fig. 3:**
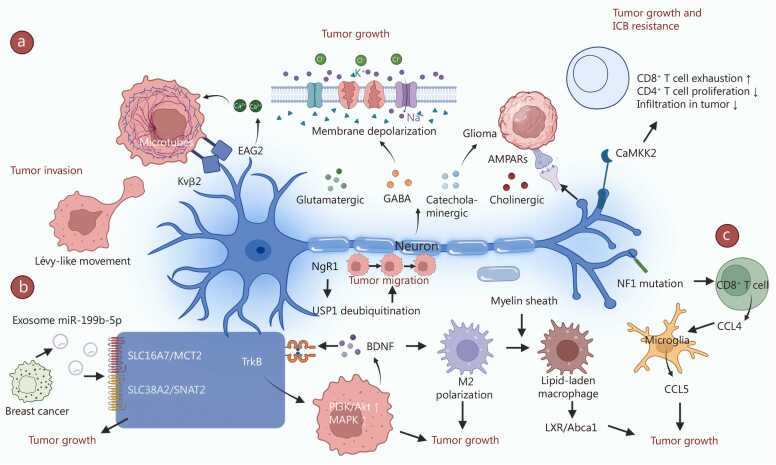
**Neuron-tumor interactions and their regulatory role in the BTME.** In a brain tumor, tumor cells establish extensive functional interactions with neurons that promote invasion, growth, and immune suppression. **a** Ion channels such as EAG2 and Kvβ2 are enriched at neuron-tumor contact sites in GBM, where they enhance calcium signalling. Glioma cells form functional synaptic connections with neurons, including glutamatergic, GABAergic, and catecholaminergic synapses, and migrate through the brain using neuron-like Lévy-type movement patterns. Neuronal activity induces membrane depolarization and Ca^2+^ influx in tumor cells, accelerating tumor growth and invasion. **b** In breast cancer BrM, tumor-derived extracellular vesicles are enriched in miR-199b-5p target SLC38A2, SLC16A7 in neurons, and SLC1A2/EAAT2 astrocytes, disrupting neuron-astrocyte metabolic coupling and favouring tumor growth. **c** Neuron-tumor signalling also contributes to immune suppression. Activation of the CaMKK2 pathway promotes CD8^+^ T cell exhaustion, limits CD4^+^ T cell expansion, and maintains immunosuppressive microglia-like myeloid states, facilitating resistance to immune checkpoint blockade. Tumor cells release neurotransmitters, and neurotrophic factors such as glutamate and BDNF promote M2 macrophage polarization and activate neuronal TrkB receptors, stimulating PI3K/Akt and MAPK signalling to support tumor proliferation. Myelin-derived lipids phagocytosed by tumor-associated macrophages generate lipid-laden macrophages that transfer lipids to tumor cells via the LXR/Abca1 pathway, supporting tumor growth. In *NF1*-mutant neurons, microglia-derived CCL5 ultimately promotes GSCs’ survival. BTME. Brain tumor microenvironment; EAG2. Ether-a-go-go 2; Kvβ2. Potassium voltage-gated channel subfamily beta member 2; GBM. Glioblastoma; GABA. Gamma-aminobutyric acid; NKCC1. Sodium-potassium-chloride cotransporter 1; CaMKK2. Calcium/calmodulin-dependent protein kinase kinase 2; BrM. Brain metastases; SLC. Solute carrier; EAAT2. Excitatory amino acid transporter 2; BDNF. Brain-derived neurotrophic factor; TrkB. Tropomyosin receptor kinase B; PI3K. Phosphoinositide 3-kinase; Akt. Protein kinase B; MAPK. Mitogen-activated protein kinase; LXR. Liver X receptor; Abca1. ATP-binding cassette transporter A1; NF1. Neurofibromatosis 1; CCL. C-C motif chemokine ligand; GSCs. Glioma stem cells; AMPARs. α‑amino‑3‑hydroxy‑5‑methyl‑4‑isoxazolepropionic acid receptors; USP1. Ubiquitin specific peptidase 1.

Neurons serve as the fundamental structural and functional units of the nervous system, responsible for information reception, integration, and transmission. The neuron-tumor crosstalk plays an important role in tumor progression [85], [86], [87]. Neurons participate in the cancer-associated stress responses and tumor progression [88], [89], in part through corticotropin-releasing hormone secretion, prompting intensive investigation of neuron-tumor interactions [90], [91]. Isocitrate dehydrogenase (IDH) wild-type glioma exhibits increased aggressiveness upon recurrence, accompanied by increased expression of neuronal signaling factors, indicating neuronal interactions facilitate tumor progression [92]. The single-cell transcriptomic analysis revealed a high abundance of malignant stem cell-like populations of neuronal lineage in GBM, including neural progenitor-like, astrocyte-like, oligodendrocyte progenitor-like, oligodendrocytes, or excitatory neurons [93] **(**Fig. 3**)**.

Neuronal activity profoundly influences tumor behaviour through synaptic transmission and electrical signaling. Ion channels such as EAG2 and Kvβ2 localize to neuron-glioma contact sites and promote tumor invasion [94]. Neuregulin-1 (NgR1) modulates glioma migration on myelin via ubiquitin-specific peptidase 1 (USP1)-mediated stabilization of inhibitor of DNA binding 1 (ID1) [95]. Glioma cells also exhibit neuron-like migratory characteristics and accelerate invasion in response to neuronal activity-induced calcium signaling [96]. Oncogenic variants such as phosphatidylinositol-4,5-bisphosphate 3-kinase catalytic subunit alpha (PIK3CA) induce brain hyperexcitability and synaptic remodeling [97], while semaphorin 4 F (SEMA4F) and chromodomain helicase DNA binding protein 2 (CHD2)-FOS-like antigen 1 (FOSL1) signaling promotes tumor progression by remodeling synaptic communication in GBM and diffuse midline glioma (DMG) [85], [98]. Additionally, neuronal activity facilitates tumor progression and treatment resistance by inducing PMT in GSCs. Neuronal activation increases the level of miR-184-3p in exosomes, which are subsequently absorbed by GSCs. This process inhibits RNA-binding motif protein 15 (RBM15) and decreases the m^6^A levels of discs large homolog 3 (DLG3), thereby inducing the PMT of GSCs through STAT3 pathway activation [99]. Furthermore, the TME can reprogram neural stem cells into CSCs, resulting in therapeutic resistance [100].

Glioma cells can form functional synapses with neurons, including glutamatergic synapses mediated by α‑amino‑3‑hydroxy‑5‑methyl‑4‑isoxazolepropionic acid receptors (AMPARs) and GABAergic synapses mediated by gamma‑aminobutyric acid (GABA) receptors. [101]. These cells express glutamate receptors and postsynaptic genes, enabling synaptic integration and activity-dependent proliferation [102], [103], [104]. Venkatesh *et al*. [105] found that AMPARs-mediated synaptic structures confer postsynaptic functionality to gliomas. Such structures, termed neurogliomal synapses (NGSs), allow direct neuronal control of tumor growth [106].

Beyond physical synapses, neurons also communicate with tumor cells through diverse neurotransmitters and receptors, regulating tumor growth, invasion, and microenvironmental adaptation. Neuron-derived glutamate is transmitted via NGSs, inducing membrane depolarization and Ca^2+^ influx, which further activate phosphoinositide 3-kinase (PI3K)/Akt and mTOR pathways to promote tumor progression, neuronal excitotoxicity, and epileptiform activity [107], [108], [109], [110], [111]. Beyond physical synapses, diverse neurotransmitter systems provide additional stimulatory cues. Local glutamatergic signaling and long-range cholinergic projections provide primary stimulatory cues. Specifically, acetylcholine promotes tumor progression via the cholinergic receptor muscarinic 3 (CHRM3) receptor, synergizing with the glutamate-driven calcium signaling [112]. This pro-tumorigenic effect extends to the catecholaminergic system, where neurons in the ventrolateral medulla have been shown to drive tumor growth [113]. In contrast, the inhibitory GABAergic signaling exhibits complex bidirectional effects. GABA_A_ receptor activation typically induces Cl⁻ influx and membrane hyperpolarization, inhibiting tumor proliferation. However, GABAergic input has a depolarizing effect on DMG cells due to Na⁺‑K⁺‑2Cl⁻ cotransporter 1 (NKCC1) chloride transporter function and consequently elevated intracellular chloride concentration, which promotes DMG growth [114]. Furthermore, the GABA_B_ receptor signaling also demonstrates dual roles. Its autocrine pathway can inhibit glycogen synthase kinase‑3 beta (GSK3β) and activate β-catenin, promoting immune escape and tumor growth, while simultaneously regulating neuronal firing frequency via G protein-coupled K^+^ channels to alleviate hyperexcitability [115], [116], [117], [118]. In BrMs, neuron-derived glutamate activates N‑methyl‑D‑aspartate receptors (NMDARs) and forms pseudo-tripartite synapses [119].

Conversely, glioma cells reciprocally remodel neural circuits and impair cognitive function and its prognosis [91], [101], [120]. Tumor cells further regulate neuronal function by releasing extracellular vesicles (EVs), neurotransmitters, and neurotrophic factors. Specifically, GBM-derived glutamate can overactivate neurons, leading to excitotoxic neuronal death and consequently creating space for tumor expansion [121]. Moreover, tumors secrete brain-derived neurotrophic factor (BDNF) to activate the TrkB receptor on neurons, triggering the PI3K/Akt and mitogen-activated protein kinases (MAPKs) signaling pathways that further reinforce the tumor-supportive signaling [122]. In metastatic settings, breast cancer-derived EVs carrying miR-199b-5p disrupt neuron-astrocyte metabolic coupling and promote tumor growth [123].

Neuron-tumor interactions also shape the immunosuppressive BTME. Gliomas inhibit natural killer (NK) cell and CD8^+^ T cell functions via the N-acetyltransferase 8-like (NAT8L)/N-acetylaspartate (NAA) axis and disrupt immune synapses through laminin acetylation [124], [125]. TAMs phagocytose myelin lipids and form immunosuppressive lipid-laden macrophages that transfer lipids to tumor cells through the liver X receptor (LXR)/ATP-binding cassette transporter A1 (Abca1) pathway [126]. Neurofibromatosis 1 (*NF1*)-mutant neurons induce microglia to produce C‑C motif chemokine ligand 5 (CCL5), which supports GSC survival and tumor growth [127]. Moreover, *NF1* mutations induce neuronal hyperexcitability and intermediate factor expression, which activate immune axes to support the growth of NF1-associated optic gliomas [128]. Additionally, calcium/calmodulin‑dependent protein kinase kinase 2 (CaMKK2) maintains immunosuppressive microglial phenotypes and limits T cell infiltration in GBM [129] **(**Fig. 3**)**.

Collectively, these findings define a malignant “neuron-tumor” cycle that sustains tumor growth, invasion, immune suppression, and treatment resistance [108], [109], [130]. Preclinically employed therapeutic strategies involve targeting the GABA/NKCC1 signaling pathway [114], inhibiting the NAT8L/NAA metabolic axis [124], [125], and employing rabies virus-based drugs for tumor-specific neuronal ablation [131]. Notably, interventions targeting the USP1/NgR1 pathway [95] and the miR-184-3p/RBM15/DLG3 axis [99] could overcome treatment resistance. Elucidating the neuron-tumor interaction patterns across tumor will be essential for developing precision therapies and neuroprotective strategies, offering substantial translational medical value.

### Astrocytes

Astrocytes are the predominant glial cells in the CNS and play critical roles in brain homeostasis, neuronal function, and neurological disorders. The concept of the astrocyte-neuron lactate shuttle (ANLS) originated from the 1994 proposal that astrocytes exhibit elevated glycolytic activity, producing and secreting lactate, which is subsequently taken up by neurons and oxidized in mitochondria to support neuronal energy requirements [132], [133].

In 2012, Barres classified reactive astrocytes into two functional states, A1 (neurotoxic) and A2 (neuroprotective) [134], [135], [136]. A1 astrocytes secrete inflammatory factors and neurotoxins, whereas A2 astrocytes upregulate neurotrophic factors. The A1/A2 astrocyte classification is commonly widely applied to neurodegenerative diseases such as Alzheimer’s disease, Parkinson’s disease, Huntington’s disease, amyotrophic lateral sclerosis, and multiple sclerosis, and has been refined into additional subpopulations [136]. In contrast, astrocytes in brain tumors are generally considered in an activated state and are classified based on surface markers or signaling markers, such as pSTAT3^+^, JAG1^+^, Annexin A1 (ANXA1)^+^, and TNF-related apoptosis-inducing ligand (TRAIL)^+^ [137], [138], [139].

Astrocytes are attracted to and activated by tumor cells, thereby promoting tumor formation within the BTME, characterized by altered cytokine secretion, metabolic adaptation, and dynamic interactions with tumor cells [140], [141], [142]. Activated astrocytes secrete cytokines such as IL-6 and transforming growth factor-β (TGF-β), which activate the Janus kinase (JAK) signal transducer and activator of transcription (JAK-STAT) and Smad pathways to promote tumor progression [140], [141]. Notably, tumor-associated astrocytes (TuAstrocytes) in the medulloblastoma originate from granule neuron precursors and drive tumor growth through the IL-4/insulin-like growth factor 1 (IGF1) axis [140], [141]. In BrMs from small cell lung cancer, tumor-derived Reelin recruits astrocytes, which subsequently facilitate metastatic growth by producing neuronal survival factors such as serpin family E member 1 (SERPINE1) [143]. Additionally, 2’3’-cyclic GMP-AMP (cGAMP) has been shown to recapitulate early metabolic alterations in astrocytes induced by BrMs [144]. The integrin-linked kinase (ILK)/STAT3 signaling pathway also regulates GSCs’ plasticity, enabling their transdifferentiation into astrocyte-like cells [145].

Astrocytes contribute to tumor immune escape through multiple mechanisms. Cyclin-dependent kinase 5 (CDK5) suppresses major histocompatibility complex class Ⅰ (MHC-Ⅰ) expression via the Ifn regulatory factor 2-binding protein 1 (IRF2BP1)-signal transducer and activator of transcription 1 (STAT1)-importin α-NLR family CARD domain containing 5 (NLRC5) pathway [146], while astrocyte-derived IL-11 upregulates PD-L1 to promote immune escape [147]. Priego *et al*. [142] demonstrated that pSTAT3^+^ astrocytes suppress CD8^+^ T cell function via tissue inhibitor of metalloproteinases 1 (TIMP1) secretion. Furthermore, lipocalin-2 (LCN2)-mediated astrocyte recruits myeloid cells, exacerbates neuroinflammation, and promotes BrMs [143].

Over the past decade, increasing attention has been devoted to the role of astrocytes in tumors. As key components of the BBB, astrocytes can initially restrain tumor growth and help maintain brain homeostasis during early tumorigenesis. However, as tumors progress, astrocytes are hijacked by tumor cells, contributing to BBB remodeling within the BTME and acquiring pro-tumorigenic functions [148], [149]. Despite extensive mechanistic insights, clinical translation has remained limited. Notably, STAT3 inhibitors, including silibinin and WP1066, represent a promising therapeutic strategy in brain tumor and are currently under investigation in clinical trials (NCT01904123, NCT05689619).

### Diversity of immune cell populations

The BTME comprises diverse immune cells, including microglia, macrophages, T cells, B cells, and NK cells, which exert complex and contradictory roles in immune surveillance and escape. Extensive remodeling of the BTME creates a highly immunosuppressive and fibrotic niche [150], [151]. Gonzalez *et al*. [152] identified two functional BrM prototypes driven by tumor-immune interactions. In the BTME, antigen presentation is impaired and B- and T-cell activity is reduced, while infiltration of neutrophils and M2-polarized macrophages is increased [150], [151], [152]. Notably, in breast cancer BrM, only 3.8% of tumors are classified as “hot” tumors characterized by high immune infiltration, whereas 73.1% are classified as “cold” tumors [50]. Melanoma BrMs display a profoundly immunosuppressive state characterized by decreased interferon‑γ (IFN-γ), tumor necrosis factor (TNF), and IL-12B expression and T cell inflammatory scores [153].

Technological advancements have led to the identification of more refined pathological subtypes of cells or ligand-receptors in BTME, which promote tumors development. Forkhead box protein P3 (FOXP3)^+^ Tregs, lysosome-associated membrane protein 3 (LAMP3)^+^ tolerogenic dendritic cells (DCs), CCL18^+^ M2-like macrophages, regulator of G protein signaling 5 (RGS5)^+^ CAFs, and galectin 1(LGALS1)^+^ microglia are notably enriched in the BrM microenvironment [154]. These cells collectively establish immune tolerance by secreting immunosuppressive mediators and modulating immune checkpoint molecule expression. Importantly, the canonical PD-1/PD-L1 axis is largely absent in BrMs, whereas alternative immune checkpoint pathways, including lymphocyte activating 3 (LAG3)-LGALS3 and T cell immunoreceptor with Ig and ITIM domains (TIGIT)- nectin cell adhesion molecule 2 (NECTIN2), play dominant roles in regulating interactions between CD8^+^ T cells and tumor or stromal cells [154].

#### Dendritic cells

**Fig. 4:**
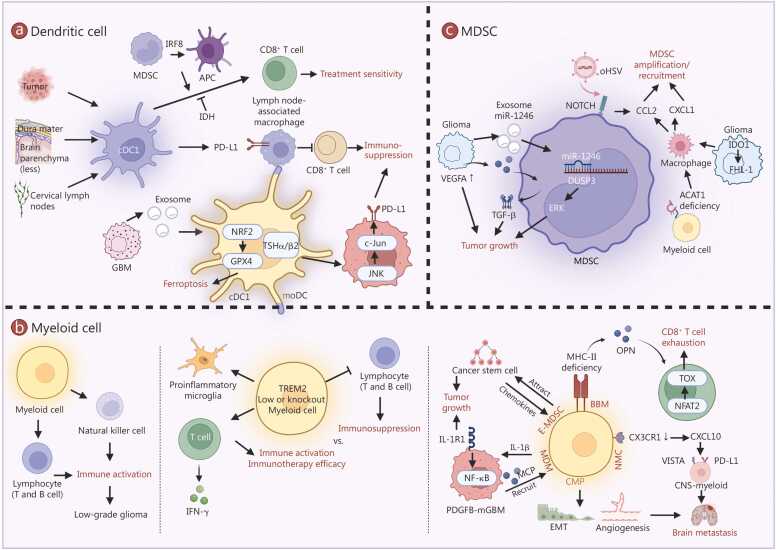
**Multifaceted roles of DCs and myeloid subsets in regulating brain tumor immunity. a** DCs in brain tumors. cDC1s are rare in the brain parenchyma and are mainly localized to the meninges and draining lymph nodes. In lymph nodes, DCs predominantly stimulate immunosuppressive PD‑L1^+^ LAMs. IRF8 is a critical regulator of cDC1 development, and its delivery can reprogram intratumoral MDSCs into antigen-presenting cells. Glioma-driven immunosuppression impairs DC function via multiple mechanisms, including *IDH* mutations reprogram monocytes, GBM-derived exosome-induced ferroptosis via the NRF2/GPX4 pathway, and aberrant PD-L1 upregulation in monocyte-derived DCs through TSHα/β2-JNK-c-Jun signalling. **b** Functional heterogeneity of myeloid cells. In low-grade gliomas, myeloid cells exhibit an immune-activated phenotype accompanied by increased T cell and NK-cell infiltration, whereas in GBM, they acquire immunosuppressive states characterized by TREM2 downregulation, T cell exhaustion, and reduced immunotherapy efficacy. Loss of TREM2 enhances IFN-γ-induced immune activation and sensitizes tumors to PD-1 blockade. GSCs recruit E-MDSCs via chemokine secretion, which in turn releases growth factors that support tumor progression. A genotype-dependent IL-1β-IL-1R1 paracrine loop between tumor cells and myeloid cells drives PDGFB-driven GBM progression through NF-κB activation and MCP upregulation. In BBM cells, loss of MHC‑Ⅱ induces dysfunctional tumor-reactive CD8^+^ T cells by increasing TOX expression. Conversely, MHC-Ⅱ-dependent CD4^+^ T cell activation restrains OPN production by myeloid cells, preventing chronic NFAT2 activation in CD8^+^ T cells. In BrM, CMPs restrain brain colonization, while CNS-resident myeloid cells promote metastatic outgrowth through interferon signalling and recruitment of VISTA^high^ PD-L1^+^ myeloid populations. **c** Recruitment and expansion of MDSC. VEGFA or GBM-derived exosomal miR-1246 promotes MDSC differentiation and stimulates TGF‑β1 secretion, reinforcing tumor-promoting phenotypes. Following oHSV therapy, NOTCH signalling drives MDSC recruitment via the upregulation of the NOTCH ligand Jag1. NOTCH-activated macrophages further secrete CCL2, amplifying the accumulation of MDSCs. The loss of ACAT1 facilitates MDSC expansion and contributes to GBM progression. Tumor-intrinsic IDO enhances immune suppression independently of tryptophan metabolism by upregulating CPH and FHL‑1, leading to increased intratumoral regulatory T cells (Tregs) and MDSC abundance. DCs. Dendritic cells; cDC1s. Conventional dendritic cell type 1; PD-L1. Programmed death-ligand 1; LAMs. Lymph node-associated macrophages; IRF8. Interferon regulatory factor 8; MDSCs. Myeloid-derived suppressor cells; IDH. Isocitrate dehydrogenase; GBM. Glioblastoma; NRF2. Nuclear factor erythroid 2-related factor 2; GPX4. Glutathione peroxidase 4; TSH. Thyroid-stimulating hormone; JNK. c-Jun N-terminal kinase; NK. Natural killer; TREM2. Triggering receptor expressed on myeloid cells 2; IFN-γ. Interferon-γ; GSCs. Glioma stem cells; IL. Interleukin; PDGFB. Platelet-derived growth factor subunit B; NF-κB. Nuclear factor κB; MCP. Monocyte chemoattractant protein; MHC-Ⅱ. Major histocompatibility complex class Ⅱ; TOX. Thymocyte selection-associated high mobility group box protein; NFAT2. Nuclear factor of activated T cells 2; BrM. Brain metastases; CNS. Central nervous system; VISTA. V-domain immunoglobulin suppressor of T cell activation; VEGFA. Vascular endothelial growth factor A; TGF-β1. Transforming growth factor-β1; oHSV. Oncolytic herpes simplex virus; NOTCH. Neurogenic locus notch homolog; CCL2. C-C motif chemokine ligand 2; ACAT1. Acetyl-CoA acetyltransferase 1; IDO. Indoleamine 2,3-dioxygenase; CPH. Carboxypeptidase H; FHL-1. Four and a half LIM domains 1; Tregs. Regulatory T cells; BBM. Blood-borne myeloid; OPN. Osteopontin; CMP. Circulating myeloid progenitors; E-MDSC. Early progenitor MDSCs; APC. Antigen-presenting cells; CXCL1. C-X-C motif chemokine ligand 1; DUSP3. Dual specificity phosphatase 3; ERK. Extracellular regulated protein kinases; CX3CR1. C-X3-C motif chemokine receptor 1; EMT. Epithelial-mesenchymal transition; MDM. Monocyte-derived macrophages.

As the most potent antigen-presenting cells (APCs), Dendritic cells (DCs) are present in both primary and recurrent high-grade gliomas **(**Fig. 4**a)**. Conventional type 1 DC (cDC1) cells are essential for initiating extracranial CD8^+^ T cell-mediated anti-tumor responses. However, cDC1s are exceedingly rare in the steady-state brain parenchyma and are primarily detected in tumor lesions, the dura mater, and cervical lymph nodes [155]. Despite their low abundance, cDC1s contribute to therapeutic benefits when combined with checkpoint blockade [155].

The critical challenge to effective immunotherapy in GBM or glioma is not solely the intrinsic immune-suppressive BTME but also defective antigen presentation. Dysfunctional DCs severely limit the antigen-specific T cell response. For example, *IDH* mutations in gliomas reprogram infiltrating monocytes via paracrine signaling, which leads to functionally impaired DCs with specific antigen presentation defects [156]. In the lymph nodes, DCs fail to effectively activate CD4^+^/CD8^+^ T cells and instead promote immune suppression by stimulating PD-L1^+^ lymph node-associated macrophages (LAMs) [157]. For example, GBM-derived exosomes induce ferroptosis in DCs through the nuclear factor erythroid 2-related factor 2 (NRF2)/GPX4 pathway, which weakens their antigen presentation function [158] **(**Fig. 4**a)**. Collectively, these alterations lead to defective DC-mediated immune priming and contribute to immune escape.

Therapeutic strategies targeting DCs have demonstrated promising clinical potential [159], [160]. In a phase Ⅱ clinical trial, DC vaccine AV-GBM-1 targeting tumor-initiating cells significantly prolonged median progression-free survival (PFS) [160]. A phase Ⅲ trial showed that a DC vaccine loaded with autologous tumor lysate extended the median overall survival (OS) of newly diagnosed GBM (ndGBM) patients to 19.3 months [161]. Various engineering strategies have been developed to enhance the DCs’ anti-tumor efficacy [162], including the activation of Toll-like receptor (TLR) agonists [163], reprogramming of myeloid-derived suppressor cells (MDSCs) into APCs via interferon regulatory factor 8 (IRF8) overexpression [164], and nanotechnology-based strategies to promote DC maturation [165]. Additionally, machine learning-guided reprogramming of glioma cells into DC-like APCs (iDC-APCs) has generated cells with functional properties comparable to those of natural DCs [166].

DC-targeted therapies exhibit enhanced efficacy when combined with other immunotherapeutic approaches [160]. Tumor-associated macrophages nano-reprogrammers (MG5-S-IMDQ) improves anti-tumor immunity by increasing cDC1 infiltration in GBM [167]. Chemotherapy-derived lipid nanoparticles combined with Fms-like tyrosine kinase 3 ligand (*Flt3L*)/CD40 ligand (*CD40L*) mRNAs promote DC activation and BTME reprogramming [168]. Synergistic effects have also been observed with PD-L1 blockade and DC vaccines by depleting PD-L1^+^ macrophages [157], as well as with DC-cytokine-induced killer (DC-CIK) cell therapy [169]. These findings lay the foundation for developing personalized DC-based combination therapies, including *IDH* mutation-specific strategies [156], integration with immune checkpoint inhibitors [157], and novel nanoparticle delivery systems [170]. Overcoming the BBB and optimizing the antigen presentation efficiency of DCs remain key challenges, with innovative technologies offering promising solutions [166].

#### Myeloid cells

Myeloid cells display considerable heterogeneity and functional diversity [171], [172]. Classically activated M1 macrophages and mature DCs display anti-tumor activity, whereas TAMs and MDSCs promote immune suppression, angiogenesis, metastasis, and treatment resistance. However, in low-grade gliomas, myeloid cells exhibit a more immune-activated phenotype, accompanied by increased infiltration of CD4^+^/CD8^+^ T cells and NK cells [173]. Myeloid cells exhibit four immune regulatory programs, including microglial inflammation and phagocytic immune suppression, which are specific to brain tumors, as well as systemic inflammation and complement immune suppression, which are also observed in extracranial tumors. These programs are predominantly influenced by microenvironmental signals, including hypoxia, IL-1β, TGF-β, and dexamethasone treatment [172].

Myeloid cells promote tumor progression by reshaping the BTME through crosstalk with tumor cells and modulation of anti-tumor immune responses [174], [175]. For example, GSCs recruit early progenitor MDSCs (E-MDSCs) by chemokine secretion, which subsequently release growth factors to support tumor growth [171]. In platelet-derived growth factor subunit B (PDGFB)-driven GBM, a highly active IL-1β/IL-1R1 paracrine loop further illustrates this bidirectional interaction: monocyte-derived macrophages (MDMs) secrete IL-1β, activating the NF-κB signaling in tumor cells, and inducing the expression of monocyte chemoattractant proteins (MCPs) [176]. MHC-Ⅱ deficiency in blood-borne myeloid cells drives CD8^+^ T cell exhaustion by increasing chromatin accessibility and Tox expression, a process involving chronic activation of myeloid cell-derived osteopontin and nuclear factor of activated T cells 2 (NFATC2) in CD8^+^ T cells [177]. In BrMs, CNS-resident myeloid cells downregulate C-X3-C motif chemokine receptor 1 (CX3CR1), which leads to enhanced interferon responses and C-X-C motif chemokine ligand 10 (CXCL10) upregulation, thereby potentially influencing immune cell recruitment within the metastatic niche [178] **(**Fig. 4**b)**.

Therapeutic targeting of myeloid cells has demonstrated substantial potential [179], [180]. Triggering receptor expressed on myeloid cells 2 (TREM2) is upregulated in brain tumors and TREM2 deficiency enhances IFN-γ-induced immune activation and improves efficacy of anti-PD-1 therapy [175]. In addition, binding of CNS-enriched sphingolipids to TREM2 on myeloid cells induces anti-tumor responses in GBM [174]. Conversely, Zheng *et al*. [181] reported that TREM2 is downregulated in GBM-infiltrating myeloid cells, where TREM2 promotes tumor cells’ phagocytosis and enhances immune responses by facilitating MHC-Ⅱ-associated CD4^+^ T cell responses. Preclinically, engineered bone-marrow-derived myeloid cells that release IL-2 (GEMys-IL2) show therapeutic efficacy in low-grade gliomas by promoting T and NK cell recruitment [182]. These findings support the development of precision immunotherapy strategies that target various myeloid cell subpopulations [172]. Preclinical studies indicate that many strategies enhance therapeutic responses by modulating myeloid cells [182]. However, effective myeloid cell-targeted therapeutic strategies remain largely undeveloped. Dynamic monitoring of myeloid cell composition, such as the predominance of microglia-derived TAMs in primary tumors and monocyte-derived TAMs in recurrent hypoxic lesions, provides insights for optimizing treatment timing and strategy selection [180].

#### Myeloid-derived suppressor cells

Myeloid-derived suppressor cells (MDSCs) are a heterogeneous population of immature myeloid cells that expand under pathological conditions such as cancer, infections, and inflammation [183] **(**Fig. 4**c)**. MDSCs potently suppress the anti-tumor functions of T cells and NK cells through the production of immunosuppressive mediators like ARG1, inducible nitric oxide synthase (iNOS), reactive oxygen species (ROS), and cytokines like IL-10 and TGF-β, while also promoting angiogenesis and tumor metastasis [184]. *IDH* mutation status significantly influences MDSC abundance, with reduced inhibitory myeloid cells in *IDH*-mutant gliomas [185]. The single-cell sequencing has identified two major MDSC subsets in IDH wild-type GBM, metabolically active E-MDSCs and monocytic MDSCs (M-MDSCs). Notably, E-MDSCs localize near metabolic stem-like tumor cells within pseudopalisading regions [171], [186], [187]. High expression of integrin β1 and dipeptidyl peptidase-4 (DPP-4) enhances the adhesion and tumor-homing capacity of M-MDSCs [188]. Tumor cells actively recruit MDSCs through various mechanisms, including VEGF-containing exosomes that promote MDSC differentiation and TGF-β1 secretion [189], macrophage-derived CCL2 [190], and hypoxia-induced miR-1246-mediated dual specificity phosphatase 3 (DUSP3)/ extracellular signal regulated kinases (ERK) pathway [191].

Myeloid cell-associated metabolic and accumulation of immunoregulatory alterations further contribute to brain tumor progression [186], [187]. Acetyl-CoA acetyltransferase 1 (ACAT1) deficiency in myeloid cells promotes fatty acid oxidation, which accumulates MDSC and promotes GBM progression [192]. In addition, indoleamine 2,3-dioxygenase 1 (IDO) upregulates factor H-like protein 1 (FHL-1), which is associated with increased infiltration of Tregs and MDSCs, leading to reduced overall survival (OS) in GBM-bearing mice [193]. Moreover, leukocyte immunoglobulin like receptor B2 (LILRB2) in GBM cells inhibits the function of CD8^+^ T cells and the expansion of MDSCs, which constructs an immunosuppressive BTME and promotes GBM [194] **(**Fig. 4**c).**

Myeloid-directed strategies are being explored to improve immunotherapy. Preclinical studies have shown that IL-2 can enhance brain tumor treatment outcomes by inhibiting myeloid cells [195], [196], but myeloid cell-based therapeutic strategies remain underdeveloped in clinical practice. Current MDSC-targeted approaches generally focus on inhibiting the generation or expansion of MDSCs, depleting existing MDSCs, preventing their recruitment, and modulating their immunosuppressive functions [30].

#### Microglia

**Fig. 5:**
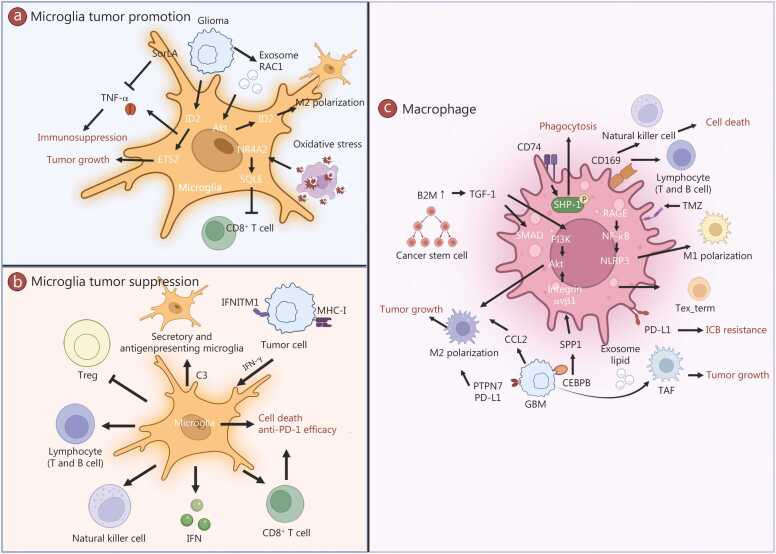
**Functional plasticity of microglia and TAMs in brain tumors. a** The ID2-ETS2 axis promotes the polarization of microglia towards a tumor-supportive phenotype. SorLA, a neuronal trafficking receptor, is repurposed in the GBM microenvironment to reprogram GAMs by suppressing TNF‑α release, thereby suppressing anti-tumor immunity. Under oxidative stress, NR4A2-dependent transcriptional reprogramming drives alternative activation of microglia and inhibits CD8^+^ T cell responses. Furthermore, GBM-derived exosomes carrying RAC1 promote M2-like microglial polarization through the protein Akt/NRF2 signalling pathway. **b** Despite immunosuppressive roles, microglia can also facilitate anti-tumor immunity. In breast cancer BrM, microglia enhance interferon signalling, increase cytotoxic CD4^+^ and CD8^+^ T cell infiltration, and reduce Tregs accumulation. Early brain metastatic cancer cells with high IFITM1 expression stimulate microglial C3 production, which increases MHC‑I expression and membrane localization, promoting CD8^+^ T cell-mediated cytotoxicity. **c** Regulatory role of TAMs in the BTME. B2M interacts with PIP5K1A in GSCs, inducing TGF‑β1 secretion and activating the PI3K/Akt signalling pathway, which promotes M2-like macrophage polarization. The CEBPB transcriptional network defines a tumorigenic GBM subcluster by coordinating macrophage recruitment via MCP1 and M2 polarization through the SPP1-integrin αvβ1-Akt pathway. TAFs arise through lipid uptake from GBM-derived extracellular vesicles, which promote tumor growth. PTPN7 actively mediates inflammation resolution and the polarization of macrophages and microglia, protecting gliomas from immune attacks. TAMs, rather than tumor cells, serve as the primary source of antigen exposure, controlling the transition of Tex_prog_ cells to Tex_term_ cells. TMZ-induced HMGB1 via the RAGE-NF-κB-NLRP3 pathway promotes M1-like polarization, enhancing chemotherapy sensitivity. APP, through its interaction with CD74, promotes the phosphorylation of SHP‑1, inhibiting the phagocytic activity of TAMs. CD169^+^ macrophages produce proinflammatory chemokines, facilitating the accumulation of T cells and NK cells. TAMs. Tumor-associated macrophages; ID2. Inhibitor of DNA binding 2; ETS2. ETS proto-oncogene 2; SorLA. Sortilin-related receptor; GBM. Glioblastoma; GAMs. Glioma-associated microglia and macrophages; TNF-α. Tumor necrosis factor-α; NR4A2. Nuclear receptor subfamily 4 group A member 2; RAC1. Ras-related C3 botulinum toxin substrate 1; Akt. Protein kinase B; NRF2. Nuclear factor erythroid 2-related factor 2; BrM. Brain metastases; Tregs. Regulatory T cells; IFITM1. Interferon-induced transmembrane protein 1; C3. Component 3; MHC-Ⅰ. Major histocompatibility complex class I; BTME. Brain tumor microenvironment; B2M. β2-microglobulin; PIP5K1A. Phosphatidylinositol-4-phosphate 5-kinase type 1 alpha; GSCs. Glioma stem cells; TGF-β1. Transforming growth factor-β1; PI3K. Phosphoinositide 3-kinase; CEBPB. CCAAT enhancer binding protein beta; MCP1. Monocyte chemoattractant protein 1; SPP1. Secreted phosphoprotein 1; TAFs. Tumor-associated foam cells; PTPN7. Protein tyrosine phosphatase non-receptor type 7; Tex_prog_. Progenitor-exhausted T; Tex_term_. Terminally exhausted T; TMZ. Temozolomide; HMGB1. High mobility group box 1; RAGE. Receptor for advanced glycation end products; NF-κB. Nuclear factor κB; NLRP3. NLR family pyrin domain containing 3; APP. Amyloid precursor protein; SHP-1. Src homology region 2 domain-containing phosphatase 1; NK. Natural killer.

Microglia, the resident immune cells of the CNS, are predominant in the BTME. In the BTME, microglia are often polarized into a protumor M2 phenotype, driven by several molecular mechanisms [34]. In GBM, a microglia-glioblastoma specific phenotype suppresses immune responses and promotes tumor cell proliferation [197]. The inhibitor of DNA binding 2 (ID2)-ETS proto‑oncogene 2 (ETS2) signaling axis of microglia regulates the protumoral phenotype in GBM [198]. Additionally, sortilin-related receptor (SorLA) on tumor cells reprograms glioma-associated microglia/macrophages by restricting tumor necrosis factor-α (TNF-α) release, which inhibits anti-tumor capabilities [199]. Finally, oxidative stress induces nuclear receptor subfamily 4 group A member 2 (NR4A2)-dependent transcriptional reprogramming in microglia, which inhibits CD8^+^ T cell activity [200] **(**Fig. 5**a, b)**.

Under certain conditions, microglia exhibit anti-tumor effects. For example, GBM organoid models have shown that tumor-associated microglia display distinct phagocytic and DC-like characteristics [201]. In breast cancer BrMs, microglia suppress metastatic growth by upregulating proinflammatory programs, including interferon responses, antigen presentation, and cytokine secretion, thereby enhancing CD4⁺ T cell, CD8⁺ T cell, and NK-cell responses within the CNS [202]. IFN-induced transmembrane protein 1 (IFITM1) mediates metastasis and treatment resistance in various tumors [203]. However, IFITM1 in BrM activates microglia and releases complement component 3, leading to CD8^+^ T cell activation and inhibiting tumor progression [204]. Consistently, targeting NR4A2 or CX3CR1^+^ myeloid cell-specific knockout enhances the antigen-presenting capacity of microglia, thereby promoting anti-GBM immunity [200] **(**Fig. 5**a, b)**.

While some studies have reported inhibitory effects of microglia on brain tumors, the predominant therapeutic strategy remains suppressing microglia-mediated protumor functions [205], [206]. Targeting the heat shock protein 47 (HSP47)-collagen axis, such as with the HSP47 inhibitor COL003, can reverse M2 polarization of microglial cells, restore anti-tumor immune activity, and inhibit BrM growth [207]. Targeting CD72 significantly reduce the number of Tregs and microglia can suppress brain tumor [208]. The CD73 inhibitor AB680 blocks the conversion of adenosine monophosphate (AMP) to immunosuppressive adenosine, thereby promoting an anti-tumor M1-like phenotype in purinergic receptor P2Y12 (P2RY12)^+^ microglia and enhancing tumor immune responses [209]. In addition, dietary modulation of butyrate levels to enhance the anti-tumor activity of microglia represents a potential complementary approach [210].

#### Tumor-associated macrophages

Tumor-associated macrophages (TAMs) are a major immune cell population in brain tumor. In *IDH*-mutant gliomas, there is a high density of TAMs and a relatively low abundance of T cells. This supports the classification of gliomas as immune “cold” tumors [211].

Notably, monocyte-derived TAMs represent the predominant population and are preferentially polarized toward the M2 phenotype within the BTME [211]. This phenotype is further reinforced by tumor-derived signals. For example, β2-microglobulin (B2M), a component of MHC-Ⅰ, promotes tumor stemness and reprograms the tumor immune microenvironment (TIME) to an anti-inflammatory state [212]. Mechanistically, B2M-induced TGF-β1 secretion activates paracrine SMA and MAD related family (SMAD) and PI3K/Akt signaling in TAMs, driving the M2 macrophage phenotype [212]. In addition, the CCAAT/enhancer-binding protein beta (CEBPB) transcriptional network regulates monocyte chemoattractant protein-1 (MCP1)-mediated macrophage recruitment and activates the SPP1-integrin αvβ1-Akt pathway in GBM, thereby supporting tumor-promoting macrophage programs [213]. Notably, the traditional M1/M2 dichotomy does not fully capture TAM diversity [214], as subpopulations such as CD169^+^ TAMs have proinflammatory characteristics [215], while tumor-associated foam cells (TAFs) present distinct protumor features [216] **(**Fig. 5**c)**.

TAMs promote BTME immunosuppression through multiple mechanisms. For example, protein tyrosine phosphatase non-receptor type 7 (PTPN7) in glioma mediates macrophage polarization and protects gliomas from immune attack [217], [218]. In addition, the MET/STAT4/PD-L1 pathway further enhances the immunosuppressive function of TAMs [219], whereas the amyloid protein precursor (APP)-CD74 axis protects GBM cells from TAM phagocytosis by promoting Src homology region 2 domain-containing phosphatase-1 (SHP-1) phosphorylation, which finally leads to a poorer prognosis [220]. Compared with terminally exhausted T (Tex_term_) cells, stem-like progenitor-exhausted T (Tex_prog_) cells have better immunotherapeutic responsivity. However, TAMs usually shape dysfunctional T cell responses by serving as a major source of antigen exposure that drives the transition of Tex_prog_ cells to Tex_term_ cells, which promote GBM [221]. Furthermore, TAMs contribute to treatment resistance. Even after anti-PD-1 treatment, macrophages and monocytes remain predominant among infiltrating immune cells, while sustained high expression of T cell inhibitory checkpoints prevents effective activation of tumor-infiltrating lymphocytes (TILs) [222]. TMZ-induced high mobility group box 1 (HMGB1) promotes M1-like macrophage polarization via the RAGE-NF-κB-NLRP3 pathway, which enhances chemotherapy sensitivity in GBM [223] **(**Fig. 5**c)**.

Therapeutic strategies targeting TAM plasticity have shown significant promise. Colony-stimulating factor 1 receptor (CSF1R) inhibitors, such as PLX3397 and GW2580, reprogram M2-like GBM-associated macrophages and impair tumor proliferation [224]. Anti-miR-25/cGAMP nanocomplexes activate the cyclic GMP-AMP synthase- stimulator of interferon gene (cGAS-STING) pathway to promote M1 polarization of macrophages, which enhances immune responses in GBM [225]. Targeting membrane spanning 4-domains A4A (MS4A4A) and combining immune checkpoint blockade (ICB) therapy drives the macrophage conversion of M2 to M1, leading to complete tumor eradication [226]. Current treatment strategies are often based on the classic phenotypic transition between M1 and M2 [220]. Future research will investigate specific subsets, functional states, spatial distributions, and how TAMs connect with tumor cells and other immune cells in the BTME, and promote brain tumor progression and immunosuppression [34].

#### T cells

Under physiological conditions, T cells patrol the CNS to control latent infections, highlighting their critical role in maintaining CNS homeostasis. Activated T cells enter the CNS through three main pathways [227], migration to the choroid plexus and traversal of the blood-cerebrospinal fluid barrier; extravasation across meningeal venules into the subarachnoid space; and direct crossing of the BBB at post-capillary venules to access the parenchyma. In BrMs, specific venous vessels have been identified as key structures for T cell recruitment [228]. To reach the parenchyma or tumor sites, T cells enter the perivascular space and then penetrate the glial limitans formed by astrocytic endfeet. Whether T cells can directly cross the ventricular ependymal layer adjacent to brain tumors remains unclear.

In brain tumors, T cells, particularly cytotoxic T lymphocytes (CTLs), are the principal effector cells of anti-tumor immunity. Their infiltration varies markedly. In gliomas, T cells account for about 1%–10% of immune infiltrates in GBM but only 0.1%–1% in astrocytomas and oligodendrogliomas [229], [230]. Compared to gliomas, BrMs show relatively higher T cell infiltration.

**Fig. 6:**
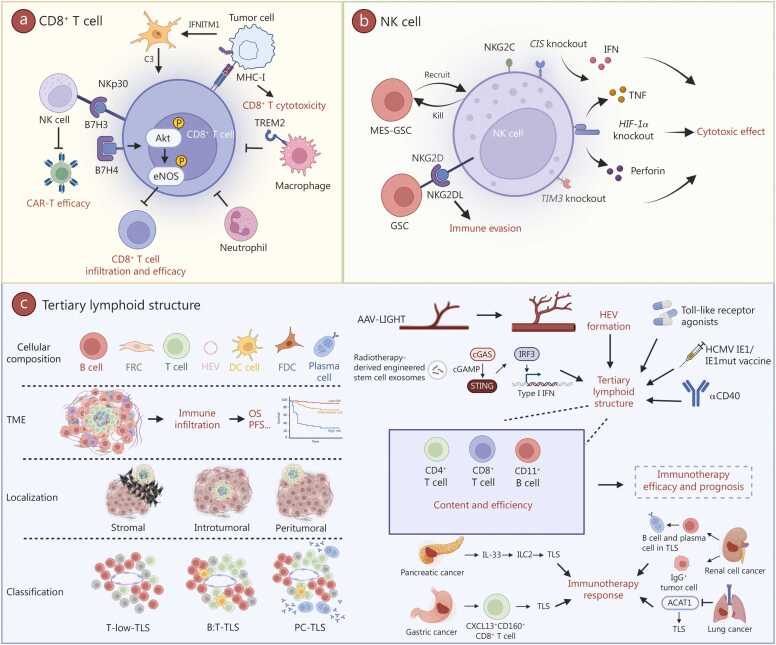
**Immune landscape and therapeutic reprogramming in brain tumors: from T and NK cell dynamics to TLS-driven immunity. a** CD8^+^ T cell states and regulatory interactions in brain tumors. Unlike classical checkpoint molecules, B7-H6 mediates NKp30-dependent recognition and subsequent cytolysis of activated T cells by NK cells. In humanized mouse models, B7-H6-mediated NK cell surveillance limits the persistence and anti-tumor activity of CAR‑T cells. B7-H4 induces early dysfunction of CD8^+^ TILs by downregulating Akt and eNOS phosphorylation, reducing T cell infiltration and cytotoxicity in gliomas. Neutrophils and TREM2-expressing macrophages are key sources of local T cell suppression within the brain, contributing to immune escape in the CNS. Early brain metastatic cancer cells with high expression of IFITM1 activate microglia through the secretion of complement C3 and enhance CD8^+^ T cell cytotoxicity by increasing MHC‑I. **b** Strategies to enhance NK cell cytotoxicity against brain tumors. Knockout of *CIS* enhances the production of IFN and TNF, thereby promoting NK cell-mediated cytotoxicity in GBM cells. In a hypoxic microenvironment, knockout of *HIF-1α* increases cytotoxicity through increased perforin and TNF expression, further augmenting anti-tumor activity. *TIM3* knockout enhances NK cell cytotoxicity against glioma cells. MES-GSCs accumulate NK cell ligands and are sensitive to NK-mediated cytotoxicity *in vitro*. EZH2-encoded EZH2-92aa suppresses surface NKG2D ligands, facilitating immune escape. Additionally, NKG2C^+^ NK cells show therapeutic potential. **c** TLSs and their contribution to anti-tumor immunity. The composition, location, and classification of tertiary lymphoid structures are quite complex. The degree of TLS infiltration often indicates better PFS and OS. In glioma, vascular expression of LIGHT can induce the formation of HEVs and TLSs. Radiation-derived engineered stem cell-derived exosomes activate the cGAS-STING pathway, promoting the formation of TLSs, increasing type Ⅰ interferon release and enhancing TLS formation. TLS formation is associated with increased lymphocyte infiltration in the BTME, and Toll-like receptor agonists promote the formation of TLSs. Systemic delivery of immunostimulatory agonistic αCD40 in preclinical glioma models induces TLS formation near the meninges. The systemic delivery of αCD40 induces impaired T cells, and the mechanism involves the systemic induction of suppressive CD11b^+^ B-cell infiltration. HCMV IE1/*IE1*mut vaccination preclinically results in significantly more and larger TLSs. TLS is also related to immunotherapy responses in extracranial research. NK. Natural killer; B7-H6. Natural killer cell cytotoxicity receptor 3 ligand 1; NKp30. Natural killer cell protein 30; CAR-T. Chimeric antigen receptor T; B7-H4. V-set domain containing T cell activation inhibitor 1; TILs. Tumor-infiltrating lymphocytes; Akt. Protein kinase B; eNOS. Endothelial nitric oxide synthase; TREM2. Triggering receptor expressed on myeloid cells 2; CNS. Central nervous system; IFITM1. Interferon-induced transmembrane protein 1; C3. Complement component 3; MHC-Ⅰ. Major histocompatibility complex class I; GBM. Glioblastoma; CIS. Cytokine-inducible SH2-containing protein; IFN. Interferon; TNF. Tumor necrosis factor; HIF. Hypoxia-inducible factor; TIM3. T cell immunoglobulin and mucin domain-containing protein 3; GSCs. Glioma stem cells; circular EZH2. Circular RNA enhancer of zeste homolog 2; NKG2D. NK group 2D; NKG2C. NK group 2C; TLSs. Tertiary lymphoid structures; HEVs. High endothelial venules; cGMP. Cyclic GMP; cGAS. cGMP-AMP synthase; STING. Stimulator of interferon genes; TME. Tumor microenvironment; BTME. Brain tumor microenvironment; HCMV. Human cytomegalovirus.

Beyond overall abundance, T cell infiltration in brain tumors exhibits pronounced spatial specificity [231]. A population of lymphocyte-like cells residing in the calvarium has been shown to coexist with GBM. Compared to distal bone marrow, the tumor-adjacent calvarial microenvironment contains a higher proportion of tumor-reactive CD8^+^ effector cells characterized by sphingosine 1-phosphate receptor 1 (S1PR1) expression [232]. A significant clonal overlap exists between proliferative and exhausted CD8^+^ T cells, while clonally distinct CD8^+^ T cell populations with divergent phenotypes occupy different niches within the BrM microenvironment [231] **(**Fig. 6**a)**. Notably, even in BrMs that respond more favourably to immune checkpoint inhibitors (ICIs), such as those from melanoma and non-small cell lung cancer (NSCLC), immune infiltration often remains spatially constrained, predominantly localized to perivascular regions or tumor margins. This intracranial response to ICIs is associated with the expansion of peripheral effector CD8^+^ T cells with homing capacity [233].

The functional states of CD4^+^ T cells differ significantly between brain tumors. In GBM, CD4^+^ T cells preferentially differentiate into the T helper cell 17 (Th17) subset [234]. Although CD4^+^ T cells are present in gliomas, they often skew towards a regulatory phenotype rather than proinflammatory helper subsets. FOXP3^+^ Tregs are enriched in the BrM microenvironment but are less prevalent in gliomas [211], [235], potentially due to tumor-associated macrophage-mediated T cell reprogramming through mechanisms such as tryptophan metabolism [235]. Tregs suppress effective anti-tumor immunity by secreting immunosuppressive cytokines like IL-10 and TGF-β and by inhibiting the activation and proliferation of other T cell subsets [236]. In addition, vaccine-induced antigen-specific follicular helper T cell-like CXCL13^+^CD4^+^ T cells have been detected in inflammatory glioma lesions following vaccination [230].

Similarly, CD8^+^ T cells’ functional states vary markedly between brain tumors [237]. Within the “non-supportive” microenvironment of gliomas, CD8^+^ T cells are frequently immature or insufficiently activated, exhibiting tissue-repair signatures, unexpanded T cell receptor (TCR) repertoires associated with anti-inflammatory signaling, elevated recognition of self-epitopes, and anti-proliferative stress or pro-death programs [238]. In GBM, CD8^+^ T cells are typically functionally exhausted [234]. Although the infiltrating CD8^+^ T cell population is dominated by clonally expanded granzyme K (GZMK)^+^ effector subsets [239], these cells display high PD-1 expression with relatively low levels of LAG-3 and T cell immunoglobulin and mucin domain-containing protein 3 (TIM-3) [240], [241]. This PD-1^+^ population corresponds to terminally exhausted T cells with an eomesodermin (EOMES)^high^, T-box expressed in T cells (T-BET)^low^ phenotype, which correlates negatively with proliferation [240]. In “supportive” microenvironments, such as melanoma and lung cancer, CD8^+^ T cells more often display a tumor-reactive exhausted phenotype, characterized by expanded TCR repertoires and immunopeptidomes enriched for conserved cancer antigens or neoantigens [238]. Consistently, in BrMs, the PD-1/PD-L1 pathway is widely activated, most CD8^+^ TILs express PD-1, and T cell clonotypes show spatial heterogeneity [231]. These features help explain the limited efficacy of immune checkpoint blockade in brain tumors, despite it not typically being classified as an immunologically “cold” tumor.

In recent years, advances in transcriptomics have confirmed the association between the proportion of memory T cells in brain tumors and clinical outcomes. Most CD8^+^ T cells are memory T cells, with comparable proportions in BrMs and gliomas [242]. In GBM, a higher frequency of central memory CD4^+^ and CD8^+^ T cells is positively correlated with OS [242], suggesting that memory T cell populations may contribute to more favourable clinical outcomes. These memory T cells are typically localized within hypoxic and vascular niches [243] **(**Fig. 6**a)**.

Despite their presence, T cell function is severely constrained by immunosuppressive factors within the BTME [244]. Canonical immune checkpoint pathways, including PD-1/PD-L1, CTLA-4, TIM-3, LAG-3, and TIGIT, cooperatively impair the effector functions of both CD8^+^ and CD4^+^ T cells by inhibiting TCR signaling, reducing cytotoxic mediator secretion, and stabilizing exhausted states [245], [246]. Targeting these pathways has also demonstrated anti-tumor efficacy in clinical studies of brain tumors [247], [248], [249], [250], [251], [252], [253]. Beyond classical checkpoints, B7 family members further suppress T cell activity in GBM. B7-H4 induces early functional dysfunction in CD8^+^ TILs by inhibiting the phosphorylation of Akt and endothelial nitric oxide synthase (eNOS) [254], whereas B7-H6 mediates secondary lysis of activated T cells by NK cells via NKp30-dependent recognition [255]. Immunosuppressive intracranial milieu may also limit the efficacy of immunotherapy, as high-dose radiotherapy combined with anti-PD-1 therapy shows extracranial but not intracranial activity in breast cancer BrMs, largely due to suppression by intracranial neutrophils and Trem2^+^ macrophages. Furthermore, systemic immunosuppression further contributes to bone marrow T cell retention via the chemokine receptor 8 (CCR8)/chemokine (C-C motif) ligand (CCL)1 /CCL8 axis [256].

Therapeutic strategies targeting T cell regulation have advanced substantially. These include the CNS-targeting chimeric antigen receptor (CAR)-T cell therapy with EphA3 demonstrating robust anti-tumor activity and immune memory formation [257]; an “open-source threshold” strategy combining CXCL10 and IL-2 to enhance recruitment and relieve exhaustion [258]; and low-frequency ultrasound-guided delivery techniques to enhance T cell infiltration [258]. CD8^+^ T cells usually function via MHC-Ⅰ recognition. However, Lerner *et al*. [259] confirmed CD8^+^ T cells persistently eliminate MHC-Ⅰ-negative tumor cells through the NK group 2 member D (NKG2D)-NKG2D ligand (NKG2DL) axis.

In summary, T cell phenotypes in brain tumors exhibit significant heterogeneity **(**Fig. 6**a)**. The central challenge lies in overcoming the brain tumor-specific immunosuppressive BTME and establishing comprehensive immunotherapeutic strategies that coordinately activate both T cells and myeloid compartments. Promoting T cell recruitment and activation by modulating chemokine and cytokine pathways (e.g., CXCL9/10-IL-2) [258], combined with metabolic remodeling and exhaustion reversal, may improve durability and efficacy. CAR-T cells targeting EphA2, B7-H3, and GD2 have shown potent anti-tumor activity and immune memory formation in preclinical models [257], [260], [261], [262]. Further optimization of delivery strategies and local immune modulation, combined with spatial multi-omics-guided analysis of cellular interaction networks, will be essential for achieving durable and precise immune responses to brain tumors.

#### B cells

B cells are key components of the adaptive immune system, responsible for antibody production, antigen presentation, and immune regulation. In the BTME, B cells exhibit functional heterogeneity. They can exert anti-tumor effects by forming immune synapses and activating stem-like CD8^+^ T cells, but specific B-cell subpopulations may also promote immune suppression. The functional plasticity highlights B cells as a potential therapeutic target in brain tumor immunotherapy [263], [264].

Biermann *et al*. [265] sequenced 22 treatment-naive melanoma BrMs samples and reported that lymphoid aggregates in melanoma BrM were enriched with differentiated tumor-infiltrating B cells. These lesions also contained a high proportion of MDMs and dysfunctional thymocyte selection associated high mobility group box (TOX)^+^CD8^+^ T cells. In GBM, B cells and T cells form clusters with a radius of 15 μm, indicating the formation of immune synapses. Activation of B cells with a CD40 agonist together with IFN-γ and B-cell activating factor (BAFF) generates a highly effective antigen-presenting B-cell population termed BVax. BVax promotes the expansion of T-cell factor 1 (TCF-1)^+^ PD-1^-^ stem-like CD8^+^ T cells in GBM. Compared with DC-activated CD8^+^ T cells, BVax-activated CD8^+^ T cells showed enhanced functional reinvigoration, characterized by increased TCF-1 and increased granzyme B expression [266].

#### Natural killer cells

Natural killer (NK) cells are central components of the lymphoid lineage and play a critical role in antitumor immunity. Unlike T cells, NK cells can be rapidly activated without antigen-specific priming and exert cytotoxic effects through the release of proinflammatory cytokines like TNF and IFN-γ and cytotoxic molecules such as perforin and granzymes [267]. Through these mechanisms, NK cells cooperate with CTLs and APCs to promote anti-tumor immune responses [268], [269]. NK cell activation is tightly regulated by the balance between activating and inhibitory surface receptors [270]. When target cells express activating ligands such as MHC-Ⅰ-related molecules or lack inhibitory signals, NK cells can rapidly initiate cytolytic responses [269], [271], [272] **(**Fig. 6**b)**.

Within the immune landscape of brain tumors, NK cells constitute approximately 2.5% of leukocytes and display marked phenotypic heterogeneity [44], [273]. IDH1-wild-type gliomas are enriched with immature, immunosuppressive CD16^-^ NK cells, whereas *IDH1*-mutant gliomas and BrMs contain more cytotoxic CD16^+^ NK cells [44]. Although less frequent than T cells, NK cells profoundly influence the abundance of CD8^+^ TILs through the coordination of response molecule expression and T cell migration mediators [274], [275]. Notably, activated T cells expressing B7-H6 can become targets of NK cell cytotoxicity via NKp30 engagement, suggesting an NK cell-dependent checkpoint mechanism that may limit T cell activity and affect immunotherapy efficacy [255].

In the BTME, NK cell function is strongly constrained by multiple immunosuppressive mechanisms. Hypoxia inhibits NK cell cytotoxicity through hypoxia-inducible factor 1α (HIF-1α) signaling [276], and immune checkpoint molecules such as cytokine inducible SH2 containing protein (CISH) and TIM-3 negatively regulate NK cell activity [277], [278]. Engagement of TIM-3 leads to direct functional impairment of NK cells [279]. TGF-β serves as a central suppressive factor by inhibiting NK cell-GSC interactions through integrin αv-mediated contact and by impairing NK cell metabolism via suppression of mTOR signaling and induction of galectin production. TGF-β is abundantly secreted by astrocytes and myeloid cells within the BTME, further reinforcing NK cell dysfunction [280].

In response to NK cell pressure, brain tumor cells develop multiple immune evasion strategies. These include the downregulation or shedding of activating ligands such as NKG2DL, often driven by TGF-β signaling [281], [282]. Circular enhancer of zeste homolog 2 (CircEZH2) also contributes to immune escape by suppressing NKG2D ligands [283]. GBM cells further evade NK cell recognition by overexpressing inhibitory MHC-Ⅰ molecules such as human leukocyte antigen (HLA)-E and HLA-G [284]. Importantly, the susceptibility of different subtypes of GSCs to NK cell-mediated killing varies, with mesenchymal GSCs being preferentially sensitive to NK cell cytotoxicity [285].

Advances in genetic engineering have significantly improved the therapeutic efficacy of NK cells [286]. Key developments include CRISPR-Cas9-mediated deletion of inhibitory regulators such as CISH, TIM-3, and HIF-1α [276], [277], [278], as well as the generation of CAR-NK cells targeting human epidermal growth factor receptor 2 (HER2) and CD19, which have demonstrated favourable safety profiles [287], [288]; GD2-CAR NK-92 cells show specific activity against CSCs [289]; and the HEFDS-NK system enabled NK cell traversal across the BBB [290]. Additionally, NK group 2 C (NKG2C)^+^ NK cells [291] and killer cell lectin like receptor B1 (KLRB1)^+^ T cell subsets [292] have shown distinct therapeutic value, and IL-21 enhances NK cell function through CEBPD-driven epigenetic reprogramming [293], [294].

Combination strategies further expand the therapeutic scope of NK cell-based therapies [289]. The progesterone/abiraterone therapy promotes NK cell infiltration into tumors [295], while aurora kinase A (AURKA) inhibitors enhance NK cell cytotoxicity by modulating PD-L1 and MHC-Ⅰ expression [296]. PD-1 blockade shows increased efficacy in tumors with high NKG2C expression [297]. Notably, combining CAR-T cells targeting the poliovirus receptor with NK-92 cells produces a synergistic suppression of tumor recurrence [298]. Clinically, phase Ⅰ trials have confirmed the safety and feasibility of HER2-CAR-NK cells [288]. The remaining challenges include optimizing *in vivo* persistence, overcoming metabolic constraints, and developing off-the-shelf NK cell products, indicating NK cell-based therapy as a promising and evolving modality in brain tumor treatment.

#### Neutrophils

Lad *et al*. [299] discovered “hybrid” polarized neutrophils with dendritic features in both GBM patients and murine models. These immature neutrophils, which do not originate from circulating populations, are instead derived from local cranial bone marrow. The cranial marrow microenvironment plays a crucial role in the development of APCs, including tumor-associated neutrophils (TANs), which can induce T cell cytotoxicity and promote immune memory formation [299].

### Tertiary lymphoid structures

Tertiary lymphoid structures (TLSs) are highly organized lymphoid tissues that form in response to chronic inflammation or within the BTME **(**Fig. 6**c)**. They resemble secondary lymph nodes with distinct compartments, such as B and T cell zones [300], [301], [302]. TLSs promote anti-tumor immunity by recruiting immune cells, such as CTLs and memory B cells [302]. Their presence is consistently associated with improved prognosis and better responses to immunotherapy, positioning them as potential biomarkers or novel therapeutic targets in cancer treatment [302].

A recent spatial transcriptomic study has categorized TLSs in gliomas into three distinct subtypes with distinct immune compositions and maturation statuses: 1) T-low-TLS, characterized by low T cell abundance and enrichment of neuronal-like and stem cell-like tumor cell genealogies, suggesting an early developmental stage of TLS; 2) B:T-TLS, defined by B-cell and T-cell interactions, high expression of antigen presentation-related genes [CD74, HLA-B, and major histocompatibility complex, class II, DR alpha (HLA-DRA)], and co-localization of LAMP3^+^ DCs and CD8^+^ cytotoxic T cells, indicating features of mature TLSs; 3) PC-TLS, enriched with plasma cells expressing immunoglobulin genes and plasma cell markers, suggesting local antibody production capability [303]. In B:T-TLS and PC-TLS, B cells and T cells are more clearly spatially separated, whereas they are intermingled in T-low-TLS. This compartmentalization reflects TLS’s functional maturity and is closely associated with local antigen presentation and humoral immune activation [299]. The co-localization of LAMP3^+^ DCs with CD8^+^ cytotoxic T cells in B:T-TLS further supports the functional characteristics of mature TLSs [303] **(**Fig. 6**c)**.

A further analysis in glioma models revealed that TLSs are composed of dense clusters of CD45^+^ immune cells, with central enrichment of CD19^+^ B cells and inclusion of CD4^+^ and CD8^+^ T cells, as well as CD11b^+^ myeloid cells [304]. Active immune responses are indicated by the presence of PD-1^+^ follicular helper T cells, CD19^+^CD38^+^ memory B cells, CD31^+^ mouse endothelial cell antigen 79 (MECA-79)^+^ high endothelial venules (HEVs), and Ki-67^+^ proliferating B cells. Although typical CD21/35^+^ follicular DCs are absent, the overall architecture suggests the presence of mature TLSs [304].

The formation of TLSs is regulated by multiple molecular mechanisms. The adeno-associated viral vector to express LIGHT in the glioma vasculature (AAV-LIGHT) induces tumor-associated HEVs and T cell-rich TLSs [305]. Additionally, radiotherapy-derived exosomes promote TLS formation via the cGAS-STING signaling pathway [306]. The maturation of TLSs in gliomas is strongly influenced by the local microenvironment. SEC61G expression is negatively correlated with TLSs maturation [51]. T-low-TLS typically forms in regions with low immune activity, severe hypoxia, or necrosis, where VEGF signaling, glycolysis, and HIF-1α-mediated metabolic reprogramming are suppressed. This suppression hinders B-cell differentiation and plasma cell formation [303]. In contrast, in regions with heightened immune activation and increased DC, B-cell and T-cell activity, TLSs undergo progressive maturation along the B:T-TLS to PC-TLS differentiation trajectory [303] **(**Fig. 6**c)**.

In breast cancer patients with BrMs, high TLS density is closely associated with significantly prolonged OS and PFS [307], [308]. Similarly, in lung cancer BrMs, high TLS density, but not high TIL density, correlates with improved postoperative survival [309]. Notably, TLSs distribution varies by organ, with higher frequencies in lung and liver metastases compared to BrMs [310]. However, TLS’s relationship with immunotherapy remains poorly understood [304], [309], [310].

In brain tumors, multiple therapeutic strategies have demonstrated the capacity to induce TLS. TLR agonists promote TLS formation and enhance anti-tumor immunity by activating CD4^+^ and CD8^+^ T lymphocytes in glioma [304]. Human cytomegalovirus (HCMV) vaccine treatment increases both the number of TLSs and T lymphocyte infiltration [311]. However, the anti-CD40 antibody (αCD40) therapy, inducing TLSs formation, also recruits immunosuppressive CD11b^+^ B cells, which can diminish the effectiveness of checkpoint blockade [312]. These findings reveal diverse regulatory mechanisms of TLSs and suggest potential strategies for targeting TLSs in the BTME **(**Fig. 6**c)**.

Extracranial tumor studies have highlighted TLS’s role in immune activation and anti-tumor response. For example, IL-33 has been implicated in TLS-associated immune regulation in pancreatic cancer CXCL13^+^CD160^+^CD8^+^ T cells have been linked to TLS’s function in gastric cancer, and local antibody production has been observed in renal cancer [313], [314], [315]. These insights suggest that enhancing TLSs could improve brain tumor therapy. Future research should focus on elucidating the dynamic regulatory mechanisms of TLSs formation in the BTME, developing standardized evaluation methods, and exploring strategies to enhance or specifically target TLSs [316] **(**Fig. 6**c)**.

### Vascular system

In brain tumors, the vascular system shows abnormalities, such as increased density, enhanced permeability, and disrupted blood flow [317], [318]. Abnormal blood vessel formation involves endothelial cell-dependent angiogenesis and tumor cell-dependent vascular mimicry (VM) [319]. An endothelial transcriptome analysis revealed the dysregulation of genes related to cell proliferation, angiogenesis, ECM deposition, and BBB dysfunction in the vascular systems of GBM and BrMs [320]. BBB disruption in BrMs is spatially heterogeneous, with increased permeability in the tumor core, whereas the tumor edge often remains relatively intact [321].

**Fig. 7:**
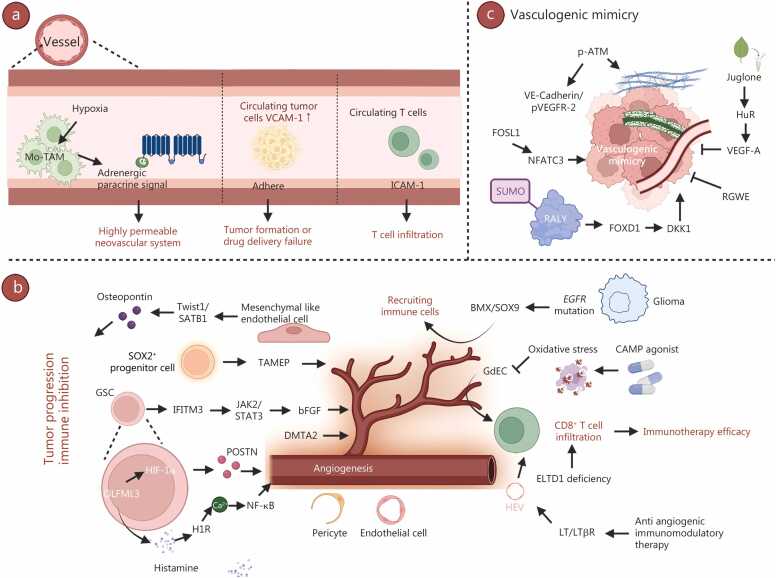
**Vascular remodelling and vascular mimicry in brain tumors: mechanisms of angiogenesis, immune modulation, and therapeutic implications. a, b** Angiogenic remodelling in brain tumors and its impact on immune escape and therapy. **a** Mo-TAMs are located in the perinecrotic, hypoxic niche and acquire hypoxic response characteristics. Through adrenergic paracrine signalling, Mo-TAMs disrupt endothelial adhesion junctions, thereby stimulating a hyperpermeable neovascular system and hindering drug delivery in GBM xenografts. VCAM-1 on activated cerebral endothelial cells enhances the adhesion of circulating tumor cells, facilitating metastatic seeding in the brain. ICAM-1 positivity in PVV-like vessels is associated with T cell infiltration in tumors. **b (Left side)** A distinct population of mesenchymal-like endothelial cells establishes an immunosuppressive vascular niche in GBM. Through Twist1/SATB1-mediated sequential transcriptional activation, tumor endothelial cells secrete osteopontin, which promotes immunosuppressive macrophage phenotypes. TAMEP derived from SOX2-positive progenitor cells resident in the CNS promote GBM growth by controlling tumor-associated angiogenesis. GSC-derived IFITM3 activates JAK2/STAT3 signalling and induces secretion of bFGF, ultimately enhancing angiogenesis. CLOCK-guided expression of OLFML3 upregulates POSTN via HIF-1α, and secreted POSTN promotes tumor angiogenesis by activating TBK1 signalling in endothelial cells. Histamine secreted by GSCs activates endothelial cells via the histamine H1R-Ca^2+^-NF-κB axis, thereby promoting angiogenesis and GBM progression. **(Right side)***EGFR*-mutant glioma cells can adopt pericyte functions in a BMX/SOX9-dependent manner, stabilizing vascular structures and recruiting immune cells. cAMP activators induce the differentiation of GdECs by inducing oxidative stress, normalizing the tumor vasculature, and increasing CD8^+^ effector T cells. ELTD1 deficiency reduces vascular abnormalities and improves T cell recruitment after PD‑1 blockade in glioma. Anti-angiogenic immunoregulatory therapy induces the differentiation of postcapillary venules into HEVs through LT/LTβR signaling. Tumor-associated HEVs lymphocyte entry and provide a niche supporting PD-1^-^ and PD-1^+^TCF1^+^CD8^+^ T progenitor cells and exert anti-tumor effects. **c** Mechanisms and therapeutic targeting of VM in brain tumors. p-ATM promotes VM by remodelling the ECM and activating VE‑cadherin/pVEGFR‑2. FOSL1 interacts with NFATC3, enhancing its DNA-binding capacity and driving VM-associated transcriptional programs. SUMOylation of RALY increases its stability and elevates FOXD1 expression, which in turn activates the DKK1 promoter and promotes glioma cell migration, invasion, and VM formation. Juglone inhibits VM in glioma by suppressing HuR-mediated VEGF-A expression. RGWE induced the death of GBM patient-derived CSCs and impaired VM. Mo-TAMs. Monocyte-derived tumor-associated macrophages; GBM. Glioblastoma; VCAM-1. Vascular cell adhesion protein 1; ICAM-1. Intercellular adhesion molecule 1; PVV. Perivenular venules; Twist1. Twist family bHLH transcription factor 1; SATB1. Special AT-rich sequence binding protein 1; TAMEP. Tumor-associated cells with a myeloid-like expression profile; SOX. SRY-box transcription factor; CNS. Central nervous system; GSCs. Glioma stem cells; IFITM3. Interferon-induced transmembrane protein 3; JAK2. Janus kinase 2; STAT3. Signal transducer and activator of transcription 3; bFGF. Basic fibroblast growth factor; CLOCK. Circadian locomotor output cycles kaput; OLFML3. Olfactomedin-like 3; POSTN. Periostin; HIF. Hypoxia-inducible factor; TBK1. TANK-binding kinase 1; H1R. H1 receptor; NF-κB. Nuclear factor κB; EGFR. Epidermal growth factor receptor; BMX. Bone marrow tyrosine kinase on chromosome X; cAMP. Cyclic adenosine monophosphate; GdECs. Glioma-derived endothelial cells; ELTD1. EGF, latrophilin and seven transmembrane domain-containing protein 1; PD-1. Programmed cell death protein 1; HEVs. High endothelial venules; LT. Lymphotoxin; LTβR. Lymphotoxin β receptor; TCF1. T cell factor 1; VM. Vascular mimicry; pATM. Phosphorylated ATM; ECM. Extracellular matrix; VE-cadherin. Vascular endothelial cadherin; pVEGFR-2. Phosphorylated vascular endothelial growth factor receptor 2; FOSL1. FOS-like antigen 1; NFATC3. Nuclear factor of activated T cells cytoplasmic 3; FOXD1. Forkhead box D1; DKK1. Dickkopf-1; HuR. Human antigen R; VEGFA. Vascular endothelial growth factor A; RGWE. Ruta graveolens water extract; CSCs. Cancer stem cells.

Abnormal vasculature induces a hypoxic microenvironment and hinders drug penetration. Translocator protein (TSPO) deficiency in GBM induces mitochondrial dysfunction, shifting metabolism towards glycolysis, which promotes hypoxia, angiogenesis, and tumor growth [322]. Monocyte-derived TAMs (Mo-TAMs) in the necrotic perivascular niche acquire hypoxic response characteristics and disrupt endothelial adhesion junctions through adrenalin-paracrine signaling, creating a highly permeable blood vessel system that hinders drug delivery to GBM xenografts [323] **(**Fig. 7**a)**.

Abnormal tumor vasculature impacts immune responses through various mechanisms. Vascular damage reduces immune cell infiltration. A distinct population of mesenchymal-like endothelial cells creates an immunosuppressive microenvironment in GBM. Mechanistically, twist-related protein 1 (Twist1)/special AT-rich sequence-binding protein 1 (SATB1)-mediated transcriptional activation causes tumor endothelial cells to produce osteopontin, promoting an immunosuppressive macrophage phenotype [324]. A process mimicking an infarct-like microenvironment promotes BrMs, which depend on Ang-2 and VEGF [325] **(**Fig. 7**b)**.

Normalization of blood vessels may promote the infiltration of immune cells and enhance the efficacy of immunotherapy. Cyclic adenosine monophosphate (cAMP) activators impair glioma-derived endothelial cells (GdEC) differentiation, normalize tumor blood vessels, and alter tumor immune features [69], [280]. Circulating T cells preferentially extravasate into perivascular venules (PVVs), where the endothelial adhesion molecule intercellular adhesion molecule 1 (ICAM-1) is highly expressed. In BrMs, the abundance of ICAM-1^+^ PVV-like vessels correlates with TILs [326]. Moreover, epidermal growth factor, latrophilin, and seven transmembrane domain-containing protein 1 (ELTD1) deletion alleviates vascular abnormalities and enhances T cell recruitment after PD-1 blockade [327] **(**Fig. 7**b)**.

Tumor cells reside in the perivascular niche, a microenvironment closely linked to the brain’s microvascular system [328], [329]. Perivascular stromal cells in vascular niche models can direct the invasion, proliferation, and treatment response of GBM [328]. Tumor cells also directly interact with endothelial cells, inducing the expression of adhesion molecules such as ICAM-1 and vascular cell adhesion molecule 1 (VCAM-1), which facilitates tumor cell infiltration and distant metastasis [330]. VCAM-1 enhances the adhesion of circulating tumor cells to activated brain vascular endothelial cells, promoting metastatic lesion formation in the brain [330]. GSCs promote abnormal blood vessel formation, creating a “tumor-friendly” microenvironment. GSCs activate JAK2/STAT3 signaling via IFITM3, leading to basic fibroblast growth factor (bFGF) secretion [331], and the circadian locomotor output cycles kaput (CLOCK)-olfactomedin-like 3 (OLFML3)-HIF-1α-periostin (POSTN)-TANK-binding kinase 1 (TBK1) signaling axis drives angiogenesis [332]. Additionally, SRY-box transcription factor 2 (SOX2)-positive progenitor-derived tumor-associated cells with a myeloid-like expression profile (TAMEP) contributes to angiogenesis [333], while histamine secreted by GSCs activates endothelial cells via the H1R-Ca^2+^-NF-κB axis [334], further enhancing angiogenesis. *DMTA2* knockdown in glioma spheroids affects the stability of GSC-dependent tube formation *in vitro* [335] **(**Fig. 7**b)**.

VEGF-C-mediated lymphatic drainage improves anti-tumor efficacy. When confined to the CNS, CD8^+^ T cell-mediated immunity is limited, causing tumor growth. Impaired meningeal lymphatics disrupt the trafficking of tumor antigen-bearing DCs and reduce CD8^+^ T cell activation after radiotherapy. By contrast, VEGF-C overexpression promotes meningeal lymphatic expansion, which enhances CCL21-dependent DC trafficking and subsequently improves CD8^+^ T cell priming, thereby increasing sensitivity to radiotherapy. Consistently, exogenous VEGF-C promotes CD8^+^ T cell activation in deep cervical lymph nodes, leading to T cell migration to the tumor, GBM cells clearance, and durable anti-tumor memory response [25], [336]. These findings suggest that therapeutic modulation of the meningeal lymphatic system may enhance anti-tumor immunity in brain tumors [337] **(**Fig. 7**b)**.

Changes in endothelial cells within the BTME not only affect angiogenesis but also disrupt the BBB, a critical structure that protects the CNS. Impaired BBB function is a hallmark of a brain tumor. The IL-6-STAT3 signaling downregulates tight junction proteins and ATP-binding cassette (ABC) transporters in the BBB [338], while STAT overactivation causes abnormal permeability [339], [340]. Mo-TAMs disrupt endothelial junctions through adrenaline signaling [323], and breast cancer cells induce morphological and functional changes in brain endothelial cells through EVs [341].

Metastatic tumor cells actively remodel the BBB to facilitate brain colonization through both direct interactions with brain endothelial cells and systemic tumor-derived factors. For example, the interaction between breast cancer cells and brain microvascular endothelial cells compromises BBB integrity by inducing both paracellular and transcellular hyperpermeability, while also enhancing the invasive and migratory properties of tumor cells [342]. In addition, systemic secretion of ectonucleotide pyrophosphatase/phosphodiesterase 1 (ENPP1) from primary breast tumors induces endothelial dysfunction by impairing insulin signaling and the Akt/GSK3β/β-catenin pathway [343]. EVs derived from breast cancer cells induce morphological, motility, and proteomic changes in brain endothelial cells via transcytosis, enabling tumor cells to breach the BBB and spread to the brain [341]. Consistently, although brain-seeking cell subline and parental breast cancer cells behave similarly in terms of adhesion, the brain-seeking subline has a stronger impact on the brain endothelium integrity and increased migratory ability [344].

Tumor cells undergo VM, where they form tubular structures that function as substitutes for blood vessels [345]. Key molecules involved in VM include pATM, which activates VE-cadherin/pVEGFR2 [346], the FOSL1-nuclear factor of activated T cells cytoplasmic 3 (NFATC3) complex [347], and the SUMOylation of RALY heterogeneous nuclear ribonucleoprotein (RALY-SUMOylation)-forkhead box D1 (FOXD1)-dickkopf 1 (DKK1) signaling axis [348]. VM functions as a channel resembling a blood vessel, supplying essential nutrients for tumor growth and significantly contributing to resistance in GBM [349]. Juglone inhibits VM by suppressing the HuR-VEGF-A pathway [350], while RGWE components can disrupt VM at sublethal doses [351] **(**Fig. 7**c)**.

Targeting the BBB and tumor vasculature represents a promising therapeutic strategy for brain tumors. Targeting the vascular GP130 signaling pathway enhances cancer cell sensitivity to TMZ by attenuating blood-tumor barrier formation and improving drug penetration [352]; cAMP interferes with glioma-derived endothelial cell differentiation, normalizes tumor vasculature， and alters tumor immune features [69], [353]. Ectopic expression of VEGF-C, which promotes CCL21-dependent DC trafficking and CD8^+^ T cell activation, leads to rapid clearance of the glioblastoma [25], [336]. Additionally, peripheral targeting of the meningeal lymphatic system [337] and physical BBB opening techniques also show potential effects [321], [354], [355]. Future challenges include the precise regulation of vascular heterogeneity, the synergistic blockade of VM and classic angiogenesis, the dynamic balance between BBB disruption and reconstruction, and the timing of vascular targeting with immunotherapy combinations. These strategies need to be optimized based on patient-specific vascular characteristics.

### Extracellular matrix

Extracellular matrix (ECM) remodeling is a key feature of the BTME, influencing tumor progression and therapy resistance through altering its structural and biochemical properties [356]. Key ECM components, such as collagen, fibronectin, and hyaluronic acid, undergo significant crosslinking and stiffening in tumors [357], [358]. ECM degradation, regulated mainly by the MMP family, is crucial for tumor invasion. The overexpression of MMP-2 and MMP-9 promotes ECM degradation, releasing growth factors and enabling tumor cell migration [359]. Notably, the stiffness difference between the vascular and parenchymal interfaces directs the infiltration of tumor cells [360], indicating that ECM physical properties influence tumor distribution. Streitberger *et al*. [361] demonstrated that ECM fluidity plays a critical role in tumor invasiveness. These mechanical changes activate mechanosensitive channels like Piezo1/Piezo2 and the yes-associated protein 1 (YAP1)/ transcriptional coactivator with PDZ-binding motif (TAZ) signaling pathway, aiding tumor migration and invasion [362], [363].

**Fig. 8:**
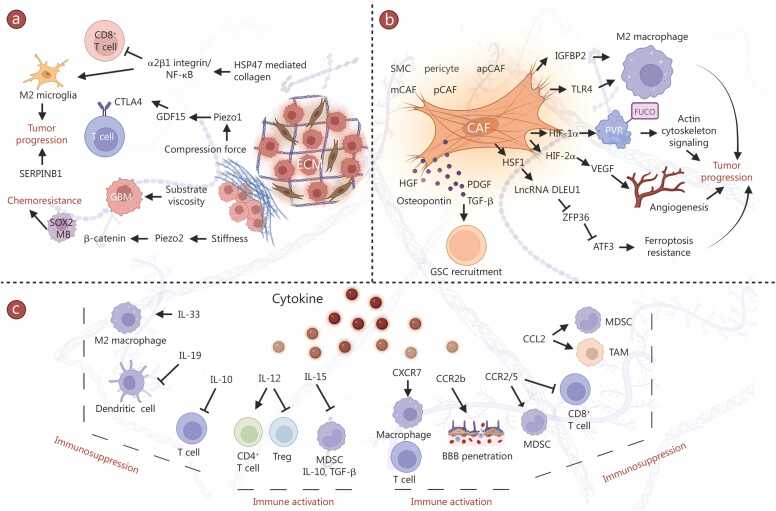
**ECM, CAFs, and cytokine-mediated immune modulation. a** Effects of ECM physical properties on tumor growth and immune escape. During tumor progression, alterations in ECM stiffness, viscosity, and compressive forces profoundly influence tumor behaviour and therapy response. Compression promotes glioma progression by stimulating Piezo1 and increasing GDF15 and CTLA4. Increased substrate viscosity weakens the inhibitory effects of TMZ on GBM. In medulloblastoma, SOX2^+^ tumor cells sense matrix stiffness through Piezo2, stabilize β-catenin and drive chemoresistance. SERPINB1 upregulated in BrMs may be involved in metastatic tropism. HSP47-mediated collagen deposition promotes microglial polarization to the M2 phenotype via the α2β1 integrin/NF-κB pathway, leading to suppression of CD8⁺ T cell anti-tumor activity and facilitation of BrM progression. **b** CAF heterogeneity and protumoral mechanisms. In breast cancer, there are five subpopulations of CAFs: SMCs, pericytes, apCAFs, pCAFs, and mCAFs. pCAFs are the predominant CAF subtype in breast cancer and are associated with lymph node, bone, and BrM. PDGF and TGF‑β have been identified as mediators of GSC influence on CAFs. Osteopontin and HGF have been identified as mediators of CAF-induced GSC enrichment. Moreover, CAFs induce M2 macrophage polarization by regulating TLR4 and IGFBP2. Under hypoxic conditions, HIF1α fucosylates PVR and triggers its secretion from BrM-associated CAFs, which promotes tumor progression. In lung cancer BrMs, HIF-2α activation in CAFs enhances angiogenesis via VEGF signalling, promotes metabolic reprogramming, and accelerates tumor growth. The oncogenic lncRNA DLEU1 confers ferroptosis resistance in GBM cells by binding ZFP36 and epigenetically repressing ATF3. **c** Cytokine-mediated immune modulation in the BTME. IL-33 induces regulatory chemokines that collectively recruit M2 macrophages and establish a protumor environment. IL-19 acts as a predictive immunosuppressive cytokine in the tumor periphery that significantly reduces DCs and monocyte/macrophage cells. IL-10, released by HMOX1^+^ myeloid cells, located in the mesenchymal-like tumor region, drove T cell exhaustion. IL-12 enhances CAR‑T cell cytotoxicity and remodels BTME by increasing proinflammatory CD4^+^ T cells, reducing Tregs, and activating myeloid cells. IL-15-modified CAR-T cells inhibit MDSCs and reduce immunosuppressive factors (IL-10 and TGF-β), making it a dual-target agent for GBM cells and MDSCs. Chemokine signalling further regulates immune cell trafficking. The CXCR7 agonist VUF11207 enhanced tumor cytotoxicity and reversed microglia-mediated immune suppression. CCR2b co-expression in B7‑H3 CAR-T cells improve BBB penetration and anti-tumor efficacy. The co-inhibition of the chemokine receptors CCR2 and CCR5 synergizes with anti‑PD‑1 therapy, selectively reducing monocyte-derived MDSCs while increasing CD8^+^ T cell infiltration and cytokine production. H19-IRP, which promotes immune suppression by activating CCL2 and galectin-9 transcription, recruiting MDSCs and TAMs and driving T cell exhaustion in GBM. ECM. Extracellular matrix; CAFs. Cancer-associated fibroblasts; GDF15. Growth differentiation factor 15; CTLA4. Cytotoxic T-lymphocyte-associated protein 4; TMZ. Temozolomide; GBM. Glioblastoma; SOX2. SRY-box transcription factor 2; SERPINB1. Serpin family B member 1; BrMs. Brain metastases; HSP47. Heat shock protein 47; NF-κB. Nuclear factor κB; SMCs. Smooth muscle cells; apCAFs. Antigen-presenting CAFs; pCAFs. Proliferative CAFs; mCAFs. Matrix CAFs; PDGF. Platelet-derived growth factor; TGF-β. Transforming growth factor-β; GSC. Glioma stem cell; HGF. Hepatocyte growth factor; TLR4. Toll-like receptor 4; IGFBP2. Insulin-like growth factor-binding protein 2; HIF. Hypoxia-inducible factor; PVR. Polio virus receptor; VEGF. Vascular endothelial growth factor; DLEU1. Deleted in lymphocytic leukemia 1; ZFP36. Zinc finger protein 36; ATF3. Activating transcription factor 3; BTME. Brain tumor microenvironment; IL. Interleukin; DCs. Dendritic cells; HMOX1. Heme oxygenase 1; CAR-T. Chimeric antigen receptor T; Tregs. Regulatory T cells; MDSCs. Myeloid-derived suppressor cells; CXCR7. C-X-C chemokine receptor type 7; CCR. C-C chemokine receptor; B7-H3. B7 homolog 3; BBB. Blood-brain barrier; anti-PD-1. Anti-programmed cell death protein 1; CCL2. C-C motif chemokine ligand 2; TAMs. Tumor-associated macrophages.

ECM properties contribute to therapeutic resistance in tumors **(**Fig. 8**a)**. Increased ECM stiffness and viscosity reduce chemotherapy sensitivity [364]. In medulloblastoma, SOX2^+^ tumor cells rely on mechanosensitive ion channel Piezo2 to sense substrate stiffness, which maintains calcium levels, myosin tension and adhesion. This promotes β-catenin and leads to chemotherapy resistance [363]. In breast cancer BrMs, cancer-stromal interactions promote mucin and glycoprotein production, which reduces sensitivity to neratinib [365]. These findings indicate that ECM physical properties contribute to therapy resistance.

ECM remodeling also regulates the BTME, forming a complex network. HSP47-mediated collagen deposition promotes microglial polarization towards the M2 phenotype via the α2β1 integrin/NF-κB signaling axis, suppressing CD8^+^ T cells and promoting BrM [207]. Specific ECM components, such as SERPINB1, can influence organ-specific metastasis [366]. These findings highlight ECM’s role in shaping the immunosuppressive microenvironment.

Therapeutic interventions targeting ECM have shown potential treatment promise [359]. Future research should focus on the ECM heterogeneity and combination therapies targeting both the physical properties and biochemical composition [367], [368]. A deeper understanding of the interactions between ECM remodeling and the immune microenvironment will provide new theoretical foundations and therapeutic targets to overcome the current challenge of treatment resistance [369], [370] **(**Fig. 8**a)**.

### Cancer-associated fibroblasts

Cancer-associated fibroblasts (CAFs) are key regulators in the BTME and display functional diversity across tumor types [371], [372], [373] **(**Fig. 8**b)**. For example, single-cell sequencing revealed site-specific CAF heterogeneity; inflammatory CAFs (iCAFs) predominate in NSCLC BrMs and promote metastasis by activating MET and other invasion-related molecules [374]. In breast cancer, five CAF subpopulations have been identified: smooth muscle cells (SMCs), pericytes, antigen-presenting CAFs (apCAFs), proliferative CAFs (pCAFs), and matrix CAFs (mCAFs), with pCAFs predominating in cases with lymph node, bone, and BrMs [375]. Consistently, BrM-associated CAFs show high mRNA expression of type Ⅰ collagen [376], and in breast cancer BrMs, elevated expression of PDGFR-β, α-SMA, and type Ⅰ collagen correlates with tumor size and recurrence [375], [377], [378]. CAFs in BrMs may originate from perivascular pericytes, endothelial progenitor cells, or transformed astrocytes [377]. Furthermore, GBM, once considered devoid of fibroblasts, also contains CAF-like cell populations [379].

CAFs promote tumor progression and shape the immune microenvironment through multiple mechanisms [374] **(**Fig. 8**b)**. CAF-associated insulin-like growth factor-binding protein 2 (IGFBP2) drives glioma progression by inducing M2 macrophage polarization [380]. GSCs recruit CAFs through PDGF and TGF-β signaling, and in turn, CAFs enhance tumor stemness by secreting osteopontin and HGF [379]. CAF-derived extra domain A (EDA) fibronectin also contributes to immune suppression by inducing M2 macrophage polarization and promoting GBM [379]. In BrMs, hypoxic conditions further enhance the pro-tumor functions of CAF. For example, BrM-associated fibroblasts secrete HIF-1α-mediated polio virus receptor (PVR) fucosylation to promote breast cancer invasion [381], whereas in lung cancer BrMs, CAFs exert HIF-2α-mediated proangiogenic effects [371]. CAFs also suppress anti-tumor immunity by upregulating PD-L1, promoting Tregs infiltration, and secreting collagen that engages the leukocyte associated immunoglobulin like receptor 1 (LAIR1) receptor to enhance immune suppression [372], [382], [383]. In lung cancer BrMs, CAFs promote angiogenesis and metabolic reprogramming through the VEGF pathway, thereby accelerating tumor growth [371], [372]. These findings underscore the central role of CAFs in tumor progression and immune suppression. Future research needs to further delineate the function of specific CAF subpopulations and develop precision strategies to overcome CAF-mediated resistance.

### Cytokines

The cytokine network in the BTME constitutes a highly complex regulatory system that governs tumor progression and treatment response [384], [385]. TGF-β is a central regulator with a well-recognized biphasic role. During early tumor development, TGF-β acts as a tumor suppressor [386], whereas at later stages, it promotes tumor invasion and metastasis by facilitating microtubule formation [387] and inducing immune suppression [388], [389], [390]. This temporally functional switch suggests the need for precise timing when therapeutically targeting the TGF-β pathway **(**Fig. 8**c)**.

ILs mediate immune cell communication and exert context-dependent effects in brain tumors [391]. Proinflammatory ILs, such as IL-12, enhance anti-tumor immunity. However, anti-inflammatory ILs, including IL-10 and IL-6, drive tumor progression by promoting proliferation, angiogenesis, and immune suppression through recruiting Tregs and MDSCs [195], [293], [392]. Mechanistically, IL-6 promotes tumor proliferation, Th17 differentiation, and metabolic reprogramming through JAK/STAT3 signaling, whereas IL-33 regulates chemokine expression through both nuclear and secretory mechanisms to recruit innate immune cells and establish a pro-tumorigenic environment [393]. In addition, IL-19 has been identified as an immune-suppressive biomarker at the tumor periphery in GBM [394], and a subset of IL-10-stimulated heme oxygenase 1 (HMOX1)^+^ myeloid cells localized to mesenchymal tumor regions drives T cell exhaustion and contributes to the immunosuppressive BTME [395]. Several ILs have been exploited to enhance immunotherapy efficacy. For example, IL-12 improves CAR-T cell cytotoxicity and remodels the BTME by increasing proinflammatory CD4^+^ T cells infiltration, reducing Treg abundance, and activating myeloid cells [195]. Similarly, IL-15 modification enables CAR-T cells to simultaneously target tumor cells and MDSCs in GBM [396] **(**Fig. 8**c)**.

Chemokines act as key spatial regulators of immune cell trafficking within tumors. The CXCL12-C‑X‑C chemokine receptor type 7 (CXCR7) axis upregulates PD-L1 expression through an NF-κB signaling [397], whereas the CCL2-CCR2b pathway enhances CAR-T cells penetration to cross the BBB [398]. Notably, CCR2 and CCR5 co-inhibition reduces myeloid cell infiltration and synergizes with PD-1 blockade [399], highlighting the therapeutic potential of multitarget strategies. In addition, the long noncoding RNA H19 contributes to the establishment of an immunosuppressive microenvironment by promoting the transcription of CCL2 and galectin-9 [400] **(**Fig. 8**c)**.

Cytokine-mediated immune regulation is closely linked to therapeutic resistance [147], [401]. Proinflammatory cytokines combine with CAR macrophages/NK cells, which significantly increase glioma treatment efficacy [402], [403]. Future studies should focus on defining the dynamic evolution of the cytokine network, developing spatiotemporally precise targeting strategies, and assessing how microenvironmental heterogeneity shapes cytokine function and therapeutic response [384], [404], [405].

### Metabolic reprogramming

**Fig. 9:**
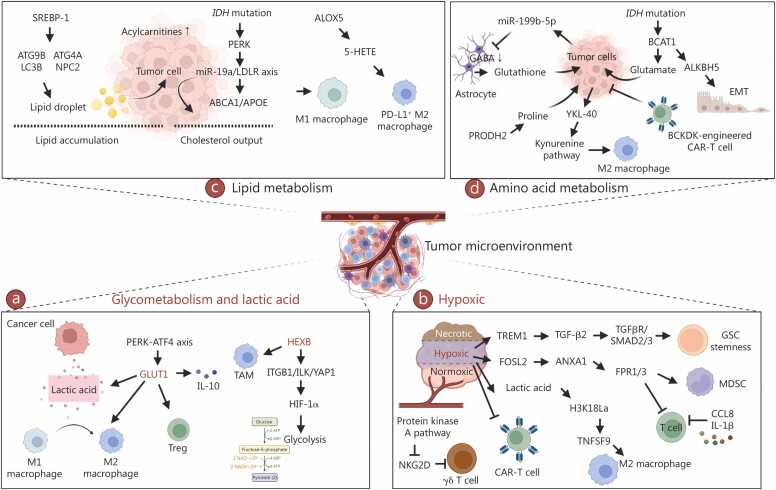
**Metabolic reprogramming in brain tumors: glucose, hypoxia, lipid and amino acid metabolism in immune modulation and tumor progression. a** Glucose metabolism. Tumor-derived signals activate the PERK-ATF4 axis in monocyte-derived macrophages, inducing GLUT1 expression and enhancing glycolysis, lactate production, and IL-10 secretion in MDMs, thereby creating an immunosuppressive microenvironment. Lactate-induced M2 polarization of TAMs promotes the invasion of pituitary adenoma cells through the secretion of CCL17. HEXB is a key regulator of glycolysis in GBM by both reprogramming TAM metabolism and intrinsically enhancing tumor glycolysis. Mechanistically, elevated HEXB stabilizes HIF-1α by activating the ITGB1/ILK/YAP1 axis. **b** Hypoxia-mediated metabolic and immune reprogramming. Hypoxia promotes tumor stemness by upregulating TREM1 and activating TGF-β2-TGFβR-SMAD2/3 signaling. Hypoxia also recruits and polarizes myeloid-derived suppressor cells (MDSCs) via the activation of the FOS-like 2, AP-1 transcription FOSL2-ANXA1-FPR1/3 axis, which facilitates tumor invasion and weakens T cell cytotoxicity. Hypoxia selectively impairs γδ T cells by inhibiting NKG2D signaling through protein kinase A activation. Accumulated glycolytic by-products, particularly lactate, are taken up by macrophages and induce M2 polarization. Mechanistically, hypoxia-driven lactate uptake increases H3K18 lactylation and TNFSF9 expression in macrophages. The hypoxic niche attracts and isolates TAMs and cytotoxic T cells, reprogramming them into an immunosuppressive state through the chemokine CCL8 and the cytokine IL-1β. CAR-T cells display impaired mitochondrial ATP production and exhibit a clear hypoxic state, reducing their cytotoxic ability. **c** Lipid metabolism. GBM cells accumulate CEs within lipid droplets. SREBP-1 upregulates key autophagy genes, including *ATG9B*, *ATG4A*, microtubule-associated protein 1 (*LC3B*), and the lysosomal cholesterol transporter (*NPC2*), promoting lipid droplet turnover and lysosomal cholesterol release to maintain membrane cholesterol homeostasis. *IDH* mutation-induced PERK activation enhances cholesterol efflux in glioma through the miR-19a/LDLR axis and upregulation of ABCA1/APOE, ultimately influencing the M1-like polarization of glioma-associated macrophages. ALOX5 and its metabolite 5‑HETE accumulate PD-L1^+^ M2‑GAMs and contribute to the immunosuppressive BTME. **d** Amino acid metabolism. Astrocyte-derived glutathione is exploited by tumor cells to sustain cysteine pools; BCAT1 is highly expressed in IDH-wild-type GBM and NSCLC BrMs, where it promotes tumor progression by generating glutamate and regulating ALKBH5-dependent EMT programs. PRODH2‑dependent proline metabolism or secretes miR‑199b‑5p to disrupt astrocytic GABA synthesis, reshaping the local metabolic niche. Furthermore, GBM‑secreted YKL‑40 activates the kynurenine pathway, reinforcing an immunosuppressive microenvironment. In addition, BCKDK engineering in CAR-T cells modifies branched-chain amino acid metabolism to enhance anti-tumor efficacy. PERK. Protein kinase R-like endoplasmic reticulum kinase; ATF4. Activating transcription factor 4; GLUT1. Glucose transporter 1; IL. Interleukin; MDMs. Monocyte-derived macrophages; TAMs. Tumor-associated macrophages; CCL. C-C motif chemokine ligand; HEXB. Hexosaminidase B; GBM. Glioblastoma; HIF-1α. Hypoxia-inducible factor-1α; ITGB1. Integrin beta 1; ILK. Integrin-linked kinase; YAP1. Yes-associated protein 1; TREM1. Triggering receptor expressed on myeloid cells 1; TGF. Transforming growth factor; SMAD2/3. SMAD family member 2/3; MDSCs. Myeloid-derived suppressor cells; FOSL2. FOS like 2; AP-1. Transcription factor subunit; ANXA1. Annexin A1; FPR. Formyl peptide receptor; NKG2D. Natural killer group 2 member D; H3K18. Histone H3 lysine 18; TNFSF9. Tumor necrosis factor ligand superfamily member 9; CAR-T. Chimeric antigen receptor T; ATP. Fulladenosine triphosphate; CEs. Cholesterol esters; SREBP. Sterol-regulatory element binding protein; ATG. Autophagy-related; LC3B. Light chain 3 beta; NPC2. NPC intracellular cholesterol transporter 2; IDH. Isocitrate dehydrogenase; LDLR. Low-density lipoprotein receptor; ABCA1. ATP-binding cassette transporter A1; APOE. Apolipoprotein E; ALOX5. Arachidonate 5-lipoxygenase; 5-HETE. 5-hydroxyeicosatetraenoic acid; M2-GAMs. M2-type glioma-associated macrophages; BTME. Brain tumor microenvironment; BCAT1. Branched-chain amino acid transaminase 1; NSCLC. Non-small cell lung cancer; BrMs. Brain metastases; ALKBH5. AlkB homolog 5; EMT. Epithelial-mesenchymal transition; PRODH2. Proline dehydrogenase 2; GABA. Gamma-aminobutyric acid; YKL-40. Chitinase-3-like protein 1; BCKDK. Branched-chain ketoacid dehydrogenase kinase.

Metabolic reprogramming is a hallmark of brain tumors and plays a central role in tumor growth, immune evasion, and therapeutic resistance [406], [407], [408]. The most prominent alteration is the Warburg effect, characterized by enhanced glycolysis despite sufficient oxygen availability **(**Fig. 9**a)**. GBM cells exhibit high glycolytic flux and produce large amounts of lactate through lactate dehydrogenase A (LDHA), while LDHB catalyzes lactate-to-pyruvate conversion to fuel mitochondrial metabolism [409], [410]. These findings suggest that GBM may exploit metabolic circuits for survival, consistent with the ANLS model [132].

Glucose metabolism is further refined by post-translational and transcriptional regulation. Zinc finger DHHC-type containing 9 (ZDHHC9)-mediated S-palmitoylation promotes glucose transporter 1 (GLUT1) localization and sustains glycolytic flux [411], while the homeobox A3 (HOXA3)-lysine demethylase 6 A (KDM6A) regulatory axis maintains aerobic glycolysis through H3K27 demethylation, accelerating GBM progression [412]. Metabolic traits may also be associated with metastasis: breast cancer BrMs display reduced glucose metabolism and immune gene expression [413], whereas lung cancer preferentially colonizes metabolically active brain regions [414].

Hypoxia is another significant characteristic of the BTME **(**Fig. 9**b)**. Single-cell analyses identify hypoxia as an adaptive strategy in IDH-wild-type GBM, where it enhances stemness via triggering receptor expressed on myeloid cells 1 (TREM1) induction and TGFβ2-TGFβR/SMAD2/3 signaling [415], [416]. Hypoxia also sustains tumor growth by activating autophagy, and acetylation-dependent activation of p21-activated kinase 1 (PAK1) promotes autophagy-related 5 (ATG5) phosphorylation and autophagic flux [417]. Beyond GBM, hypoxia also drives brain colonization by systemic malignancies, as HIF-1 activation increases the release of EVs enriched in integrin β3, thereby facilitating colonization of the brain microenvironment [418]. In parallel, hypoxia further reinforces immune evasion within the BTME. Hypoxia-induced lactate accumulation drives histone lactylation and upregulates tumor necrosis factor ligand superfamily member 9 (TNFSF9) [419], while recruitment of bone marrow-derived macrophages through the FOSL2-ANXA1-formyl peptide receptor (FPR)1/3 axis enhances tumor invasion and weakens T cell cytotoxicity [415], [420]. However, another study demonstrated hypoxia selectively impairs γδ T cells by inhibiting NKG2D signaling through protein kinase A activation [421]. In CAR-T cells, hypoxia impairs mitochondrial ATP production, thereby weakening cytotoxic capacity [422]. The hypoxic niche attracts and isolates TAMs and cytotoxic T cells, reprogramming them into an immunosuppressive state via CCL8 and IL-1β [423]. Tumor-associated myeloid cells surround the hypoxic necrotic regions of GBM and express the enzyme glycine amidinotransferase (GATM). Consequently, tumor cells absorb creatine secreted by tumor-associated myeloid cells, which protects them from hypoxic stress [424].

In addition to glucose metabolism, brain tumor also exhibits profound lipid metabolic reprogramming **(**Fig. 9**c)**. GBM cells increase fatty acid synthesis and uptake, and lipid heterogeneity correlates with poor prognosis [425]. Lipid metabolic reprogramming also has multidimensional regulatory characteristics. In glioma, cholesterol homeostasis is maintained through the sterol-regulatory element binding protein (SREBP)-1-mediated autophagy-lysosome pathway [426], while *IDH* mutations regulate cholesterol efflux via the protein kinase R-like endoplasmic reticulum kinase (PERK)-miR-19a-low-density lipoprotein receptor (LDLR) axis [427]. Moreover, lipid metabolism displays spatial heterogeneity, with distinct fatty acid metabolic patterns in tumor core versus invasive margins [428], [429]. Beyond supporting tumor growth, altered lipid metabolism also contributes to immune escape through complex cellular interactions and redox regulation. Macrophages support GBM malignancy by phagocytosing myelin from the surrounding brain tissue and transferring lipids to tumor [126], [430]. In addition, lipid metabolites can directly regulate immune checkpoints. For example, arachidonate 5-lipoxygenase (ALOX5)-derived 5-hydroxyeicosatetraenoic acid (5-HETE) activates NRF2 and promotes PD-L1^+^ M2 macrophages accumulation [431]. Conversely, lipid peroxidation can also enhance anti-tumor immunity, as TNF receptor-associated factor 3 (TRAF3)-mediated peroxidative signaling activates immune responses in GBM [432].

Altered amino acid metabolism supports growth and immune escape of brain tumor [433] **(**Fig. 9**d)**. In GBM, tumor cells show strong dependence on glutamine, reflected by elevated expression of glutaminase (GLS), and its pharmacological inhibition suppresses tumor growth [434]. This dependency is further reinforced by metabolic crosstalk with the microenvironment. For example, astrocyte-secreted glutathione supplies a source of cysteine that can be utilized by tumor cells [435]. The branched-chain amino acid transaminase 1 (BCAT1) is highly expressed in IDH-wild-type GBM, promoting proliferation through branched-chain amino acids transamination [436]. Amino acid metabolism also intersects with immune regulation. In GBM, YKL-40 potently activates the kynurenine pathway, establishing a robustly immunosuppressive microenvironment [437]. Similar metabolic adaptations occur in BrMs. In NSCLC, BCAT1 promotes EMT and BrM via AlkB homolog 5 (ALKBH5) regulation [438]. Moreover, tumor-derived microRNAs can reshape the neural metabolic niche. For example, miR-199b-5p secreted by brain-metastatic breast cancer cells disrupts GABA synthesis, favouring tumor survival [123]. Therapeutically, targeting amino acid metabolism can enhance immunotherapy efficacy. For example, engineering CAR-T cells with modified branched-chain amino acid metabolism improves their anti-tumor efficacy [439].

Metabolic reprogramming profoundly shapes the immunosuppressive BTME by directly regulating immune cell function [440], [441]. Lactate is a central immunomodulatory metabolite. Export of lactate through monocarboxylate transporters, particularly monocarboxylate transporter (MCT)1 and MCT4, contributes to acidification of the TME [442], [443]. In addition, lactate can promote macrophage reprogramming through histone lactylation, which has been associated with increased IL-10 expression and M2 polarization [444], [445]. Lactate-driven macrophage reprogramming enhances tumor invasion through CCL17 secretion [445]. Interventions, such as GLUT1 inhibition [446] or hexosaminidase B (HEXB) regulation [447], can disrupt this malignant cycle and provide a basis for metabolic-targeted therapies.

Collectively, metabolic reprogramming forms a highly integrated interaction network in which tumor and immune cells establish metabolic symbiosis. Lactate-driven macrophage polarization [419], UDP-P2Y6-mediated TAM regulation [448], and spermidine-induced inhibition of CD8^+^ T cells [448] exemplify this crosstalk. Future research needs to integrate single-cell metabolomics with spatial transcriptomics to resolve metabolic dynamics *in situ* and develop rational combination therapies, particularly strategies combining metabolic interventions with ICIs and hypoxia-targeting approaches.

### The gut microbiota and the gut-brain axis

**Fig. 10:**
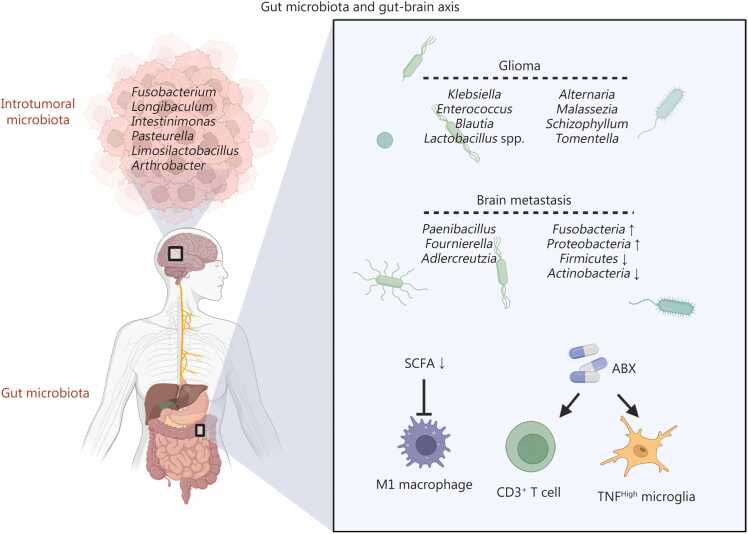
**Common microbial signatures and the gut-brain axis in brain tumors and metastases.** In gliomas, six genera, namely, *Fusobacterium, Longibaculum, Intestinimonas, Pasteurella, Limosilactobacillus, and Arthrobacter*, are significantly enriched. The gut microbiome of glioma patients includes *Klebsiella, Enterococcus, Blautia,* and *Lactobacillus* species, along with fungal genera including *Alternaria*, *Malassezia, Schizophyllum*, and *Tomentella*. In BrMs, distinct microbial signatures are observed, with enrichment of *Paenibacillus*, *Fournierella*, and *Adlercreutzia*. NSCLC patients exhibit significant changes in their gut microbiota composition, with increases in the abundance of *Fusobacteria* and *Proteobacteria*, whereas the abundance of SCFA-producing bacteria, such as *Firmicutes* and *Actinobacteria*, is decreased, particularly during the metastasis stage. Loss of SCFA-producing bacteria represents a shared feature of glioma-associated dysbiosis. SCFAs reverse GBM progression induced by dysbiosis in the BTME by promoting M1 macrophage polarization. ABX gut dysbiosis significantly increased the BrM load while altering the proportions and states of CD3^+^ T cells and TNF^high^ microglia. BrMs. Brain metastases; SCFA. Short-chain fatty acids; GBM. Glioblastoma; BTME. Brain tumor microenvironment; ABX. Antibiotic-induced; TNF. Tumor necrosis factor; NSCLC. Non-small cell lung cancer.

The BTME harbours a distinct tumor-associated microbiota, including bacteria and fungi, which influence tumor progression and treatment response by modulating immune responses, metabolic reprogramming, and inflammatory signaling [449], [450] **(**Fig. 10**)**. Although research in this area remains limited, emerging evidence suggests a non-random microbial signature in brain tumors. In glioma, Li *et al*. [451] identified 6 bacterial genera significantly enriched, including *Fusobacterium*, *Longibaculum*, *Intestinimonas*, *Pasteurella*, *Limosilactobacillus*, and *Arthrobacter*. Notably, *Fusobacterium* and other oncogenic bacteria promote glioma proliferation by upregulating chemokines such as CCL2 and CXCL1 [451]. Gut microbiome profiling in glioma patients reveals enrichment of *Klebsiella*, *Enterococcus*, and *Lactobacillus*, while fungal communities display high interindividual variability, with *Alternaria*, *Malassezia*, and *Schizophyllum* among the most prevalent genera [452]. In contrast, specific taxa such as *Peptostreptococcaceae* and *Oscillospira* may exert protective effects and diminish the risk of glioma [453]. In BrMs, gut microbiome dysbiosis is associated with both compositional and metabolic alterations. For example, in NSCLC patients with BrM, the gut microbiome is characterized by increased abundance of harmful bacteria such as *Fusobacterium* and *Proteus* and reduced short-chain fatty acids (SCFAs)-producing bacteria, including *Firmicutes* and *Actinobacteria* [454]. Consistently, patients with BrMs show increased microbiota diversity, with enrichment of specific genera such as *Paenibacillus, Fournierella,* and *Adlercreutzia*. The metabolomic analyses further revealed changes in SCFAs and vasoactive metabolites, including angiotensin [1], [2], [3], [4], [5], [6], [7], which may regulate immune responses and vascular permeability [455]. Moreover, microbial metabolites such as hippuric acid can also regulate BrM by affecting cancer cell-brain vascular interactions [456]. Together, these findings suggest that microbiome-associated metabolic changes may contribute to the development and progression of BrMs.

Dysbiosis of the gut microbiome is closely linked to brain tumors progression. In patients with meningiomas and gliomas, proinflammatory bacteria such as *Fusobacterium* and *Acinetobacter* species are enriched while beneficial SCFA-producing taxa are reduced [457]. SCFAs, particularly butyrate, alleviate immunosuppression by promoting M1 macrophage polarization [458]. Conversely, antibiotic-induced dysbiosis increases the BrM burden and alters T cells and microglia function [459]. Clinical evidence further supports the therapeutic relevance of the microbiome. In a prospective phase Ⅰ/Ⅱ trial of ndGBM, improved OS within the anti-PD-L1 therapy correlated with distinct immune, mutation, and gut microbiome characteristics [460]. These findings suggest that microbiome modulation could serve as a potential diagnostic and therapeutic target [454].

The microbiome-gut-brain axis constitutes a bidirectional regulatory network involving neural, immune, and metabolic pathways [461], [462]. The gut microbiome regulates BBB permeability, microglial activation, and neuroinflammatory responses through metabolic products such as SCFAs, neurotransmitters (such as 5-HT, GABA), and immune regulatory molecules. Specific compositional changes, such as increased *Proteus* and decreased *Firmicutes*, are significantly associated with GBM occurrence [463]. Certain bacterial genera, including *Alistipes* and *Fusobacterium*, have been implicated in BrM initiation and immune suppression by activating MDSCs and impairing immune surveillance [456], [464]. Moreover, the gut microbiome shapes innate immune responses by regulating microglial function, facilitating immune escape and tumor progression [465].

Microbiome-therapy interactions offer promising opportunities for combination treatment strategies. In pituitary adenomas, fecal microbiota transplantation enhances PD-L1 expression and CD8^+^ T cell infiltration, which inhibits tumor growth [466]. In GBM, local bacterial therapy, such as injectable bacterial-hydrogel constructs, induces tumor necroptosis and activates both innate and adaptive immune responses [467]. Standard treatments also reshape the microbiome. Temozolomide reduces the *Firmicutes*/*Bacteroidetes* ratio [468], whereas sodium butyrate combined with PD-L1 blockade restores beneficial taxa and enhances anti-tumor immunity [469].

Collectively, these findings highlight the microbiome-tumor-immune triad as a critical regulator of brain tumor progression and therapeutic response. Future studies should focus on defining causal microbial species, elucidating microbiome-driven regulation of TAMs and T cells, and integrating metabolic and immunological interventions. Furthermore, the development of personalized microbiome modulation strategies tailored to individual microbiome profiles, including the use of probiotics, fecal microbiota transplantation, or antibiotics in combination treatments, warrants further exploration [469], [470].

### Exosomes

**Fig. 11:**
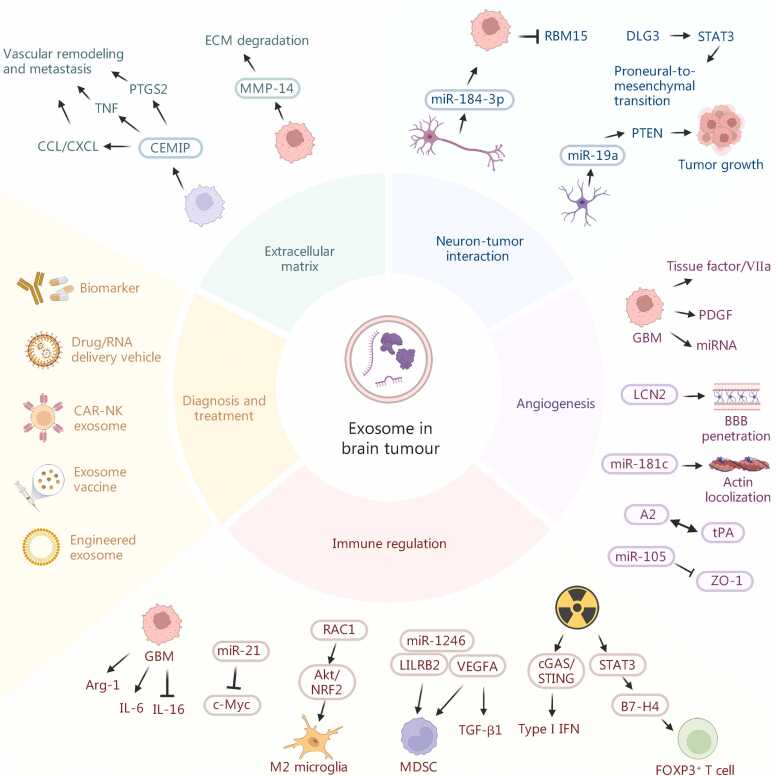
**Exosome-mediated intercellular communication and functional roles in brain tumors.** By transferring proteins, nucleic acids, and lipids, exosomes coordinate tumor-stroma interactions and shape multiple tumor-promoting processes. Exosomes contribute to extracellular matrix remodeling and metastasis by regulating CEMIP, MMP-14, PTGS2, and inflammatory chemokines. They also mediate neuron-tumor interactions, in which neuron- or glia-derived exosomal miRNAs (miR-184-3p and miR-19a) modulate RBM15/DLG3/STAT3 and PTEN signalling, driving PMT and tumor growth. In angiogenesis, tumor-derived exosomes promote vascular activation and permeability through tissue factor/VIIa, PDGF, and miRNA-dependent pathways, enhance BBB penetration via LCN2, and regulate cytoskeletal dynamics and endothelial junction integrity. Exosomes further exert broad immunoregulatory functions by inducing M2-like microglial polarization, enhancing MDSC expansion and suppressive activity via RAC1, miR-1246, LILRB2, VEGFA, and TGF-β1, and modulating adaptive immune responses through cGAS-STING-type Ⅰ interferon and STAT3-dependent pathways. Beyond their biological roles, exosomes represent emerging tools for diagnosis and therapy. CEMIP. Cell migration inducing hyaluronidase 1; MMP-14. Matrix metallopeptidase 14; PTGS2. Prostaglandin-endoperoxide synthase 2; RBM15. RNA binding motif protein 15; DLG3. Discs large MAGUK scaffold protein 3; STAT3. Signal transducer and activator of transcription 3; PDGF. Platelet-derived growth factor; BBB. Blood-brain barrier; LCN2. Lipocalin 2; RAC1. Ras-related C3 botulinum toxin substrate 1; LILRB2. Leukocyte immunoglobulin-like receptor B2; VEGFA. Vascular endothelial growth factor A; TGF-β1. Transforming growth factor-β1; cGAS. Cyclic GMP-AMP synthase; STING. Stimulator of interferon genes.

Exosomes are a subtype of EVs with a diameter of 30-150 nm that mediate intercellular communication within the BTME. Derived from endosomal compartments and enclosed by lipid membranes, exosomes carry diverse cargoes, including proteins, nucleic acids, and lipids, that can reprogram recipient cells and modulate tumor-stroma interactions [471]. They mediate extensive crosstalk between tumor cells and surrounding neurons, glial cells, endothelial cells, and immune cells, thereby promoting tumor progression [472]. Notably, their small size and lipid composition allow them to cross the BBB, making them critical mediators of signal propagation and attractive vehicles for therapeutic delivery in brain tumors **(**Fig. 11**)**.

Exosomes contribute to BTME remodeling through coordinated effects on angiogenesis and BBB integrity. In angiogenesis, GBM-derived exosomes deliver pro-angiogenic molecules, including tissue factor/Ⅶa [473], PDGF [474] and miRNAs [475], activating both VEGF-dependent and VEGF-independent pathways in endothelial cells. Breast cancer-derived exosomes can also induce angiogenesis via annexin A2-endothelial tissue plasminogen activator (tPA) interaction [476]. Exosomes also disrupt BBB integrity by increasing endothelial membrane fluidity and permeability, partly through upregulation of LCN2 [477]. Exosomes derived from breast cancer brain metastatic cells downregulate the tight junction protein zonula occludens-1 (ZO-1) via miR-105 [478] and alter actin localization through miR-181c [479], further compromising the BBB integrity and facilitating tumor cell infiltration into the brain.

Exosomes and their contents are important signal transduction mediators and key substances that help shape the immunosuppressive BTME. GBM-derived exosomes induce M2-like microglial polarization [480]. Exosomal miR-21 and Ras-related C3 botulinum toxin substrate 1 (RAC1) further inhibit anti-tumor immunity by targeting cellular myelocytomatosis oncogene (c-Myc) and activating the Akt/NRF2 pathway, respectively [481], [482]. Furthermore, exosomes promote the recruitment and immunosuppressive differentiation of MDSCs through multiple cargos, including VEGFA, miR-1246, and LILRB2 [194]. Radiotherapy reshapes exosomal composition. For example, irradiated GBM cells secrete B7-H4-enriched exosomes that promote FoxP3^+^ T cell differentiation and radioresistance via STAT3 activation [483]. In contrast, radiotherapy-derived stem cell exosomes enhance TLSs formation and type I interferon signaling through the cGAS-STING pathway, improving anti-tumor immunity [483].

Exosomes also mediate bidirectional communication between tumors and neural cells. Neuronal activation increases exosomal miR-184-3p, which suppresses RBM15 in GSCs, reduces m^6^A modification of *DLG3* mRNA, and drives PMT via STAT3 activation [99]. Furthermore, astrocyte-derived exosomes similarly promote BrM by transferring miR-19a to breast cancer cells, downregulating phosphatase and tensin homolog (PTEN) and promoting tumor proliferation [484].

In addition, exosomes regulate ECM remodeling and vascular adaptation. GBM-derived exosomes induce MMP-14 in microglia, enhancing ECM degradation [485]. In BrM, exosomal cell migration-inducing and hyaluronan-binding protein (CEMIP) upregulates proinflammatory cytokines and chemokines, promoting cerebrovascular remodeling and metastatic colonization [486].

Beyond their biological roles, exosomes hold substantial diagnostic and therapeutic potential. Exosomes can serve as biomarkers for liquid biopsy, enabling early detection and disease monitoring [487]. Therapeutically, they have been explored as delivery vehicles for mRNA and miRNA [488], [489]. Engineered exosomes can remodel the immunosuppressive microenvironment [490]. In addition, both GBM-derived exosome vaccines [491] and CAR-NK cell-derived exosomes [492] have demonstrated promising anti-tumor potential.

Despite rapid progress, several challenges limit clinical translation, including exosomal heterogeneity, lack of standardized isolation methods, and unresolved dosing and safety concerns. Furthermore, a key challenge for future precision interventions lies in suppressing tumor-promoting exosome functions while preserving physiological EV-mediated communication in the CNS. Overall, exosomes represent a critical frontier in understanding BTME regulation and the development of novel therapeutic strategies.

## Research techniques and models for BTME

### New imaging technologies

**Fig. 12:**
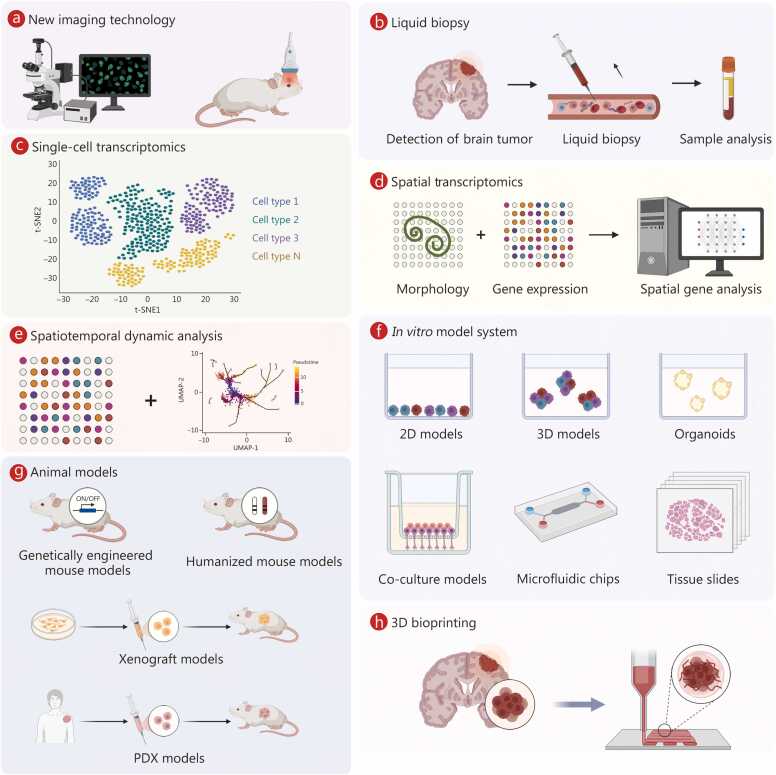
**Research techniques and models for brain tumor microenvironment**. **a** New imaging technology. **b** Liquid biopsy. **c** Single-cell transcriptomics. **d** Spatial transcriptomics. **e** Spatiotemporal dynamic analysis. **f***In vitro* model system. **g** Animal models. **h** 3D bioprinting. 2D. Two-dimensional; 3D. three-dimensional; PDX. Patient-derived xenograft.

Recent advances in imaging technologies have provided powerful tools for investigating BTME. High-resolution microscopy techniques, such as super-resolution stimulated emission depletion microscopy and advanced ultrasound imaging, enable nanoscale visualization of molecular distributions and interactions in BTME [493], [494], [495], [496] **(**Fig. 12**a)**. Carbon dot (CD)-based probes, particularly long-wavelength red-emitting CDs (R-CDs), exhibit excellent tumor-targeting ability, biocompatibility, and deep-tissue imaging potential for GBM [497]. Moreover, multifunctional probes, nanoparticles, and micelles integrate imaging with therapeutic delivery [498], [499], [500]. TMZ-loaded nanobubbles combined with persistent luminescence nanoparticles allow image-guided GBM therapy with improved tissue penetration and signal-to-noise ratio [501].

### Liquid biopsy

Liquid biopsy provides a minimally invasive approach for real-time monitoring of tumor dynamics by analysing circulating tumor DNA (ctDNA), circulating tumor cells, and exosomes in blood or cerebrospinal fluid [502], [503], [504], [505] **(**Fig. 12**b)**. Microbial exosome profiling based on 16S rDNA sequencing has enabled the construction of diagnostic models for brain tumors with high accuracy (area under the curve 0.93) [506]. In parallel, nanotechnology has enhanced liquid biopsy sensitivity; superlattice-based sensors can detect GBM CSCs with high sensitivity and specificity using minimal blood volume [507]. Integrative approaches combining ctDNA and the T cell repertoire were used to predict radiotherapy response in the NSCLC BrM patients [508]. Moreover, focused ultrasound-enabled liquid biopsy disrupts the BBB, releasing tumor-derived DNA into the circulation and improving detection efficiency [509].

### Single-cell transcriptomics

Single-cell sequencing technologies have revolutionized the study of BTME heterogeneity [139], [154], [510] **(**Fig. 12**c)**. Single-cell studies further show that macrophages dominate the GBM microenvironment, followed by T cells and neutrophils, whereas NK and natural killer T (NKT) cells are relatively scarce [47]. Cellular communication and interactions represent fundamental aspects of the BTME [511]. Single-cell technologies, including single-cell assays for transposase accessible chromatin (ATAC) sequencing, enable high-resolution profiling of transcriptional and epigenetic states across diverse cell populations [512]. These approaches have revealed previously unrecognized cell subtypes and dynamic cell states within the BTME [415]. For example, the integration of single-cell datasets and large-scale population data has identified the specific accumulation of CXCL13^+^CD39^+^ tumor-reactive T (pTRT) cells in BrM patients [235].

### Spatial transcriptomics

Spatial transcriptomics is a major methodological advance by preserving spatial context while profiling gene expression [42], [171] **(**Fig. 12**d)**. Unlike dissociative single-cell approaches, spatial transcriptomics enables the analysis of cell-cell interactions, vascular niches, and immune-tumor spatial organization within the intact BTME. In GBM, spatial transcriptomics has identified spatially distinct transcriptional programs, demonstrating how stress-free regions facilitate subtype transitions, with key features of hypoxia and immune suppression phenotypes [420]. Complementary technologies, such as multiplex immunofluorescence and mass spectrometry imaging, further enable high-resolution mapping of protein expression and metabolic states, advancing spatial metabolomics and signaling analysis [513], [514]. Moreover, spatial RNA sequencing has been used on primary and metastatic NSCLC samples, providing a comprehensive spatial transcriptomic map [151].

### Spatiotemporal dynamic analysis

The spatiotemporal analysis is essential for capturing the dynamic evolution of the BTME during tumor initiation, progression, and treatment response [515], [516], [517]. Long-term *in vivo* imaging allows real-time observation of cellular behaviour and signaling dynamics [518] **(**Fig. 12**e)**. Comparative proteomic analyses between ndGBM and rGBM reveal increased immune cell infiltration and ECM remodeling in rGBM [519]. Spatiotemporal approaches have provided important insights into the dynamic cellular organization and interactions within brain tumors. Through a dynamic analysis of spatiotemporal transcription, Comba *et al*. [520] identified oncostreams, which are dynamic multicellular bundles of spindle-shaped and neatly arranged cells with mesenchymal properties that are regulated by collagen type I alpha 1 chain (COL1A1). Consistently, time-resolved single-cell RNA sequencing revealed tumor-macrophage crosstalk promotes mesenchymal transition in GBM through the EZH2-FOSL2-CCL2 axis [521]. In parallel, two-photon imaging technology provides high-resolution spatiotemporal imaging of immune-tumor interactions [522], [523], demonstrating correlations between TAM/microglia composition and BrM stages [523], [524], and enabling longitudinal imaging of microglia-neuron interactions [525].

Multimodal integration is key to understanding the complexity of the BTME. Combining genomics, transcriptomics, proteomics, and metabolomics enables a comprehensive understanding of BTME composition and function [76], [420], [526], [527], [528]. Systems biology approaches, such as network analysis and dynamic modelling, have improved our understanding of the interactions and regulatory mechanisms among various components in the BTME.

### *In vitro* model system

*In vitro* model systems provide controllable platforms to reconstruct key features of the BTME and to dissect TME interactions. Organoids reconstruct certain features of the BTME [529], [530]. Coculturing tumor cells with stromal cells allows simulation of cell-cell interactions in the BTME **(**Fig. 12**f)**. Brain organoids containing microglia (MiCBOs) recapitulate essential immune-neural interactions, containing mature, motile microglia, and various subtypes of neurons [531]. Eichmüller *et al*. [532] constructed a human brain organoid model of the tuberous sclerosis complex, identified late-stage intermediate neural progenitor (CLIP) cells that drive tumor development and cortical malformations. Recent advances include myelinated human brain organoids with integrated microglia, which demonstrate microglia-dependent remyelination and enable evaluation of pro-myelinating compounds [533].

Microfluidic platforms offer complementary advantages by enabling precise control over fluid dynamics, spatial organization, and cellular composition [534], [535], [536]. This system is particularly suitable for investigating angiogenesis and drug delivery processes under physiologically relevant flow conditions. Perivascular niche-on-chip supports three-dimensional (3D) growth of patient-derived GSCs and fetal neural stem cells [537]. The emergence of microfluidic bionic chips can simulate multiorgan metastatic processes of tumors simultaneously [538].

Organotypic slice culture preserves the native 3D architecture, ECM, and intercellular connections of brain tissue, maintaining region-specific spatial conformation and cellular heterogeneity, thereby recapitulating the complexity of BTME [539]. As this model also retains neuronal electrical activity and synaptic function, it provides a physiologically relevant platform for directly investigating interactions between brain tumors and neural circuits. The *in vitro* organotypic brain slice cultures retain diverse cellular components within the TME, including astrocytes, microglia, and infiltrating immune cells, faithfully simulating TME crosstalk. In the context of personalized medicine, patient-derived tissue slices serve as a valuable tool for drug sensitivity testing and therapeutic strategy development [540]. Within tissue slices, computational tools such as the spatial transcriptomic algorithm GASTON enhance the resolution of microenvironmental heterogeneity [541]. These platforms have yielded important mechanistic insights into tumor invasion and recurrence. For example, brain slice-tumor co-culture systems have revealed that pSTAT3^+^ reactive astrocytes promote invasive growth through CHI3L1 secretion in BrM models [542]. Human brain slice cultures confirmed that F-box protein 2 (*FBXO2*) knockout suppresses tumor cell infiltration [543].

BrM modeling remains particularly challenging [9]. Conventional approaches using primary lung cancer or breast cancer cell lines fail to capture the selective pressures of brain colonization [544]. The brain metastatic microenvironment differs substantially from the primary tumor, and metastatic cells undergo extensive selection and adaptation during dissemination [545]. To better mimic this process, brain-tropic tumor cell models are commonly generated through serial *in vivo* selection, involving repeated systemic injection and recovery of metastatic lesions to enrich for brain-adapted clones [546]. However, these models are typically established in immunodeficient mice and therefore lack immune system contributions. Furthermore, treatment-resistant tumor cells offer a valuable platform for studying therapeutic resistance and tumor evolution within the BTME [547].

### Animal models

Animal models remain indispensable for studying brain tumor initiation, progression, and therapeutic response [548]. Different animal models provide complementary advantages for addressing distinct biological questions. Genetically engineered mouse models recapitulate brain tumors development in an immunocompetent setting, enabling mechanistic studies that closely reflect human disease [549]. Furthermore, humanized mouse models, generated by engrafting human immune systems into immunodeficient hosts [550], enable studying immunotherapy and tumor-immune interactions of human brain tumors in a human-relevant context **(**Fig. 12**g)**.

Xenograft models, including patient-derived xenografts (PDXs), preserve tumor heterogeneity and are suitable for studying personalized treatment strategies [548]. In recent years, PDX models have been widely used in drug screening [551], [552], [553]. Combining repeated biopsies with ultrafast cycling technology further enables longitudinal monitoring of immunological changes during tumor evolution [554].

### 3D bioprinting

3D bioprinting enables precise spatial organization of cells and ECM components, facilitating the construction of tumor models that more closely recapitulate *in vivo* conditions [555], [556], [557], [558] **(**Fig. 12**h)**. For example, 3D microfluidic GBM models have been employed to investigate tumor invasion and metastatic dissemination [559]. Additionally, 3D bioprinting has successfully recapitulated the phenotypic heterogeneity and drug resistance in GBM [560].

## Advancing therapeutic strategies through BTME targeting

The deeper understanding of the BTME has opened new avenues for therapeutic development. Current BTME-targeted strategies include inhibiting tumor proliferation and angiogenesis and modulating immune responses [561], [562], [563]. Anti-angiogenic therapies, particularly VEGF inhibitors, have shown clinical benefit. However, the presence of the BBB and the unique immunosuppressive microenvironment significantly limit the efficacy of ICIs in brain tumors. In recent years, emerging approaches such as CAR-T cell therapy targeting brain tumor-specific antigens and oncolytic virus therapy combining direct tumor lysis with immune activation, offer a new option for brain tumors treatment [564], [565].

Patients with BrMs have historically been analyzed as a subpopulation within studies of primary tumors. Nevertheless, advances in targeted therapies for lung cancer have translated into significant progress in BrMs treatment [566], [567]. Based on targeted strategies, the integration of radiotherapy, chemotherapy, and antiangiogenic agents is increasingly explored [568], [569], [570]. For patients without actionable driver mutations or those who develop resistance to targeted therapies, chemotherapy remains a cornerstone of management. In such cases, the addition of immunotherapy or antiangiogenic therapies is under investigation to further enhance treatment efficacy [561], [562], [571]. Dual ICIs have attracted particular interest and represent a current focus of translational research [563], [572], [573]. Radiotherapy remains a fundamental method for local disease control, with novel approaches such as prophylactic cranial irradiation and hippocampal avoidance whole-brain radiotherapy (HA-WBRT) gaining considerable attention [574], [575], [576]. Despite these advances, the development of phase Ⅲ clinical trial development for melanoma and breast cancer BrMs remains slower than that for lung cancer **(**Table 1**)** [161], [249], [561], [562], [563], [566], [567], [568], [569], [570], [571], [572], [573], [574], [575], [576], [577], [578], [579], [580], [581], [582], [583], [584], [585], [586], [587], [588], [589], [590], [591], [592], [593], [594], [595], [596], [597], [598], [599], [600], [601], [602], [603].

**Table 1:** The latest phase Ⅲ clinical trials for brain tumors (2021 to date).

Therapy	Clinical trial ID	Phase	Inclusion patients (*n*)	Interventions	Results	AEs	References
Lung cancer BrM							
Chemo+anti-PD-1+anti-VEGF	NCT05184712	Ⅲ	EGFRm NSCLC (*n=*37)	Ivonescimab+Chemo	PFS for BrM: *HR=*0.40; mOS: not mature	≥3 grade treatment-emergent AEs of entire group: Ivonescimab group: 61.5%; Placebo group: 49.1% (most common: Chemo-related)	[561]
Chemo+ICI+anti-VEGF	NCT02366143	Ⅲ	Treated and stable BrM (*n=*1202)	ABCP/ACP/BCP	100 patients (8.3%) developed new BrM, ABCP was found with an improvement toward delayed time to development compared to BCP: *HR=*0.68, 95% CI 0.39–1.19	NA	[562]
	NCT03802240	Ⅲ	Locally advanced or metastatic EGFRm NSCLC who had disease progression after receiving EGFR-TKI (*n=*444)	Sintilimab+IBI305+pemetrexed+cisplatin (*n=*148); sintilimab+pemetrexed+cisplatin (*n=*145); Pemetrexed+cisplatin (*n=*151)	*HR* for PFS in the sintilimab+IBI305+Chemo group: 0.48	≥3 grade treatment-related AEs: decreased neutrophil count (20% in the sintilimab+IBI305+Chemo group vs. 18% in the Chemo alone group), decreased white blood cell count (11% vs. 9%), and anemia (12% vs. 10%)	[571]
Immunotherapy	NCT03088540	Ⅲ	NSCLC BrM with PD-L1 ≥50 (*n=*69)	Cemiplimab monotherapy vs. platinum-based doublet Chemo	mOS: 52.4 months (cemiplimab) vs. 20.7 months (Chemo), *HR=*0.40 (*P=*0.0031); mPFS: 12.5 months vs. 5.3 months, *HR=*0.33 (*P=*0.0002)	NA	[572]
Dual-immunotherapy	NCT02477826	Ⅲ	Treatment-naive adults with stage IV or recurrent NSCLC without EGFR or ALK alterations [BrM at baseline *n=*202 (nivolumab+ipilimumab *n=*68; Chemo *n=*66)]	Nivolumab+ipilimumab/Chemo	Nivolumab+ipilimumab group vs. Chemo group: 5-year OS: *HR=*0.63, 95% CI 0.43–0.92; 5-year systemic PFS: 12% vs. 0%; 5-year iPFS: 16% vs. 6%; New brain lesions: 4% vs. 20%	NA	[573]
	NCT02869789	Ⅲ	Untreated advanced NSCLC with BrM (*n=*49)	Nivolumab+ipilimumab	3-year OS: 20.5%	Most common grade 3–4 immune-mediated AEs: diarrhea/colitis (3.5%)	[563]
RT	NCT02397733	Ⅲ	SCLC (71.3% with limited disease) (*n=*150)	PCI/HA-PCI	Delayed free recall at 3 months: HA-PCI group: 5.8%; PCI group: 23.5%; The incidence of BrM, OS, and QoL was not significantly different	NA	[574]
	NCT01780675	Ⅲ	SCLC (*n=*168)	Standard PCI/HA-PCI	Standard PCI group vs. HA-PCI group: Hopkins Verbal Learning Test-Revised total recall score at 4 months: 29% vs. 28%; Cumulative incidence of BrM at 2 years: 20% (95% CI 12–29) vs. 16% (95% CI 7–24) OS: No significant difference	HA-PCI did not reduce the probability of cognitive decline in patients with SCLC compared with conventional PCI	[575]
	NCT02355613	Ⅲ	BrM from primary solid tumor [1–4 BrMs, up to 30 mm in maximum diameter [*n=*251 (arm A, *n=*121; arm B, *n=*130)]	Arm A: multi-source gamma-ray stereotactic platform; Arm B: Linac	1-year cumulative incidence of symptomatic (grade 2–3) RN: arm A 9.3% (95% CI 6.2–13.8), arm B 3.8% (95% CI 1.9–7.4); Median time to RN: arm A 6.9 months, arm B 15.9 months	56 symptomatic RN: 29 in arm A, 27 in arm B	[576]
	NCT01372774	Ⅲ	Long-term survivors with 1–4 BrMs [*n=*54 (SRS *n=*27; WBRT *n=*27)]	SRS/WBRT	Cognitive deterioration was less frequent with SRS (37%–60%) compared with WBRT (75%–91%) at all time points; Total intracranial control at 12 months: SRS group 40.7%; WBRT group 81.5%	NA	[579]
Targeted therapy	NCT02737501	Ⅲ	ALK inhibitor-naive advanced ALK-positive NSCLC [*n=*275 (brigatinib *n=*137; crizotinib *n=*138)]	Brigatinib	Brigatinib improved OS in patients with baseline BrM (*HR=*0.43, 95% CI 0.21–0.89)	NA	[566]
	NCT03052608	Ⅲ	Treatment-naive, ALK-positive NSCLC [*n=*149 (BrM at baseline: *n=*37)]	Lorlatinib	PFS: Lorlatinib group: not reached; Crizotinib group: 9.3 months (95% CI 7.6–11.1); BrM at baseline: time to intracranial progression: *HR=*0.10, 95% CI 0.04–0.27	≥3 grade AEs: Lorlatinib group: 76% (most commonly due to altered lipid levels)	[567]
	NCT04194944	Ⅲ	Untreated advanced RET fusion-positive NSCLC [*n=*212 (measuerable BrM *n=*29)]	Selpercatinib/Chemo+pembrolizumab	Intracranial response: Selpercatinib group: 82%; Chemo+pembrolizumab group: 58%; CR: Selpercatinib group: 35%; Chemo+pembrolizumab group: 17%	A higher incidence with selpercatinib.	[580]
	NCT03052608	Ⅲ	Untreated advanced ALK-positive NSCLC (*n=*78)	Lorlatinib/Crizotinib	PFS at 12 months: With BrM at baseline: lorlatinib group 78%; crizotinib group 22%; Without BrM at baseline: lorlatinib group 78%; crizotinib group 45%; Cumulative incidence of CNS progression at 12 months: With BrM at baseline: lorlatinib group 7%; crizotinib group 72%; Without BrM at baseline: lorlatinib group 1%; crizotinib group 18%	CNS AEs incidence: Lorlatinib group: 35% (most of grade 1 severity); Patient-reported QoL: no clinical difference	[581]
	NCT02767804	Ⅲ	Advanced ALK-positive NSCLC [*n=*290 (ensartinib: *n=*145; crizotinib: *n=*145)]	Ensartinib/Crizotinib	Intracranial response rate (BrM at baseline): Ensartinib group: 63.6% (7/11); Crizotinib group: 21.1% (4/19)	Treatment-related serious AEs: Ensartinib group: 7.7%; Crizotinib group: 6.1%	[582]
	NCT04009317	Ⅲ	Untreated advanced ALK-positive NSCLC [*n=*88 (envonalkib *n=*130; crizotinib *n=*130)]	Envonalkib/Crizotinib	Envonalkib group vs. crizotinib group: CNS*-ORR*: 78.95% vs. 23.81%; mCNS-TTP: 30.32 months vs. 8.28 months	≥3 grade AEs: Envonalkib group: 55.73%; Crizotinib group: 42.86%	[583]
	NCT04028778	Ⅲ	Untreated EGFRm advanced NSCLC (*n=*99)	Anlotinib+gefitinib/Placebo+gefitinib	mPFS: Anlotinib group: 13.8 months; Placebo group: 8.3 months; *HR=*0.47	≥3 grade treatment-emergent AEs: Anlotinib group: 49.7%; Placebo group: 31.0%	[584]
	NCT03653546	Ⅲ	Untreated EGFRm advanced NSCLC with non-irradiated symptomatic or asymptomatic CNS metastases (*n=*439)	Zorifertinib	Zorifertinib significantly prolonged iPFS vs. control (RECIST1.1: *HR=*0.467, 95% CI 0.352–0.619; RANO-BrM: *HR=*0.627, 95% CI 0.466–0.844)	Manageable AEs	[585]
	NCT03849768	Ⅲ	EGFRm advanced NSCLC with BrM [*n=*429 (cFAS *n=*106; cEFR *n=*60)]	Aumolertinib/Gefitinib	Aumolertinib group vs. Gefitinib group: mCNS PFS (cFAS): 29.0 vs. 8.3 months; mCNS PFS (cEFR): 29.0 vs. 8.3 months; CNS *ORR* (cEFR): 85.7% vs. 75.0%	No new safety findings	[586]
Targeted therapy+Chemo	NCT01951469	Ⅲ	Untreated EGFRm NSCLC with BrM [*n=*161 (gefitinib+Chemo *n=*80; gefitinib alone *n=*81)]	Gefitinib+Chemo/Gefitinib alone	iPFS: Gefitinib+Chemo group: 15.6 months, 95% CI 14.3–16.9; Gefitinib alone group: 9.1 months, 95% CI 8.0–10.2, *HR=*0.36 OS: Gefitinib+Chemo group: 35.0 months; Gefitinib alone group: 28.9 months; *HR=*0.65. iORR: 85% vs. 63%	≥3 grade AEs: higher incidence in gefitinib plus Chemo group (most common: chemo-related), most of which were manageable	[587]
Targeted therapy+Chemo	NCT04035486	Ⅲ	EGFRm advanced NSCLC [*n=*557; cFAS (≥one measurable and/or no measurable CNS lesion) *n=*222, ombination *n=*118 vs. monotherapy *n=*104); cEFR (≥1 measurable target CNS lesion) *n=*78 (combination *n=*40; monotherapy *n=*38)]	Osimertinib+platinum-pemetrexed (combination)/Osimertinib (monotherapy)	Combination group vs. Monotherapy group: cFAS: CNS PFS: *HR=*0.58, 95% CI 0.33–1.01; CNS *ORR:* 73% vs. 69%; CNS CR rates: 59% vs. 43%; cEFR: CNS *ORR*: 88% vs. 87%; CNS CR rates: 48% vs. 16%	NA	[568]
Targeted therapy+anti-VEGF	NCT02759614	Ⅲ	Untreated EGFRm advanced NSCLC (erlotinib+bevacizumab *n=*157; Erlotinib *n=*154)	Erlotinib/Bevacizumab+erlotinib	BrM subgroup analysis: Bevacizumab+erlotinib improved PFS (*HR*=0.48, 95% CI 0.27–0.84; *P=*0.008)	≥3 grade treatment-emergent AEs: Bevacizumab+erlotinib group: 54.8% (86/157); Erlotinib group: 26.1% (40/154)	[570]
Targeted therapy+RT	NCT02882984	Ⅲ	Treatment-naïve EGFRm NSCLC with BrM (SRT *n=*44; WBRT *n=*41)	SRS/WBRT+first–generation TKI	iPFS at 18 months: WBRT group: 9.5%; SRT group: 10.2%. miPFS: WBRT group: 21.4 months; SRT group: 22.3 months, *P* > 0.05	Low enrollment: 76% patients not wishing to risk neurocognitive decline from WBRT	[588]
	NCT01887795	Ⅲ	NSCLC with≥2 BrMs (WBRT+erlotinib *n=*109; WBRT *n=*115)	WBRT+erlotinib/WBRT	WBRT+erlotinib group vs. WBRT group: In the whole group: iPFS: 11.2 months vs. 9.2 months (*P=*0.601); PFS: 5.3 months vs. 4.0 months (*P=*0.825); OS: 12.9 months vs. 10.0 months (*P*=0.545). In EGFRm patients: iPFS: 14.6 months vs. 12.8 months (*P=*0.164); PFS: 8.8 months vs. 6.4 months (*P=*0.702); OS: 17.5 vs. 16.9 months (*P=*0.221). Cognitive function (Mini-Mental State Examination score): no significant differences	WBRT+erlotinib group vs. WBRT group: ≥ grade 3 AEs: 11.2% vs. 1.7%	[569]
Melanoma BrM							
Chemo+ICI/ICI+ICI	NIBIT-M2	Ⅲ	Untreated asymptomatic melanoma with BrM (Fotemustine *n=*27; Ipilimumab+fotemustine *n=*26; Ipilimumab+nivolumab *n=*27)	Fotemustine/Ipilimumab+fotemustine/Ipilimumab+nivolumab	Fotemustine group vs. Ipilimumab+fotemustine group vs. Ipilimumab+nivolumab group: mOS: 8.5 months (95% CI 4.8–12.2) vs. 8.2 months (95% CI 2.2–14.3) vs. 29.2 months (95% CI 0–65.1); 4-year OS: 10.9% vs. 10.3% vs. 41.0%	Fotemustine group vs. Ipilimumab+fotemustine group vs. Ipilimumab+nivolumab group: ≥3 grade AEs: 48% vs. 69% vs. 30%	[577]
	NIBIT-M2	Ⅲ	Untreated asymptomatic melanoma with BrM (*n=*76)	Fotemustine/Ipilimumab+fotemustine/Ipilimumab+nivolumab	Fotemustine group vs. Ipilimumab+Fotemustine group vs. Ipilimumab+Nivolumab group: 7-year OS: 10.0% (95% CI 0–22.5) vs. 10.3% (95% CI 0–22.6) vs. 42.8% (95% CI 23.4–62.2)	All groups preserve Health-Related QoL	[589]
Targeted therapy	NA	Ⅲb	Unresectable stage Ⅲc or IV BRAFV600-mutant melanoma with or without BrM [*n=*856; BrM at baseline *n=*275 (32%)]	Dabrafenib+trametinib	mPFS: 5.68 months, 95% CI 5.29–6.87	NA	[590]
Breast cancer BrM							
ADC	NCT03529110	Ⅲ	HER2-positive breast cancer BrM previously treated with Trastuzumab and a taxane [*n=*82 (T-DXd group *n=*43, 16.5%; T-DM1 group *n=*39, 14.8%)]	Trastuzumab deruxtecan (T-DXd)/Trastuzumab emtansine (T-DM1)	mPFS: T-DXd group: 15.0 months (95% CI 12.5–22.2); T-DM1 group: 3.0 months (95% CI 2.8–5.8). *HR=*0.25 *ORR*: T-DXd group: 67.4%; T-DM1 group: 20.5%. *iORR*: T-DXd group: 65.7%; T-DM1 group: 34.3%.	NA	[578]
Chemo	NCT02915744	Ⅲ	Advanced BC with stable and treated BrM [*n=*178 (Etirinotecan pegol *n=*92, 51.7%; Chemo *n=*86, 48.3%)]	Etirinotecan pegol/Chemo	Etirinotecan pegol group vs. Chemo group: mOS: 7.8 months vs. 7.5 months; mPFS for CNS metastases: 3.9 months vs. 3.3 months	Comparable safety profiles between the groups	[591]
Glioma							
Targeted therapy	NCT04164901	Ⅲ	Residual or recurrent grade 2 *IDH*-mutant glioma [*n=*331 (Vorasidenib *n=*168; Placebo *n=*163)]	Vorasidenib	Vorasidenib significantly improved mPFS: Vorasidenib group 27.7 months vs. Placebo group 11.1 months, *HR=*0.39	≥3 grade AEs: Vorasidenib 22.8% vs. Placebo 13.5%	[592]
	NCT05580562	Ⅲ	Newly diagnosed H3 K27M-mutant diffuse glioma	Placebo/Once-weekly dordaviprone/twice-weekly dordaviprone	NA	NA	[593]
Chemo+cytokines	NCT01765088	Ⅲ	ndHGG (WHO grade 3/4, *n=*199)	Experimental group: TMZ+interferon-α; Control group: TMZ	2-year OS: the experimental group was significantly better than the control group (26.7 months vs. 18.8 months, *HR=*0.64, *P=*0.005); MGMT unmethylated subgroup: the OS of the experimental group was significantly prolonged (24.7 months vs. 17.4 months, *HR=*0.57, *P=*0.008)	More common in the experimental group: epileptic seizures (2% grade 1), flu-like symptoms (5% grade 2, *P=*0.02). Overall toxicity was manageable	[594]
Chemo+RT	NCT00626990	Ⅲ	IDH1/2 wildtype GBMs [*n=*159 (RT group *n=*47; RT+TMZ *n=*112)]	RT/RT+TMZ	OS: *HR=*1.19 (95% CI 0.82–1.71); PFS: *HR=*0.87 (95% CI 0.61–1.24), TMZ did not improve survival	NA	[595]
	NCT00887146	Ⅲ	Newly diagnosed 1p/19q WHO grade Ⅲ oligodendroglioma (*n=*36)	Arm A: RT (59.4 Gy); Arm B: RT with concomitant and adjuvant TMZ; Arm C: TMZ alone	PFS: Arm C: 83.3% (10/12) patients progressed; RT group (arm A+B): 37.5% (9/24) progressed, *HR=*3.12; Although there was no statistically significant difference in *OS*, a lower mortality rate was observed in the RT group	≥3 grade AEs: Arm A: 25%; Arm B: 42%; Arm C: 33%	[596]
	EORTC 1709	Ⅲ	ndGBM (*n=*749)	Standard therapy group: TMZ+RT+adjuvant TMZ Marizomib group: standard therapy+marizomib	Standard therapy group vs. Marizomib group: mOS: 17.0 months vs. 16.5 months; mPFS: 6.0 months vs. 6.3 months. MGMT promoter-unmethylated group: OS: 14.5 months vs. 15.1 months	More grade 3/4 treatment-emergent AEs were observed in the marizomib arm	[597]
Chemo+RT+ADC	NCT02573324	Ⅲ	ndGBMs with *EGFR* gene amplification (*n=*639)	RT+TMZ+depatuxizumab mafodotin (Depatux–m)/Placebo	Depatux-m group: mOS 18.9 months; mPFS 8.0 months. Placebo group: mOS 18.7 months; mPFS 6.3 months	AEs: Corneal epitheliopathy: 94% of Depatux-m-treated patients (61% grade 3–4), causing 12% to discontinue. No new important safety risks	[598]
Chemo+RT+ICI	NCT02667587	Ⅲ	ndGBM with methylated MGMT promoter (*n=*716)	RT+TMZ+Nivolumab	Nivolumab added to RT+TMZ did not improve PFS or OS in patients with ndGBM with methylated or indeterminate MGMT promoter	≥3 grade treatment-related AEs: experimental group 52.4% vs. placebo group 33.6%	[249]
Chemo combination	NCT02678975	Ⅱ/Ⅲ	≥ 18 years, first rGBM (*n=*88)	Disulfiram+copper+Chemo/Chemo	Disulfiram+copper+Chemo group vs. Chemo group: 6 months survival: 44% vs. 62% (*P*=0.10) mOS: 5.5 months vs. 8.2 months mPFS: 2.3 months vs. 2.6 months	Disulfiram+copper+Chemo group vs. Chemo group: ≥ grade 3 AEs: 34% vs. 11% (*P*=0.02); Severe AEs: 41% vs. 16% (*P=*0.02); 10 patients (24%) discontinued disulfiram treatment because of AEs	[599]
RT+ICI	NCT02617589	Ⅲ	ndGBM with unmethylated MGMT promoter [*n=*560 (RT+nivolumab *n=*280; RT+TMZ *n=*280)]	RT+nivolumab/RT+TMZ	RT+TMZ demonstrated a longer mOS than RT+Nivolumab	≥3 grade treatment-related AEs: RT+nivolumab group: 21.9% RT+TMZ group: 25.1%	[600]
DC vaccine (DCVax-L)	NCT00045968	Ⅲ	ndGBM and rGBM (*n=*331)	DCVax-L+TMZ	mOS: ndGBM: DCVax-L group 19.3 months vs. Placebo group 16.5 months; *HR=*0.8; rGBM: DCVax-L group 13.2 months vs. Placebo group 7.8 months; *HR=*0.58	NA	[161]
Others							
RT	NCT00085735	Ⅲ	Age 3–21 years with average-risk MB (*n=*549)	CSI+PFRT vs. CSI+IFRT; Radiation dose: SDCSI (23.4 Gy)/LDCSI (18 Gy)	5-year EFS: IFRT vs. PFRT: 82.5% vs. 80.5%, IFRT was not inferior to PFRT; LDCSI vs. SDCSI: 71.4% vs. 82.9%; LDCSI was inferior to SDCSI	The SDCSI group exhibited greater late declines in neurocognitive outcome	[601]
Chemo+RT	JCOG1114C	Ⅲ	PCNSL (*n=*134)	Arm A: HD-MTX and WBRT (30 ±10) Gy boost; Arm B: HD-MTX+WBRT±boost with concomitant and maintenance TMZ for 2 years	2-year OS: arm A, 86.8% (95% CI 72.5–94.0) vs. arm B, 71.4% (95% CI 56.0–82.2); *HR=*2.18 (95% CI 0.95–4.98); The predicted probability of showing the superiority of arm B at the final analysis is estimated to be 1.3%. The study was terminated early due to futility	NA	[602]
Dual-immunotherapy	NCT02982954	Ⅲb/Ⅳ	Untreated advanced renal cell carcinoma with BrMs (*n=*28)	Nivolumab+ipilimumab+nivolumab maintenance	*ORR*: 32% (95% CI 14.9–53.5); Median duration of response: 24.0 months; 4 of 8 responders remained without reported progression; iPFS rates: 25%; mPFS: 9.0 months (95% CI 2.9–12.0); mOS: not reached (95% CI 14.1 to not estimable)	No grade 5 immune-mediated AEs. Most common grade 3 and 4 immune-mediated AEs: diarrhea/colitis and hypophysitis, rash, hepatitis, and diabetes mellitus	[603]

PD-1. Programmed death-1; VEGF. Vascular endothelial growth factor; ICI. Immune checkpoint inhibitor; RT. Radiotherapy; ADC. Antibody-drug conjugate; DC. Dendritic cell; DCVax-L. Autologous tumor lysate-loaded dendritic cell vaccine; Chemo. Chemotherapy; EGFR. Epidermal growth factor receptor; EGFRm. *EGFR* mutation; NSCLC. Non-small cell lung cancer; BrMs. Brain metastasis; PD-L1. Programmed death-ligand 1; ALK. Anaplastic lymphoma kinase; RET. Rearranged during transfection; TKI. Tyrosine kinase inhibitor; SCLC. Small cell lung cancer; SRS/SRT. Stereotactic radiosurgery; WBRT. Whole-brain radiotherapy; CNS. Central nervous system; cFAS. CNS full analysis set; cEFR. CNS evaluable for response; BRAF. B-raf proto-oncogene, serine/threonine kinase; HER2. Human epidermal growth factor receptor 2; BC. Breast cancer; IDH. Isocitrate dehydrogenase; H3K27M. Histone H3 K27 mutation; WHO. World Health Organization; GBM. Glioblastoma; TMZ. Temozolomide; MGMT. O^6^-methylguanine-DNA methyltransferase; rGBM. Recurrent glioblastoma; MB. Medulloblastoma; PCNSL. Primary central nervous system lymphoma; N. Number; NA. Not applicable; T-DXd. Trastuzumab deruxtecan; T-DM1. Trastuzumab emtansine; EGFR-TKI. Epidermal growth factor receptor tyrosine kinase inhibitor; ndHGG. Newly diagnosed high-grade glioma; ndGBM. Newly diagnosed glioblastoma; ABCP. Atezolizumab+bevacizumab+carboplatin+paclitaxel; ACP. Atezolizumab+carboplatin+paclitaxel; BCP. Bevacizumab+carboplatin+paclitaxel; PCI. Prophylactic cranial irradiation; HA-PCI. Hippocampal avoidance-PCI; CSI. Craniospinal irradiation; SDCSI. Standard-dose CSI; LDCSI. Low-dose CSI; IFRT. Involved field radiation therapy; mPFS. Median progression-free survival; OS. Overall survival; *HR*. Hazard ratio; RN. Radionecrosis; TTP. Time to progression; EFS. Event-free survival; AEs. Adverse events; QoL. Quality of life; iPFS. Intracranial PFS; miPFS. Median intracranial progression-free survival; mOS. Median overall survival; *ORR*. Objective response rate; CNS-*ORR*. Central nervous system *ORR*; *iORR*. Intracranial *ORR*; mCNS PFS. Median CNS progression-free survival; HD-MTX. High-dose methotrexate; PFRT. Posterior fossa radiation therapy; 95% CI. 95% confidence interval.

In contrast, treatment strategies for gliomas remain largely focused on radiotherapy and chemotherapy [595], [596]. Immunotherapy has shown limited efficacy in multiple clinical trials, prompting renewed focus on the immunological landscape of gliomas and BrMs, particularly BTME-mediated resistance mechanisms. Notably, the combination of immunotherapy and radiotherapy has demonstrated potential survival benefits [600]. Furthermore, recent phase Ⅲ trials indicate that the personalized DC vaccine DCVax-L can significantly prolong median OS in both ndGBM and rGBM patients **(**Table 1**)** [161], [249], [561], [562], [563], [566], [567], [568], [569], [570], [571], [572], [573], [574], [575], [576], [577], [578], [579], [580], [581], [582], [583], [584], [585], [586], [587], [588], [589], [590], [591], [592], [593], [594], [595], [596], [597], [598], [599], [600], [601], [602], [603].

### New progress in therapy

#### Natural products and drug repurposing

Various naturally derived compounds represent a valuable and expanding source of anti-tumor agents. In particular, several plant- or microorganism-derived products have shown inhibitory effects on glioma or GSCs through distinct mechanisms. For example, the plant extracts such as 7α-acetoxy-6β-hydroxyroyleanone exhibit glioma-inhibitory activity [604], while extracts from the marine fungus *Penicillium* also display anti-tumor potential [595]. In addition, beyond natural products, some antipsychotic drugs have also been reported to exert certain anti-tumor properties [605]. Mechanistically, the flavonoid compound luteolin can effectively inhibit the malignant behaviour of GSCs by blocking the IL-6/STAT3 pathway [606].

#### Immune agonists

Progress has also been made in the development of immunomodulatory strategies, particularly STING agonists. For example, HA-MSA2 conjugates promote immunogenic tumor cell death and immune cell infiltration [607], and ADU-S100 enhances innate immune activation [608]. Consistently, STING activation can reprogram microglial phenotypes [609] and promote antigen presentation following tumor phagocytosis [610]. Furthermore, advances in BBB penetration technologies, including low-intensity pulsed ultrasound [611] and rabies virus glycoprotein (RVG)-modified gold nanospheres [612], significantly improve the targeting efficiency of drugs towards brain tumors.

However, despite these encouraging findings, recent studies suggest that the therapeutic effects of immune agonists remain context-dependent and require further optimization. For example, the STING signaling can also induce high PD-L1 expression in tumor-associated monocytes via the IRF3-IFN-Ⅰ axis, which may limit therapeutic efficacy; notably, TLR2 agonist pretreatment can remodel this pathway and enhance antitumor immunity [613]. Similarly, combinational delivery strategies are being explored to overcome these limitations. Exosome-based delivery systems combining TLR1/2 agonists with cyclic dinucleotides activate APCs and, when combined with ICIs, produce synergistic antitumor effects [614]. In the same vein, Kang *et al*. [615] developed a sono-responsive nanoplatform integrating STING activation with CXCR4 blockade, thereby further enhancing immunotherapeutic efficacy in GBM models.

#### Oncolytic virus therapy

The oncolytic virus therapy exerts anti-tumor effects through selective tumor cell lysis and immune activation [616]. Currently, several genetically modified oncolytic viruses have demonstrated safety and preliminary efficacy in clinical trials. For example, a phase Ⅰ trial of Delta24-RGD demonstrated excellent tolerability [617], while IL-12-expressing oHSV induced immune memory by upregulating tumor MHC-Ⅱ [618]. Notably, oncolytic immune activation by CAN-3110 has shown enhanced anti-tumor effects in rGBM, even in an immunosuppressive microenvironment **(**Table 2**)** [617], [619], [620], [621], [622], [623], [624], [625], [626].

**Table 2:** Clinical trials of oncolytic viruses for brain tumors (2021 to date).

Type	Treatment	Tumor	Sample size (*n*)	Phase	Key results	Adverse events	Identification number	Reference
Oncolytic viruses	CAN-3110	rGBM	41	Ⅰ	mOS: 11.6 months (95% CI 7.8–14.9)	no DLT	NCT03152318	[619]
	G47Δ	Recurrent/progressive GBM patients who have failed previous RT and TMZ	13	Ⅰ/Ⅱ	mOS: 7.3 months (95% CI 6.2–15.2); 1-year survival rate: 38.5%; mPFS: 8 d (95% CI 7–34); Objective response: 1 CR (low-dose group) +1 PR (set dose group); Long-term survivors: 3 patients survived >46 months	AEs rate: 12/13 cases (92.3%); Common related AEs: fever, headache, vomiting (no DLT)	UMIN000002661	[620]
	G47∆ (a triple-mutated, third-generation oncolytic herpes simplex virus type 1)	Recurrent or residual supratentorial GBM patients who have received prior RT and TMZ	19	Ⅱ	1-year survival rate: 84.2% (16/19). mOS: From the start of G47∆ treatment: 20.2 months (95% CI 16.8–23.6); From the start of initial surgery: 28.8 months (95% CI 20.1–37.5)	The most common symptoms were fever (17/19), vomiting, nausea, lymphocytopenia, and leukocytopenia	UMIN000015995	[621]
	NSC-CRAd-S-pk7 (engineered oncolytic adenovirus delivered by neural stem cells)	ndHGG	12	Ⅰ	mPFS: 9.1 months; mOS: 18.4 months	1 case of grade 3 viral meningitis (accidental intraventricular injection) No *DLT* was achieved, and no treatment-related deaths occurred	NCT03072134	[622]
	Ad-TD-nsIL12 (an oncolytic adenovirus expressing nonsecretory interleukin-12)	rHGG	8	Ⅰ	1 case of CR, 1 case of PR; CD4^+^ and CD8^+^ T cell infiltration was observed in the tumor after treatment.	4 patients developed hydrocephalus: 2 were relieved by ventriculoperitoneal shunt, and 2 showed only ventriculomegaly on MRI without neurological deterioration	ChiCTR2000032402	[623]
	Oncolytic measles virus (MV-CEA)	rGBM	22	Ⅰ	mOS: 11.6 months; 1-year survival rate: 45.5% (better than the control group)	No DLT; MTD not reached	NCT00390299	[624]
	Delta24-RGD oncolytic adenovirus	rGBM	19	Ⅰ	4 MRI responses (1 CR survival >8 years)	The main toxicity was a transient increase in intracranial pressure (inflammatory reaction/viral meningitis)	NA	[617]
Adenoviral vector	Ad-hCMV-TK (expressing HSV1-thymidine kinase) Ad-hCMV-Flt3L (expressing Flt3 ligand)	ndHGG	18	Ⅰ	mOS: 21.3 months (95% CI 11.1–26.1)	The MTD was not reached, and there was no DLT. Common grade 3–4 AEs: wound infection (2 cases), thromboembolic events (4 cases)	NCT01811992	[625]
Oncolytic viruses+immunotherapy	Oncolytic virus: intratumoral injection of DNX-2401 ICIs: intravenous injection of pembrolizumab	rGBM	49	Ⅰ/Ⅱ	*ORR*: 10.4% (90% CI 4.2–20.7) did not reach the pre-specified 5% control rate for statistical significance; 12-month OS rate: 52.7% (95% CI 40.1–69.2), significantly higher than the pre-specified 20% control rate; mOS: 12.5 months (95% CI 10.7–13.5); Clinical benefit rate: 56.2% (95% CI 41.1–70.5)	Full-dose combination therapy was well tolerated with no DLT	NCT02798406	[626]

RT. Radiation therapy; TMZ. Temozolomide; PFS. Progression-free survival; OS. Overall survival; AEs. Adverse events; DLT. Dose limiting toxicity; ndHGG. Newly diagnosed high-grade glioma; GBM. Glioblastoma; rGBM. Recurrent GBM; CR. Complete response; PR. Partial response; MTD. Maximum tolerated dose; MRI. Magnetic resonance imaging; HSV1. Herpes simplex virus type 1; ICIs. Immune checkpoint inhibitors; mOS. Median overall survival; 95% CI. 95% confidence interval; mPFS. Median progression-free survival; rHGG. Recurrent high-grade glioma; *ORR*. Objective response rate

To improve the therapeutic efficacy of oncolytic viruses in GBM, current strategies mainly focus on genetic engineering and rational combination approaches that enhance antitumor immunity and remodel the TME. Genetically modified oncolytic viruses can strengthen local immune activation. For example, G47Δ-mIL12 virus reshapes the TME by local IL-12 delivery [627], whereas oAd-CXCL11 virus recruits CD8^+^ T cells and NK cells while reducing immunosuppressive populations such as MDSCs and Tregs [402]. In parallel, combination approaches are being explored to further enhance these effects and overcome resistance-related mechanisms. D2C7-immunotoxin combined with αCD40 antibodies alleviates T cell exhaustion and expands stem-like T cells (NCT04547777) [628]. In addition, IGF2 depletion has been reported to enhance virus-mediated tumor killing [629], further supporting the potential value of mechanism-based combination strategies in improving oncolytic virotherapy.

#### Antibody-drug conjugates

Antibody-drug conjugates (ADCs), which deliver potent cytotoxic agents to tumor cells via specific surface antigen targeting, are being actively explored in both primary and metastatic brain tumors. In GBM, targeting the EGFR has been a major focus. The ADC depatuxizumab mafodotin (ABT-414) demonstrated anti-tumor activity in clinical trials for both ndGBM and rGBM [630], [631], supported by tumor-specific localisation of parental antibody ABT-806 [632]. Beyond EGFR, a phase 0 trial demonstrated that the Trop-2-directed ADC sacituzumab govitecan can accumulate in rGBM, providing a rationale for its further investigation [633].

ADCs have also shown greater promise in BrMs [634]. In patients with HER2-positive breast cancer and leptomeningeal metastasis, a phase Ⅰ/Ⅱ clinical trial reported that intrathecal trastuzumab achieved a disease control rate of 69.2% [635]. In HER2-negative breast cancer with BrMs, the Trop-2-targeting ADC ESG401 demonstrated an intracranial objective response rate of 41% in a phase Ⅰ clinical trial [636]. Similarly, HER3-directed ADC HER3-DXd is also under evaluation in lung and breast cancer BrMs [637], [638]. Additional agents, including ANG1005, a brain-penetrating peptide-drug conjugate with reported activity in leptomeningeal disease, and sacituzumab govitecan, are being assessed in clinical trials for BrMs (NCT06401824). Collectively, the evolving landscape for ADCs in brain tumors underscores the critical importance of target selection, payload properties, and innovative delivery strategies to improve outcomes for patients with brain tumor.

#### Bispecific antibodies

Bispecific antibodies simultaneously target tumor antigens and immune cells, redirecting immune cytotoxicity and enhancing anti-tumor immune responses [639]. Their molecular design also confers favorable BBB penetration, offering distinct advantages for brain tumor immunotherapy [640]. The cluster of differentiation 3 (CD3)/ delta-like ligand 3 (DLL3)-directed T cell engager tarlatamab induces rapid regression of intracranial lesions in treatment-naïve patients with SCLC BrMs, with 90% of patients achieving clinical response or disease stabilization [641]. In addition to their direct immunotherapeutic effects, bispecific antibodies have also been explored as drug delivery tools. A pH-responsive bispecific antibody targeting polyethylene glycol (PEG) and transferrin receptor (TfR) (pH-PEG engager^TfR^) was designed to promote TfR-mediated transcytosis and release PEGylated nanodrugs within acidic endosomes, thereby increasing brain accumulation and improving therapeutic efficacy in GBM [642]. Similarly, glofitamab, a bispecific antibody targeting CD20 and CD3, has been shown to cross the BBB, activate immune cell infiltration in CNS lymphoma, and induce clinical responses [640].

#### Tumor vaccines

Tumor vaccines have also progressed substantially as an immunotherapeutic strategy for brain tumors. For example, personalized neoantigen vaccines have demonstrated favourable safety and durable T cell responses, with significantly prolonged survival in patients mounting multiantigen responses (53 months vs. 27 months) [643]. Consistently, mRNA vaccines demonstrated efficacy in preclinical brain tumor models [644], while a phase Ⅱ trial combining DC vaccines with TLR agonists further confirmed the clinical benefit of vaccine-based immunotherapy [162]. These advancements suggest that vaccine strategies, which integrate precise antigen selection with long-term immune memory induction **(**Table 3**)** [160], [162], [230], [645], [646]. Meanwhile, significant breakthroughs have been achieved in immune activation strategies targeting the postoperative recurrent microenvironment. A charge-reversible, mitochondrial stress-inducing nanoparticle combined with tumor-treating fields disrupts oxidative phosphorylation, remodels the immunosuppressive microenvironment and significantly suppresses postoperative GBM recurrence in mouse models [647]. Additionally, a survivin peptide-CpG oligonucleotide nanovaccine (SPOD-NV) markedly enhances DC activation and antigen presentation. When combined with an anti-CTLA-4 antibody treatment, the combination achieved a 43% complete response rate in the GL261 orthotopic model, offering a novel strategy for brain tumor vaccines [648].

**Table 3:** Clinical trials of tumor vaccines in brain tumors (2021 to date).

Type	Treatment	Tumor	Sample size (*n*)	Phase	Key results	Adverse events	Identification number	References
Tumor vaccines	IDH1(R132H) specific peptide vaccine (IDH1-vac)	Patients with newly diagnosed WHO grade 3–4 IDH1(R132H)^+^ astrocytoma	33	Ⅰ	3-year PFS rate: 63%; 3-year OS rate: 84%; Immune response subgroup: 2-year PFS rate reached 82% (all non-responders progressed within 2 years)	All vaccine-related AEs were grade 1, and no DLTs were observed	NCT02454634	[230]
	Autologous DC vaccine (loaded with allogeneic stem cell-like glioma cell lysate)	ndGBM (*n*=11) rGBM (*n* =25)	36	Ⅰ	ndGBM: mPFS: 8.75 months; mOS: 20.36 months. rGBM: mPFS: 3.23 months (6-month PFS rate 24%); mOS: 11.97 months	Well tolerated	NCT02010606	[645]
	Autologous DC vaccine (AV-GBM-1) incubated with autologous TICs lysate	GBM treated with standard chemoradiotherapy (RT/TMZ) first	57	Ⅱ	mPFS: 10.4 months; mOS: 16.0 months; 2-year OS rate: 27%.	Treatment-emergent AEs (possibly disease-related): seizure (33%), headache (37%), focal neurologic symptoms (28%)	NCT03400917	[160]
Tumor vaccines+β-glucan	GD2/GD3 vaccine+β-glucan	Relapsed high-risk neuroblastoma	102	Ⅱ	5-year survival rate: PFS 32%, OS 71%	Only grade 1–2 local reactions/fever, no grade ≥ 3 AEs	NCT00911560	[646]
Tumor vaccines+TLR agonist	Basic treatment: autologo	Patients with newly diagnosed or recurrent WHO grade Ⅲ–Ⅳ malignant glioma	23	Ⅱ	Enhanced systemic immune response	Well tolerated	NCT01204684	[162]

IDH. Isocitrate dehydrogenase; PFS. Progression-free survival; OS. Overall survival; DLT. Dose limiting toxicity; AEs. Adverse events; DC. Dendritic cell; ndGBM. Newly diagnosed glioblastoma; rGBM. Recurrent glioblastoma; RT. Radiation therapy; TMZ. Temozolomide; ITT. Intent-to-treat; TLR. Toll-like receptors; WHO. World Health Organization; mPFS. Median progression-free survival; mOS. Median overall survival

Future directions should prioritize optimizing the intracranial delivery efficiency of oncolytic viruses, defining optimal sequencing of combination therapies, and improving the accuracy of neoantigen prediction algorithms for vaccines development. Moreover, biomarker-based patient stratification will be essential for personalized treatment. With a deeper understanding of tumor-immune interactions and technological advances, oncolytic virus and immune therapy strategies are expected to become viable treatment options for patients with brain tumor.

#### CAR-T cell therapy

The CAR-T cell therapy has shown promise in malignant brain tumors, but its efficacy is constrained by the immunosuppressive BTME. Activation of the PD-L1/PD-1 pathway inhibits CAR-T cell function, but combining CAR-T cells with anti-PD-1 therapy failed to produce synergistic effects in the lung cancer BrM model [649]. In contrast, B7-H3-targeted CAR-T and CAR-NK cells demonstrated significant anti-tumor activity in GBM xenograft models [650], [651], and a phase Ⅰ clinical trial of B7-H3 CAR-T cells (BrainChild-03) confirmed their preliminary safety and immune activation potential [652]. Dual-targeting strategies have also shown promise. EGFR-IL-13Rα2 CAR-T cells exhibited biological activity in organoid models [653]. In addition, GD2 CAR-T cells induced tumor regression and neurological improvement in patients with diffuse intrinsic pontine glioma [654].

To overcome BTME-mediated resistance, current efforts focus on both CAR engineering and local immunomodulatory strategies [655]. Notch-regulated EGFRvⅢ CAR-T cells reduce T cell exhaustion by continuous antigen stimulation [656]. Another important approach involves modulation of the local immune microenvironment. For example, an implantable and biodegradable system enabling sustained release of small-molecule immunomodulators reprograms myeloid cells, induces IL-12 expression, and enhances CD4^+^ and CD8^+^ T cells infiltration, resulting in durable tumor control in mouse models and validation in human glioma specimens [657]. Similarly, tumor-associated tyrosine kinase with immunoglobulin-like and EGF-like domains 2 (TIE2)^+^ macrophages have been engineered to deliver IFN-α and orthogonal IL-2 (oIL-2), improving B7-H3-targeting CAR-T cell function, limiting early exhaustion, and activating endogenous T cell responses, even in tumors with heterogeneous antigen expression [658]. Additional engineering strategies further improve CAR efficacy through distinct mechanisms. The fibrin-based mechanical signaling can decouple T cell proliferation and differentiation, generating stem-like CAR-T cells with improved efficacy in brain tumor [659]. In parallel, multiantigen targeting approaches, such as CD44/CD133 dual-targeted IL7Rα-overexpressing CAR-T cells [660] and PTPRZ1-targeted CAR-T cells [661], have increased cytotoxicity through multiantigen coverage and bystander effects. Notably, EphA3 CAR-T cells eliminate orthotopic GBM and induce long-term immune memory that prevents tumor recurrence [257], [662]. Additionally, the introduction of cell type-specific signaling domains, such as the neutrophil γ domain [663], and CAR-NK cell-derived exosome nanobombs [492] demonstrates the innovative potential of cross-cellular engineering strategies **(**Table 4**)** [654], [664], [665], [666], [667], [668].

**Table 4:** Clinical trials of CAR-T therapy for brain tumors (2021 to date).

Type	Treatment	Tumor	Sample size (*n*)	Phase	Key results	Adverse events	Identification number	References
CAR-T	Dual-target CAR-T cells (CART-EGFR-IL-13Rα2)	multifocal rGBM	6	Ⅰ	Early MRI showed that the enhancement lesions of all 6 patients had shrunk, but the *ORR* had not yet reached	All patients developed early neurotoxicity (ICANS); 1 case of DLT (grade 3 anorexia, general weakness) was controlled with high-dose dexamethasone+anakinra (anti-IL-1R)	NCT05168423	[664]
	IL-13Rα2-targeted CAR-T cell therapy	rHGG (primarily rGBM)	65	Ⅰ	DCR: 50% (29/58) of patients achieved stable disease or better; mOS: all rGBM patients 7.7 months	The main grade 3 *AEs* were encephalopathy (1 case) and ataxia (1 case); Generally well tolerated	NCT02208362	[665]
	B7-H3 targeted CAR-T cells	Children and young adults with recurrent/refractory diffuse intrinsic pontine glioma	21	Ⅰ	Median survival: 10.7 months from first CAR-T infusion; 19.8 months from diagnosis; Three long-term survivors	Common: headache, fatigue, fever; DLT: 1 case of intratumoral hemorrhage	NCT04185038	[666]
	IV: single autologous GD2-CART infusion; ICV: ICV GD2-CART	H3K27M mutation DMG patients	11	Ⅰ	Tumor volume changes: 4 cases had a significant reduction (52%, 54%, 91%, and 100%; 3 cases had a slight reduction, 1 case had CR lasting >30 months. Neurological function improvement: 9 patients showed improvement in clinical scores	GD2-CART (IV+ICV) was controllable in DMG	NCT04196413	[654]
CAR-T+recombinant human IL-2	Allogeneic IL-13Rα2-targeted CAR-T cells (GRm13Z40-2) +recombinant human IL-2	Unresectable rGBM	6	Ⅰ	Transient tumor shrinkage and/or tumor necrosis were observed at the site of T cell injection in 4 of 6 patients; GRm13Z40-2 cells showed dexamethasone-resistant effector activity *in vitro* and were non-alloreactive	Well tolerated, with no serious AEs reported	NCT01082926	[667]
CAR-T+immunotherapy	EGFRvⅢ-targeted CAR-T cell therapy+Pembrolizumab (anti-PD-1)	EGFRvⅢ-positive ndGBM	7	Ⅰ	mPFS: 5.2 months (90% CI 2.9–6.0); mOS: 11.8 months (90% CI 9.2–14.2)	Good safety	NCT03726515	[668]

CAR-T. Chimeric antigen receptor T; rGBM. Recurrent glioblastoma; *ORR*. Objective response rate; EGFR. Epidermal growth factor receptor; IL-13Rα2. Interleukin-13 receptor alpha 2; MRI. Magnetic resonance imaging; TLR. Toll-like receptors; poly-ICLC. Polyinosinic-polycytidylic acid stabilized with poly-L-lysine and carboxymethylcellulose; ICANS. Immune effector cell-associated neurotoxicity syndrome; rHGG. Recurrent high-grade glioma; OS. Overall survival; B7-H3. B7 homologue 3; DLT. Dose limiting toxicity; IV. Intravenous infusion; ICV. Intra-cerebroventricular; H3K27M. Histone H3 lysine 27 to methionine mutation; DMG. Diffuse midline glioma; IL. Interleukin; PFS. Progression-free survival; PD-1. Programmed death 1; ndGBM. Newly diagnosed glioblastoma; CR. Complete remission; DCR. Disease control rate; AEs. Adverse events; mPFS. Median progression-free survival; mOS. Median overall survival.

Monotherapy with CAR-T cells is not always sufficient to overcome the heterogeneity and therapeutic resistance in brain tumors, making combination strategies necessary. FLASH radiotherapy reprograms lipid metabolism and triggers macrophage polarization [669], while TGF-β dual-targeted CAR-T cells synergistically reduce infiltration of immunosuppressive myeloid cells [401]. These findings highlight that future optimization should integrate local delivery, metabolic microenvironment remodeling, and precise targeting of immunosuppressive cell subsets [287], [422].

Despite promising outcomes from early-phase clinical trials, such as GD2 CAR-T cell therapy for high-risk medulloblastoma [670], major challenges remain. These include antigen heterogeneity and immune escape, infiltration barriers in solid tumors, and CNS-specific toxicity [671]. Potential solutions may include dynamic biomarker monitoring [652], logic-gated CAR designs, and integration with BBB modulation techniques [672]. The development of spatial multiomics and organoid models may render individualized CAR-T cell therapy feasible, necessitating a meticulous equilibrium between efficacy and safety.

### Combination therapy strategies

Combination therapies have emerged as a pivotal approach to overcome BTME-driven therapeutic resistance [673], [674]. Anti-angiogenic agents promote vascular normalization and enhance T cell infiltration, thereby showing strong synergy with ICIs [675]. Deletion of FcγRⅡB may serve as a combinational immunomodulatory strategy, as it enhances the stem-like properties of CD8^+^ T cells and prolongs antitumor activity in GBM [676]. Local delivery of CAR-NK cells combined with systemic PD-1 blockade significantly increases T and NKT cells infiltration [677], while CXCL11-armed oncolytic adenoviruses increase the infiltration and function of CAR-T cells in the immunosuppressive microenvironment [402]. In addition, CDK4/6 inhibitors enhance the selective replication and DNA damage effects of the oncolytic virus VSVΔ51 by promoting the autophagic degradation of mitochondrial antiviral signaling protein (MAVS) [678], offering new immune-targeted combination strategies.

Moreover, the combination of radiotherapy and immunotherapy has demonstrated distinct benefits. Radiotherapy-induced release of tumor-derived microvesicles activates chemokine signaling pathways, such as CCL5 and CXCL2, promoting T cell recruitment and improving responses to subsequent PD-1 blockade [679]. Combined radiotherapy with chemotherapy can also induce immunogenic cell death, releasing tumor antigens and damage-associated molecular patterns (DAMPs), which disrupts immune tolerance [674]. Chemotherapeutic agents such as TMZ may enhance the efficacy of immunotherapy, thereby providing a rationale for multimodal combination strategies [680].

Metabolic regulation has become a new direction for combination therapies. Targeting lactate metabolism sensitizes GBM cells to radiotherapy [410], while radiation-induced ferroptosis can be enhanced by inhibiting lactate-dependent antioxidant defences [681], [682]. Chemical dynamics therapy (CDT) induces tumor cell death through the Fenton reaction, while α-cyano-4-hydroxycinnamic acid (CHC) nanoparticles enhance CDT efficacy by inhibiting MCT4 and modulating microenvironment pH [683]. Upregulating the key glutamine transporter ASCT2 activates SREBP-1 via ammonia release, whereas combined targeting of glutamine and lysosome-dependent lipid metabolism effectively inhibits GBM [684].

Overall, multitarget combination strategies offer promising avenues to overcome brain tumors. Future efforts should focus on optimizing treatment sequencing, developing dynamic BTME monitoring technologies and establishing personalized decision systems. Integration of multiomics data with artificial intelligence (AI)-based predictive models may facilitate a shift from empirical combinations to precise designs [685].

### Nanotechnology and drug delivery systems

Recent advances in BBB-penetrating technologies have improved intracranial delivery efficiency through diverse targeting strategies. TfR-mediated nanoparticles exploit high endothelial TfR expression for targeted transport in the brain [686]. A glutathione-responsive TMZ nanocapsule (ApoE-MT/siPKM2 NC) enables co-delivery of chemotherapy drugs and gene therapy agents for glioma [687]. The cRGD/PSDOX-Cur@NP system enhances BBB penetration and reduces doxorubicin cardiotoxicity [688]. In glioma, biomimetic nanoplatforms have further expanded therapeutic applications. For example, L-D-I/NPs targeting LRP1 receptor to induce ferroptosis [689], while tumor cell membrane-wrapped gold nanorods (GBM-PDTCM/AuNRs) have been developed for surgical navigation [690]. Nanotechnology-based immunotherapy strategies have also rapidly evolved. A wireless charging mitochondria-targeted nanoantenna (WINA) disrupts mitochondrial membrane potential, releases DAMPs, and enhances DC activation and T cell infiltration [691]. Similarly, an implantable catalytic therapy and antigen capture scaffold (CAS) based on iron-containing metal-organic frameworks (MOFs) continuously generates reactive oxygen species and enhances postoperative immunotherapy [692].

Responsive nanocarriers enable microenvironment-triggered drug release. The temperature and pH-responsive in situ hydrogels of gelatin [686] and GSH-responsive crosslinker systems achieve condition-dependent activation of BTME [687]. Surface modifications, including poly-L-glutamic acid/hyaluronic acid [446] and layer-by-layer polymer functionalization [693], significantly enhance tumor targeting. Manganese-based nanocomposites [694] and imine bond hydrolysis systems [695] achieve precise drug release and immune activation. Among responsive nanosystems, a near-infrared-Ⅱ (NIR-Ⅱ) responsive cell-membrane-disruptive CuS nanosheet supports deep-tissue photothermal therapy while serving as an antigen reservoir to sustain DC activation [695]. Additionally, RVG-modified gold yarn spheres (RVG@GY) promote drug penetration and T cell infiltration under magnetoelectric irradiation, suppressing tumor recurrence [612].

Nanotechnology also enables multifunctional therapeutic design in brain tumors. For example, camouflaged nanotransducers can induce the M1 polarization of microglia through electrical signals [696], while CuS nanosheets have simultaneous photothermal and immune-activating effects [695]. In addition, albumin-stabilized manganese-based nanocomposites [694] and azelaic acid sustained-release systems [697] improve controlled drug release, further broadening the utility of nanoplatforms. Notably, Bradykinin-based near-infrared-responsive nanoparticles (BK@AIE NPs) activate immune effectors such as NK cells and CD8^+^ T cells in deep tumors [698]. Dual-layer microneedle patches [699] and the lipid nanoparticle-mRNA formulations and dendritic cell therapy combination strategy [700] represent innovative combinations of local delivery and systemic immune modulation. Dual catalytic oxide nanosponges and pH-responsive catalytic nanoplatforms further enhance immune reprogramming and synergize with PD-1 blockade in metastatic models [701], [702].

Despite extensive preclinical development, the clinical translation of nanotherapeutic platforms remains limited overall. Nevertheless, early clinical studies have provided preliminary support for their translational potential. For example, the phase Ⅰ NANO-RAD clinical trial confirmed the clinical benefit of AGuIX nanoparticles combined with WBRT, with improvement observed in most treated patients [703]. Nanoparticles targeting CD47/PD-L1 and dendritic polymer-manganese delivery systems further enhance radiotherapy and chemotherapy efficacy [704], [705]. Recent efforts increasingly emphasize an integrated “diagnosis-treatment-monitoring” approach. Future research should focus on dynamic and reversible BBB opening, multiparameter responsive delivery systems, and real-time monitoring of BTME. With advances in materials science, bioengineering, and AI, nanotherapy platforms are expected to transition from the laboratory to clinical applications.

### Early-stage clinical trial

Although the overall clinical translation of many approaches remains limited, early-phase clinical trials continue to expand, particularly in lung cancer BrMs. To illustrate this, we summarize the current landscape of phase Ⅰ–Ⅱ clinical trials involving brain tumors. In addition to the development of novel agents and strategies to overcome resistance to targeted therapies, combination strategies integrating targeted therapy with radiotherapy have shown encouraging efficacy [706], [707], [708], [709], [710]. Advances in radiotherapy techniques have also contributed to this evolving landscape, including prophylactic cranial irradiation (PCI), hippocampal avoidance HA-WBRT, preoperative stereotactic radiosurgery (SRS), and proton craniospinal irradiation [711], [712], [713], [714]. In patients without an oncogenic driver, the combination of chemotherapy and immunotherapy remains the standard treatment approach [715], [716]. In this setting, combining immunotherapy with radiotherapy has also shown clinical benefit [250].

In breast cancer BrMs, recent progress has focused predominantly on HER2-positive subtypes. For example, the ADC trastuzumab deruxtecan has shown activity in leptomeningeal metastases from breast cancer [635], while tyrosine kinase inhibitors such as lapatinib and pyrotinib have shown preliminary efficacy when combined with chemotherapy or radiotherapy [717], [718], [719]. In melanoma BrMs, both targeted therapies and dual ICIs achieved encouraging intracranial response rates of 70.8% and 51%, respectively **(**Table 5**)** [250], [635], [636], [703], [706], [707], [708], [709], [710], [711], [712], [713], [714], [715], [716], [717], [718], [719], [720], [721], [722], [723], [724], [725], [726], [727], [728], [729], [730], [731], [732], [733], [734], [735], [736], [737].

**Table 5:** Phase Ⅰ–Ⅱ clinical trials for BrMs (2021 to date).

Therapy	Treatment	Tumor	Sample size (*n*)	Phase	Key results	AEs	Identification number	References
LCBM								
Targeted therapy	Selpercatinib	RET fusion-positive NSCLC BrM at baseline	80	Ⅰ/Ⅱ	*iORR*: 82% (CR rate 23%); Median duration of response: not reached (55% ongoing response at 9.3 months)	No new safety signals	NCT03157128	[722]
	Lazertinib	NSCLC BrM after failure of first-generation/second-generation EGFR-TKI treatment	40	Ⅱ	*iORR*: 55%; T790M-positive patients, *iORR*: 80% (5 cases); T790M-negative patients, *iORR*: 43% (21 cases); T790M-unknown patients, *iORR*: 67% (12 cases); miPFS: 15.8 months	Most AEs were grade 1–2	NCT05326425	[706]
	Dacomitinib	Patients with *EGFR*-mutated advanced NSCLC and untreated BrM (≥5 mm)	30	Ⅱ	*iORR*: 96.7% (29/30), of which 63.3% achieved *CR*; iPFS: 12-month rate 78.6% (95% CI 64.8–95.4); 18-month rate 70.4% (95% CI 54.9–90.1)	The incidence of grade ≥3 AEs was 16.7%; 83.3% of patients required dose reduction (no permanent discontinuation)	NCT04339829	[707]
	Osimertinib	*EGFR*-mutated NSCLC LM (*n*=73) previously received first-generation/second-generation EGFR-TKI treatment	73	Ⅱ	OS: 15.6 mouths (95% CI 11.5–20.2); LM-specific mOS: 15.0 months (95% CI 11.3–18.7); *ORR*: 51.6% (including 15.6% CR); DCR: 81.3%; mLM-PFS: 11.2 months (95% CI 7.7–15.3); Median duration of response: 12.6 months (95% CI 7.6–17.7)	Major AEs: grade 1–2	NCT04563871	[708]
	Osimertinib	Patients with NSCLC BrM with *EGFR*-sensitive mutations (T790M-positive) who did not receive RT	66	Ⅱ	PFS related to BrM: 25.2 months (exon 19 deletion) vs. 8.3 months (L858R)	NA	OCEAN	[723]
Targeted therapy+RT	Trametinib+WBRT	BrM	10	Ⅰ	mOS: 2.2 months	DLT: 2 cases occurred at DL2 (1.5 mg) (grade 4 thrombocytopenia and grade 3 diarrhea); MTD: <1.5 mg (1.0 mg is safe); Other toxicities: grade 3–4 AEs occurred in 4 out of 6 patients at DL2 (a total of 12/13 cases)	NCT02015117	[709]
	WBRT+anlotinib	Advanced NSCLC and multiple BrM (>3)	28	Ⅱ	miPFS: 11.1 months (95% CI 5.4–16.8); mOS: 13.4 months (95% CI 5.2–21.6); *iORR*: 71.4% (6 CR+14 PR)	Common: hypertension (71.4%), fatigue (64.3%), anorexia (46.4%), hand-foot skin reaction (25.0%); no ≥grade 4 AEs, no intracranial hemorrhage	ChiCTR 1900022093	[710]
Targeted therapy+TMZ	Olaparib+ TMZ	Patients with relapsed SCLC	15	Ⅰ/Ⅱ	CNS efficacy (*n*=15): CR: 6 cases (40%); PR: 4 cases (26.7%); SD: 3 cases (20%); CNS DCR: 87% (95% CI 59.5–98.3)	No AEs that met DLT	NCT02446704	[724]
	V (SHH inhibitor) + TMZ vs. TMZ alone	Relapsed/refractory SHH-type adult medulloblastoma: Group A (V+TMZ, *n* =10), Group B (TMZ alone, *n*=5), Group C (TMZ-treated patients treated with V alone, *n*=9)	24	Ⅰ/Ⅱ	Group A: PFS-6 20% (did not reach the trial continuation threshold); Group B: PFS-6 not reported, *ORR* 20%; Group C: PFS-6 37.5%, *ORR* 22.2%	No increased toxicity in the combination group	NCT05166005	[725]
RT	SRS	1–10 untreated BrM [primary cancer not limited (SPACE group *n*=99; MPRAGE group *n*=103)]	202	Ⅱ	12-month WBRT-free survival rate: Overall: 77.1% (95% CI 69.5–83.1); SPACE group: 78.5% (95% CI 66.7–86.5); MPRAGE group: 76.0% (95% CI 65.2–83.9) (*HR*=0.84, *P=*0.590); Patients with 5-10 BrMs: WBRT-free survival was shorter (*HR*=3.13, *P=*0.002); mOS: 13.1 months; SPACE group: 10.5 months; MPRAGE group: 15.2 months (*HR*=1.10, *P=*0.585)	Neurological mortality: 10.1%	NCT03303365	[726]
	Preoperative SRS	>2 cm BrM (primary cancer is not limited)	35	Ⅰ	Local control rate: 6 months: 85.9%; 12 months: 76.6% (median follow-up 64 months); LM: 2-year incidence 0%	DLT: > 3–4 cm group: 2 DLTs occurred at 21 Gy (MTD was 18 Gy); > 4–6 cm group: MTD was 18 Gy; ≥ Grade 3 AEs: 1 case of grade 3 radiation necrosis (occurred at 5 months)	NCT01891318	[727]
	Preoperative SRS	BrM	26	Ⅱ	OS: 66%; Distant brain recurrence rate: 37.3% LM rate: 6%; Local recurrence rate: 0%	NA	NCT04895592	[728]
	Preoperative SRS	BrM	26	Ⅱ	1-year LTC rate: 77.2% before surgery vs. 82.5% after surgery (*P=*0.61); Composite endpoint: 68.3% vs. 72.7% (*P*=0.38); mOS: 17.0 months vs. 14.0 months (*P*=0.61)	LM incidence: 3.8% before surgery vs. 16.7% after surgery (*P*=0.20)	NCT02514915	[713]
	HA-WBRT	BrM	65	Ⅱ	At 1 month, the HVLT-R recognition discrimination index (*P*=0.019) and total memory score (*P*=0.020) were significantly better in the HA-WBRT group; No difference in PFS or OS	NA	NCT02393131	[711]
	PCI	High-risk NSCLC patients	84	Ⅱ	Cumulative incidence of BrM at 24 months: 7% in the PCI group vs. 38% in the control group (*HR*=0.12, *P* < 0.001); mOS: 64.5 months vs. 19.8 months (*HR*=0.41, *P*=0.007); PFS was significantly prolonged	NA	NCT01603849	[712]
	Proton CSI	Patients with LM from solid tumors	24	Ⅰ	mCNS-PFS: 7 months (95% CI 5–13); mOS: 8 months (95% CI 6–NR); 19% of patients had CNS-PFS >12 months	2 cases of DLT occurred in the dose expansion group; All DLTs resolved spontaneously	NCT03520504	[714]
RT+other	AGuIX nanoparticles+WBRT	Multiple BrM	15	Ⅰ	Thirteen of the four evaluable patients (93%) achieved clinical benefit	No DLT (up to 100 mg/kg)	NANO-RAD	[703]
	Nitroglycerin+WBRT	The proportion of *EGFR* mutations in patients with NSCLC BrM: control group 52% (26/50); NTG group 45.7% (21/46)	96	Ⅱ	NTG group vs. control group *iORR*: 56.5% vs. 32.7% (*RR*=1.73, *P=*0.024); *iPFS* (overall): 27.7 weeks vs. 9.6 weeks (*HR*=0.5, *P=*0.02)	The incidence of vomiting was higher in the NTG group (*P=*0.016)	NCT04338867	[729]
Chemotherapy	Intrathecal pemetrexed	Patients with NSCLC LM after failure of EGFR-TKI therapy	132	Ⅱ	mOS: 12 months (95% CI 10.4–13.6); RANO-assessed response rate: 80.3% (106/132)	Myelosuppression (31.8%, 42 cases), reversible with symptomatic treatment.	ChiCTR1800016615	[715]
Chemotherapy+anti-angiogenesis therapy	Ramucirumab+docetaxel	Advanced NSCLC with asymptomatic measurable BrM that progressed after chemotherapy	25	Ⅱ	iPFS: 4.6 months (95% CI 2.5–5.9); mOS: 20.9 months (95% CI 6.6–NE); *ORR*: 20% (95% CI 6.8–40.7); *DCR*: 68% (95% CI 46.5–85.1)	Grade 3 or above: 40% neutropenia; No intracranial hemorrhage or grade 5 AEs	jRCTs071180048	[730]
Immunotherapy+RT	SRS+nivolumab/ipilimumab	Patients with active NSCLC BrM who are suitable for SRS	13	Ⅰ/Ⅱ	4-month iPFS rate: 70.7%	DLT incidence: 10% (1/10); ≥ Grade 3 treatment-related AEs: abnormal liver function, fatigue/nausea, adrenal insufficiency, and myocarditis	NCT02696993	[731]
Immunotherapy+chemotherapy	Induction phase: Atezolizumab+carboplatin +pemetrexed (4–6 cycles) Maintenance phase: Atezolizumab +pemetrexed (up to 2 years)	Advanced non-squamous NSCLC with untreated BrM	40	Ⅱ	PFS-12: 62.2% (95% CI 47.1–76.2); miPFS: 6.9 months; Intracranial response rate: 42.7% (95% CI 28.1–57.9); Systemic mPFS: 8.9 months; Systemic response rate: 45% (95% CI 28.1–57.9); mOS: 11.8 months (95% CI 7.6–16.9); 2-year OS rate: 27.5% (95% CI 16.6–45.5)	Grade ≥3 AEs in the first 9 weeks: 27.5%; Grade 3-4 neurological events: 12.5% (5 cases)	NCT03526900	[732]
Immunotherapy+chemotherapy	Camrelizumab+pemetrexed+carboplatin	Patients with newly diagnosed non-squamous NSCLC and BrM	45	Ⅱ	*iORR*: 52.5%; miPFS: 7.6 months	≥ Grade 3 AEs: neutropenia (13.3%), anemia (8.9%); 1 treatment-related death (immune pneumonia)	NCT04211090	[716]
Immunotherapy+RT +chemotherapy	RT (SRS/WBRT)+camrelizumab (PD-1 inhibitor)+platinum	Patients with advanced NSCLC BrM without driver gene mutations (EGFR/ALK/ROS1 negative)	65	Ⅱ	PFS-6: 71.7% (95% CI 58.9–81.1)	Common grade 3–4 AEs: neutropenia (22%), leukopenia (15%), thrombocytopenia (15%), and lymphocytopenia (14%)	NCT04291092	[250]
BCBM								
ADC	ESG401 (ADC drug targeting TROP2, carrying topoisomerase Ⅰ inhibitor SN-38)	HER2-negative BC BrM	17	Ⅰ	*iORR*: 41% (7/17, including 3 CR); *iDCR*: 76%; Systemic *ORR*: 53%; mPFS: 5.7 months	NA	NCT04892342	[636]
	Trastuzumab	HER2^+^ BCLM	34	Ⅰ/Ⅱ	*ORR*: 19.2% (5 PR); DCR: 69.2% (including stable disease); mOS: Overall: 8.3 months (95% CI 5.2–19.6); BC subgroup: 10.5 months (95% CI 5.2–20.9)	The only dose-limiting toxicity was grade 4 arachnoiditis (80 mg group); no CSF drug accumulation toxicity	NCT01325207	[635]
Immunotherapy	Pembrolizumab	Patients with BrM from multiple tumor types, including BC, melanoma, and sarcoma;Cohort A: untreated BrMs (*n*=9); Cohort B: recurrent/progressive BrMs (*n=*48)	57	Ⅱ	Intracranial benefit rate: 42.1% (90% CI 31–54); mOS (overall): 8.0 months (90% CI 5.5–8.7); Cohort A: 6.5 months (90% CI 4.5–18.7); Cohort B: 8.1 months (90% CI 5.3–9.6); Long-term survivors: 7 patients (12.3%) with OS>2 years	Grade ≥3 AEs: 52% (95% CI 41–64); Grade 4 AEs: 2 (cerebral edema, possibly treatment-related)	NCT02886585	[733]
Immunotherapy+RT	Nivolumab+SRS	BrM from multiple subtypes of BC	12	Ⅰb	Local control rate: 94% at 12 months	No radiation necrosis occurred	NCT03807765	[734]
RT	Experimental group: proton craniospinal radiotherapy (pCSI) Control group: photon conformal field radiotherapy (IFRT)	NSCLC and BC with LM were randomized to pCSI (*n =* 42) vs. IFRT (*n =* 21)	63	Ⅱ	pCSI group vs. IFRT group: mCNS PFS: 7.5 months (95% CI 6.6–NR) vs. 2.3 months (95% CI 1.2–5.8); mOS: 9.9 months (95% CI 7.5–NR) vs. 6.0 months (95% CI 3.9–NR).	No significant difference in grade 3–4 AEs (*P*=0.19)	NCT04343573	[735]
Targeted therapy+RT	Lapatinib+WBRT or SRS	HER2^+^ BC BrM	116	Ⅱ	Combination group vs. RT group 12-week intracranial CR: 0% vs. 6% (*P*=0.97); 4-week *ORR*: 55% vs. 42%	Grade 3/4 *AEs* were more common in the combination group (34% vs. 8%)	NCT01622868	[717]
Targeted therapy+RT+chemotherapy	RT+pyrotinib+capecitabine	ERBB2^+^ BC BrM	40	Ⅱ	1-year CNS PFS rate: 74.9% (95% CI 61.9–90.7); mCNS PFS: 18.0 months (95% CI 15.5–NR); 1-year PFS rate: 66.9% (95% CI 53.1–84.2); mPFS: 17.6 months (95% CI 12.8–34.1); CNS *ORR*: 85% (34/40); mOS: NR	Common grade 3–4 AEs: diarrhea (7.5%); Special AEs: asymptomatic radiation necrosis (6.0%, 4/67 lesions); Overall safety: acceptable	NCT04582968	[718]
Targeted therapy + chemotherapy	Pyrotinib+capecitabine	HER2^+^ BC BrM Cohort A: no RT (*n =* 59); Cohort B: progression after RT (*n* = 19)	78	Ⅱ	*ORR*: Cohort A: 74.6% (44/59); Cohort B: 42.1% (8/19)	No treatment-related deaths occurred. Major grade ≥3 AEs: diarrhea (24% in cohort A, 21% in cohort B)	NCT03691051	[719]
MBM								
Targeted therapy	Encorafenib+binimetinib sequential RT	*BRAFV600*-mutant MBM	48	Ⅱ	Intracranial response rate: 70.8% (95% CI 55.9–83.1), including CR: 10.4%; miPFS: 8.5 months (95% CI 6.4–11.8); mOS: 15.9 months (95% CI 10.7–21.4); Patients receiving RT: miPFS 8.3 months, OS 13.9 months	The most common grade 3–4 AEs: ALT elevation (10.4%); Well tolerated, with a low incidence of high-grade AEs	E-BRAIN/GEM1802	[720]
Dual-immunotherapy	Nivolumab+ipilimumab	MBM Cohort A (asymptomatic): ECOG 0–1, no neurological symptoms and corticosteroids (*n=*101); Cohort B (symptomatic): ECOG 0-2, stable neurological symptoms, acceptable low-dose dexamethasone (*n=*8)	119	Ⅱ	Intracranial clinical benefit rate: Cohort A: 57.4% (58/101); Cohort B: 16.7% (3/18). *ORR*: Cohort A: 53.5% (54/101); Cohort B: 16.7% (3/18)	Cohort A: Common grade 3–4 AEs: ALT/AST increase (15%), colitis, diarrhea, and hypophysitis (5% each); Cohort B: no grade 4 AEs	NCT02320058	[736]
	Ipilimumab+nivolumab vs. Nivolumab alone	Asymptomatic patients with MBM who have not received immunotherapy: Cohort A: dual immunotherapy (*n=*36); Cohort B: nivolumab alone (*n=*25); Cohort C: non-randomized, nivolumab alone (*n=*15)	76	Ⅱ	Intracranial response rate: Cohort A 51% (18/36), Cohort B 20% (5/25), Cohort C 6% (1/15); PFS: Cohort A 42%, Cohort B 15%, Cohort C 6%; OS: Cohort A 48%, Cohort B 26%, Cohort C 13%	No new toxicities reported	NCT02374242	[721]
Immunotherapy+ anti-angiogenesis therapy	Induction phase: bevacizumab+pembrolizumab; Maintenance phase: pembrolizumab alone (up to 2 years)	MBM patients who have not received PD-L1 inhibitor treatment	37	Ⅱ	BrM-ORR: 54.1% (95% CI 36.9–70.5); Extracranial *ORR*: 56.3% (95% CI 37.7–73.6); miPFS: 2.2 years (95% CI 0.41–NR); mOS: 4.3 years (95% CI 1.6–NR); 4-year OS rate: 51.6%	Bevacizumab-related grade 3 AEs: 10.8% Pembrolizumab-related grade 3 AEs: 18.9%	NCT02681549	[737]

BrM. Brain metastases; NSCLC. Non-small cell lung cancer; *ORR*. Objective response rate; EGFR-TKI. Epidermal growth factor receptor-tyrosine kinase inhibitor; T790M. Thr790Met; AEs. Adverse events; PFS. Progression-free survival; CI. Confidence interval; LM. Leptomeningeal metastasis; OS. Overall survival; CR. Complete response; DCR. Disease control rate; RT. Radiation therapy; WBRT. Whole brain radiotherapy; DLT. Dose limiting toxicity; MTD. Maximum tolerated dose; PR. Partial response; SD. Stable disease; CNS. Central nervous system; TMZ. Temozolomide; SCLC. Small cell lung cancer; SHH. Sonic hedgehog signaling molecule; SRS. Stereotactic radiosurgery; *HR*. Hazard ratio; LTC. Local tumor control; NA. Not applicable; HA-WBRT. Hippocampal avoidance WBRT; PCI. Prophylactic cranial irradiation; CSI. Craniospinal irradiation; NTG. Nitroglycerin; PD-1. Programmed death 1; ALK. Anaplastic lymphoma kinase; ROS1. ROS proto-oncogene 1, receptor tyrosine kinase; ADC. Antibody-drug conjugates; TROP2. Trophoblast cell surface antigen 2; HER2. Human epidermal growth factor receptor 2; BC. Breast cancer; BCLM. BC leptomeningeal metastases; CSF. Cerebrospinal fluid; IFRT. Involved field radiation therapy; ERBB2. Erythroblastic oncogene B2; BRAF. B-raf proto-oncogene, serine/threonine kinase; MBM. Melanoma brain metastasis; ALT. Alanine aminotransferase; AST. Aspartate aminotransferase; iDCR. Intracranial DCR; *iORR*. Intracranial objective response rate; V. Vismodegib; LCBM. Lung cancer brain metastases; RET. RET proto-oncogene, receptor tyrosine kinase; miPFS. Median intracranial progression-free survival; iPFS. Intracranial progression-free survival; mOS. Median overall survival; mLM-PFS. Median leptomeningeal metastasis progression-free survival; DL2. Dose level 2; PFS-6. 6-month progression-free survival; SPACE. Sampling perfection with application-optimized contrasts using different flip-angle evolutions; MPRAGE. Magnetization-prepared rapid gradient-echo; HVLT-R. Hopkins Verbal Learning Test-Revised; NR. Not reached; *RR*. Relative risk; NE. Not possible to estimate; PFS-12. 12-month progression-free survival; EAS. Efficacy analysis set; BCBM. Breast cancer brain metastases; MBM. Melanoma brain metastases; pCSI. Proton craniospinal irradiation; IFRT. Involved-field radiotherapy

In gliomas, targeted therapies against IDH mutations, BET proteins, Sp1, 2-hydroxyoleic acid (2-OHOA), exportin-1 (XPO1), BRAF, and poly ADP-ribose polymerase (PARP) have shown variable efficacy in early-phase studies [738], [739], [740], [741], [742], [743], [744]. Recent research has been increasingly focusing on combination strategies that integrate these targeted agents with standard chemoradiotherapy or antiangiogenic therapies [745], [746], [747]. In parallel, radiotherapy research continues to refine dose fractionation, explore alternative radiation modalities, and assess the role of reirradiation in recurrent disease [748], [749], [750], [751].

Although immunotherapy has reshaped treatment paradigms for lung cancer BrMs, its clinical impacts in gliomas remain constrained. Neoadjuvant pembrolizumab administered before surgery has demonstrated survival benefits [752]. In addition, the combination of ICIs, such as ipilimumab combined with nivolumab, is being actively explored to enhance therapeutic efficacy [753], [754]. Current studies aim to evaluate the synergy between immunotherapy and radiotherapy, as well as their combination with standard-of-care treatments **(**Table 6**)** [633], [738], [739], [740], [741], [742], [743], [744], [745], [746], [747], [748], [749], [750], [751], [752], [753], [754], [755], [756], [757], [758], [759], [760], [761], [762], [763], [764], [765], [766], [767], [768], [769], [770], [771], [772], [773], [774], [775], [776], [777], [778], [779], [780], [781], [782], [783], [784], [785], [786]. These combinatorial approaches represent a major direction in glioma immunotherapy.

**Table 6:** Phase Ⅰ–Ⅱ clinical trials for glioma (2021 to date).

Type	Treatment	Tumor	Sample size (*n*)	Phase	Key results	Adverse events	Identification number	References
Targeted therapy	Letrozole (aromatase inhibitor)	rHGG	21	0/Ⅰ	RP2D was determined to be 15 mg/d	Common adverse reactions: fatigue, nausea, musculoskeletal discomfort, anxiety, and depression; no DLT was observed	NCT03122197	[755]
	DS-1001 (*IDH1* mutation-selective inhibitor)	Recurrent/Progressive *IDH1*-mutated glioma	47	Ⅰ	mPFS: 10.4 months for enhancing lesions, not reached (> 24.1 months) for non-enhancing lesions; *ORR*: 17.1% for enhancing lesions, 33.3% for non-enhancing lesions	MTD was not reached. Grade 3 AEs: 42.6%; No grade 4/5	NCT04458272	[738]
	hrBMP4	rGBM	15	Ⅰ	Tumor growth in the hrBMP4-infiltrated area was inhibited; the survival of the two patients with complete remission was significantly prolonged (>30 months)	MTD/DLT was not reached, and there were no drug-related serious AEs	NCT02869243	[756]
	BET inhibitor Trotabresib	rHGG who are scheduled for salvage surgery	20	Ⅰ	6-month PFS rate: 12%;Two patients had stable disease	Grade 3/4 thrombocytopenia (5/16 patients on maintenance therapy); No surgery was delayed/cancelled	NCT04047303	[739]
	Vorasidenib	Solid tumors with *IDH1/IDH2* mutations (*n*=93): glioma subgroup (*n=*52, relapsed/progressed after previous standard treatment)	52	Ⅰ	Non-enhancing glioma: *ORR* 18% (1 PR+3 minor responses); mPFS: 36.8 months (95% CI 11.2–40.8); Enhancing glioma: mPFS: 3.6 months (95% CI 1.8–6.5)	DLT: reversible transaminase elevation occurs at ≥100 mg;Overall safety is good	NCT02481154	[757]
	Vorasidenib/Ivosidenib	LGG	49	Ⅰ	Vorasidenib 50 mg: reduced 2-HG by 92.6% (95% CI 76.1–97.6); Ivosidenib 500 mg: reduced 2-HG by 91.1% (95% CI 72.0–97.0)	NA	NCT03343197	[758]
	Terameprocol (specific protein 1 transcription inhibitor)	rHGG	20	Ⅰ	AUC < 5 μg×h/ml for all dose groups	Well tolerated	NCT02575794	[740]
	Olutasidenib	Relapsed/Refractory *IDH1* mutant glioma	26	Ⅰb/Ⅱ	DCR: 48% (objective response+SD); PR: 8% (2 cases); SD (SD ≥4 months): 32% (8 cases)	No DLTs were observed. Grade 3–4 AEs (≥10%): alanine aminotransferase increased (12%); aspartate aminotransferase increased (12%)	NCT03684811	[759]
	2-hydroxyoleic acid (2-OHOA)	rHGGOther advanced solid tumors	54	Ⅰ/ⅡA	The clinical benefit rate of HGG patients was 24% (5/21), including 1 case of significant remission lasting >2.5 years	Major AEs: reversible grade 1–2 nausea, vomiting, and diarrhea	NCT01792310	[741]
	Selinexor (oral XPO1 selective inhibitor)	rGBM Group A (preoperative administration, *n=*8); Group B (50 mg/m^2^, twice a week, *n*=24); Group C (60 mg, twice a week, *n*=14); Group D (80 mg, once a week, *n*=30)	76	Ⅱ	*ORR*: 8.8% overall (1 CR and 2 durable PR in group D)	Common AEs: fatigue (61%), nausea (59%), decreased appetite (43%), and thrombocytopenia (43%); Serious AEs: 34% of patients, 1 fatal event (1.3%)	NCT01986348	[742]
	Infigratinib (BGJ398) (oral FGFR1–3 selective inhibitor)	Recurrent/Progressive gliomas with *FGFR* gene alterations	26	Ⅱ	PFS-6: 16%; mPFS: 1.7 months; *ORR*: 3.8%	Hyperphosphatemia (76.9%, grade 3 3.8%) was the main toxicity	NCT01975701	[760]
	Dabrafenib (BRAF inhibitor)+trametinib (MEK inhibitor)	*BRAFV600E* mutation-positive recurrent/Progressive glioma	58	Ⅱ	HGG cohort: *ORR* 33% (3 CR+12 PR), median follow-up 12.7 months; LGG cohort: *ORR* 69% (1 CR+6 PR+2 MR), median follow-up 32.2 months	Incidence of grade ≥3 AEs: 53%; Common grade ≥3 AEs: fatigue (9%) and neutropenia (9%)	NCT02034110	[743]
Targeted therapy+RT	Olaparib (PARP inhibitor)+RT (40 Gy/15 fractions)	Patients with GBM who are not suitable for radical chemoradiotherapy	16	Ⅰ	mOS: 10.8 months (80% CI 7.3–11.4); mPFS: 5.5 months (80% CI 3.9–5.9)	The MTD was not reached. Cognitive function: no significant effect	PARADIGM	[744]
Targeted therapy+chemoradiotherapy	Olaparib (PARP inhibitor) +standard chemoradiotherapy (Stupp regimen)	Unresectable ndGBM	30	Ⅰ	mPFS: 6.2 months;mOS: 19.8 months; 2-year survival rate: 36.7% (better than historical data)	MTD: olaparib 100 mg bid (3 d a week) (applicable to both concurrent chemoradiotherapy and maintenance phases); DLT: 5 cases (4 during radiotherapy and 1 during maintenance, mainly hematological toxicity)	OLA-TMZ-RTE-01	[745]
	p38-MAPK inhibitor ralimetinib+chemoradiotherapy	ndGBM	18	Ⅰ	MTD: 100 mg/12 h	AEs of grade ≥ 3: hepatocellular carcinoma (13%), dermatitis/rash (7%), and lymphocytopenia (7%)	NCT02364206	[746]
	Experimental group: veliparib (PARP inhibitor) +concurrent chemoradiotherapy → adjuvant veliparib+TMZ Control group: standard TMZ chemoradiotherapy	MGMT-unmethylated ndGBM	125	Ⅱ	Experimental group vs. control group: PFS-6: 46% vs. 31% (not statistically significant difference); mOS: 12.7 months vs. 12.8 months	Grade 3–4 AEs: thrombocytopenia/neutropenia	VERTU	[761]
Targeted therapy+TMZ	TRC102+TMZ	rGBM	19	Ⅱ	*ORR*: 0%; mOS: 11.1 months (95% CI 8.2–17.9); mPFS: 1.9 months (95% CI 1.8–3.7); PFS-6: 10.5% (95% CI 1.3–33.1);Long-term survivors: 2 patients with PFS ≥ 17 months and OS > 32 months	Grade 3 AEs: lymphocytopenia (*n*=2);Grade 4 AEs: thrombocytopenia (*n*=1);Overall safety: acceptable, no treatment-related deaths	NCT02395692	[762]
	Veliparib+TMZ	ndGBM with MGMT promoter methylation	447	Ⅱ/Ⅲ	Placebo group: mOS 24.8 months (90% CI 22.6–27.7); Veliparib group: mOS 28.1 months (90% CI 24.3–33.3); Statistical difference: the pre-specified endpoint was not reached	Major toxicity (grade 3–4): increased incidence of hematological toxicity (neutropenia, thrombocytopenia, etc.), but generally well tolerated	NCT02152982	[763]
	SurVaxM (peptide vaccine targeting survivin)+adjuvant therapy: TMZ	ndGBM	64	Ⅱa	PFS-6: 95.2% (60/63); mPFS: 11.4 months; mOS: 25.9 months (calculated from the first dose)	Well tolerated, no vaccine-related serious AEs	NCT02455557	[747]
Targeted therapy+anti-angiogenesis therapy	Experimental group: TVB-2640+bevacizumab; Control group: bevacizumab only in the first cycle	Recurrent high-grade astrocytoma	25	Ⅱ	*ORR*: 56% (CR 17%+PR 39%); PFS-6: 31.4% (vs. historical control 16%, *P*=0.008); OS-6: 68%	Major grade 1–2 AEs: palmar-plantar erythra, dysesthesia, and hypertension	NA	[764]
Immunotherapy	Neoadjuvant Pembrolizumab	rGBM	25	Ⅱ	PFS-6: 19.5% (95% CI 9.29–41.2); neoadjuvant therapy was not proven to significantly improve survival	NA	NCT02852655	[752]
	Pembrolizumab	PD-L1+rGBM	26	Ⅰb	*ORR*: 8% (2 PRs, lasting 8.3–22.8 months); PFS-6 rate: 37.7% (mPFS 2.8 months); 12-month OS rate: 58% (mOS 13.1 months)	73% of patients experienced AEs; grade 3–4 TRAEs occurred in 19% of patients, and no grade 5 events	NCT02054806	[765]
Dual-immunotherapy	Single-drug group: Ipilimumab/Nivolumab; Combination group: Ipilimumab+Nivolumab	Unifocal supratentorial ndGBM	32	Ⅰ	Combination therapy group (*n*=14): mPFS: 16.1 months; mOS: 20.7 months	The overall tolerability was good: 1 DLT in each monotherapy group and no DLT in the combination group	NCT02311920	[766]
	Local injection into the brain: ipilimumab +nivolumab	rGBM:Cohort 1 (IPI, *n*=3);Cohort 2 (IPI+NIVO, *n*=24)	27	Ⅰ	mOS: 38 weeks (6-month OS rate 74.1%, 1-year OS rate 40.7%, 2-year OS rate 27%), significantly better than historical controls (*P*=0.003); mPFS: 11.7 weeks	2 cases of reversible postoperative neurological deterioration (responsive to hormonal therapy); no other CNS toxicity	NCT03233152	[753]
	Nivolumab±ipilimumab	rHGG Cohort 4: only NIVO dose escalation (*n*=16); Cohort 7: NIVO+IPI dose escalation (*n*=28)	44	Ⅰ	mOS: cohort 4: 42 weeks (95% CI 26–57); cohort 7: 35 weeks (95% CI 29–40)	Most common AEs: fatigue; no grade 5 AEs	NCT03233152	[754]
Immunotherapy+RT	Durvalumab+hypofractionated SRS	rGBM	6	Ⅰ	Median progression-free interval: 2.3 mouths; mOS: 16.7 mouths	DLT: 1 case of grade 3 vestibular neuritis (immune-related); overall well tolerated	NCT02866747	[767]
	RT+CXCL12 neutralizing aptamer (olaptesed pegol, NOX-A12)	MGMT is not methylated in patients with incompletely resected ndGBM	10	Ⅰ/Ⅱ	mPFS: 174 d; PFS-6: 40.0%; mOS: 389 d	Safety: no DLT or treatment-related death; MTD: not reached, RP2D 600 mg/week	NCT04121455	[768]
	Pembrolizumab (PD-1 inhibitor)+re-irradiation	Cohort A: bevacizumab-naïve (*n*=30); Cohort B: bevacizumab-experienced patients with rGBM (*n*=30)	60	Ⅱ	Cohort B: 6-month *OS* 57% (reached the primary endpoint); Cohort A: no significant improvement in survival; *ORR*: Cohort A 3.3%, Cohort B 6.7%	Most toxicity levels ≤ 3	NCT03661723	[769]
Immunotherapy+gene therapy	VDX-controlled IL-12 gene therapy+nivolumab	rGBM	21	Ⅰ	mOS: 9.8 months	Toxicity is manageable (similar to single-agent), dose-dependent, and reversible	NCT03636477	[770]
Immunotherapy+standard treatment	IGV-001 personalized Immunotherapy+standard treatment	ndGBM	33	Ⅰ/Ⅱ	mPFS: 9.8 months (vs. 6.5 months in historical controls, *P*=0.0003)	6 patients (18%) experienced ≤ grade 3 AEs	NCT02507583	[771]
Immunotherapy+RT+anti-angiogenesis therapy	Pembrolizumab+HFSRT and bevacizumab	rGBM/anaplastic astrocytoma: bevacizumab-naïve group (*n*=24); bevacizumab-resistant group (*n*=8)	32	Ⅰ/Ⅱ	Bevacizumab-naive group: *ORR* 83% (CR+PR); mOS 13.45 months; PFS 7.92 months; Bevacizumab-resistant group: *ORR*: 62%; mOS: 9.3 months; PFS: 6.54 months	Common AEs: proteinuria (40.6%), fatigue (25%), ALT elevation (25%), and hypertension (25%)	NCT02313272	[772]
	Cohort A: pembrolizumab+bevacizumab (*n*=50); Cohort B: pembrolizumab monotherapy (*n*=30)	Bevacizumab-naïve patients with rGBM	80	Ⅱ	Cohort A: PFS-6 26.0% (95% CI 16.3–41.5); mOS 8.8 months (95% CI 7.7–14.2); *ORR* 20%; Cohort B: PFS-6 6.7% (95% CI 1.7–25.4); mOS 10.3 months (95*%* CI 8.5–12.5); *ORR* 0%	Well tolerated	NA	[773]
RT	HSRT: 25 Gy/5 fractions vs. 35 Gy/5 fractions	rGBM	40	ⅡA	25 Gy group vs. 35 Gy group: mPFS: 4.9 months vs. 5.2 months (*P*=0.23); PFS-6 rate: 40% in both groups; mOS: 9.2 months vs. 10 months (*P*=0.201); Patients with treatment-related necrosis had a longer mOS (14.1 vs. 8.7 months, *P*=0.003)	35 Gy group vs. 25 Gy group: the incidence of grade ≥3 radiation necrosis: 16% (3 cases) vs. 5% (1 case) (*P*=0.267)	NCT01464177	[748]
	Re-RT	rHGG	90	Ⅱ	mOS: 17 months (95% CI 14–19)	Grade 2-3 radiation necrosis: 10% (9 cases); Stable to progressive neurocognitive function	NCT02567539	[749]
	PT vs. intensity-modulated radiation therapy	ndGBM	67	Ⅱ	Cognitive function: no significant difference (*HR*=0.88, *P*=0.74); No difference in PFS (*HR*=0.74) and OS (*HR*=0.86)	The PT group had less grade ≥2 toxicity (0.35/person vs. 1.15/person, *P*=0.02); the incidence of fatigue was lower in the PT group (24% vs. 58%, *P*=0.05)	NCT01854554	[750]
	Innovative fractionation scheme: Phase 1: 3.96 Gy×7 fractions (1 fraction per day); Phase 2: 1.0 Gy×9 fractions (3 fractions per day)	rGBM	14	Ⅰ	All patients completed the full course of treatment	No DLT reported	NCT03557372	[751]
	Chemoradiotherapy; RT alone	IDH wild-type/*TERT* mutant LGG	-	Ⅱ	Chemoradiotherapy group vs. RT group mOS: 25 months vs.17 months; *HR*=0.271, *P*=0.017; 1-year *OS* rate: 94.1% vs. 74.6%; PFS: 16 months vs. 7 months (*P=*0.840)	NA	NCT02766270	[774]
RT+chemotherapy	Hypofractionated proton RT (35–40 Gy/5–10 fractions)+TMZ (concurrent+adjuvant)	Elderly patients (≥ 65 years old) with ndGBM	39	Ⅱ	12-month OS rate: 56% (95% CI 39–72); mOS: 13.1 months (95% CI 11.1–19.1); mOS in historical controls: 6–9 months	Grade 3 AEs: CNS necrosis (10%) and thrombocytopenia (3%); No grade 4 AEs or treatment-related deaths	NCT03778294	[775]
	Chloroquine+chemoradiotherapy	ndGBM	13	Ⅰ	MTD: 200 mg/d; mOS: 16 months	QTc prolongation; Irreversible blurred vision; Nausea/Vomiting	NCT02378532	[776]
	Hypofractionated IMRT+TMZ+GM-CSF	ndGBM	41	Ⅱ	PFS-6: 68.3% (95% CI 54.0–82.6); mOS: 16.7 months (95% CI 10.5–22.9)	No grade 3–4 hematological toxicity; Non-hematologic grade 3 toxicity due to GM-CSF	NCT02663440	[777]
RT+anti-angiogenesis therapy	Re-radiotherapy (35 Gy/10 fractions) Bevacizumab (10 mg/kg, once every two weeks)	rGBM	170	Ⅱ	OS: no difference (10.1 months vs. 9.7 months, *HR*=0.98, *P*=0.46); PFS: significant improvement (7.1 months vs. 3.8 months, *HR*=0.73, *P*=0.05); PFS-6: 54.3% vs. 29.1% (*P*=0.001)	≥Grade 3 acute treatment-related AEs: 5%; No late high-grade AEs	NCT01730950	[778]
Chemotherapy	Continuous infusion of topotecan using the CED system	rGBM	5	Ⅰb	All patients had a significant reduction in tumor proliferating cells	Safety: only 1 patient (20%) had a grade 3 AE (intraoperative supplementary motor area syndrome), and no grade 4 AEs or treatment-related deaths	NCT03154996	[779]
	SM-1+TMZ	rHGG	13	Ⅰ	CR in 1 case, PR in 2 cases	DLT was observed. The MTD was not reached	CTR20221641	[780]
Standard Stupp regimen+anti-angiogenesis therapy	Anlotinib (multikinase antiangiogenic inhibitor)+standard Stupp regimen	ndGBM	33	Ⅱ	mPFS: 10.9 months (95% CI 9.9-18.7); 12-month PFS rate: 48.5%; mOS: 17.4 months (95% CI 14.5-21.1); 12-month OS rate: 81.8%	During concurrent chemoradiotherapy: hypertriglyceridemia (58%), hypoalbuminemia (46%), and hypercholesterolemia (46%)	NCT04119674	[781]
Others	BXQ-350 (nanoencapsulated saposin C preparation, sphingolipid metabolism regulator)	Advanced/Recurrent solid tumors or HGGs	86	Ⅰ	8 patients achieved PFS ≥ 6 months (2 PR+6 SD, 3 of which had shrinkage of target lesions); Long-term benefit: 2 patients remained progression-free for >7 years	No DLT was observed, and MTD was not reached (2.4 mg/kg was the highest dose tested)	NCT05178342	[782]
	Metronomic antiangiogenic chemotherapy combination regimen	Relapsed/refractory medulloblastoma	40	Ⅱ	6-month DCR: 57.5% (23/40); *ORR*: 45.0% (18/40); mOS: 25.5 months (range 10.9–40.0); mPFS: 8.5 months (range 1.7–15.4)	Common grade 3–4 AEs: myelosuppression, infection, seizure, and headache	NCT01356290	[783]
	Standard treatment: RT+TMZ; Experimental group: DSF (250–375 mg/d)+Cu; Maintenance period: TMZ+DSF (500 mg/d)+Cu	ndGBM	33	Ⅰ/Ⅱ	Overall population: no significant improvement in OS or PFS (compared to historical controls); *BRAF* mutation subgroup: 3 patients experienced long-term remission (significant benefit signal)	MTD: DSF 375 mg/d	NCT02715609	[786]
	Low-intensity pulsed ultrasound+intravenous microbubbles+nab-paclitaxel	rGBM	17	Ⅰ	Drug concentration increased: Non-ultrasound area vs. ultrasound area Paclitaxel: 0.037 μmol/L vs. 0.139 μmol/L (3.7 times, *P*<0.0001); Carboplatin: 0.991 μmol/L vs. 5.878 μmol/L (5.9 times, *P=*0.0001)	DLT: 1 case of grade 3 encephalopathy in the 260 mg/m^2^ group (8%); Common grade 3–4 AEs: neutropenia (47%), leukopenia (29%), and hypertension (29%)	NCT04528680	[784]
	SNA nanoconjugate NU-0129	rGBM	8	0	SNA was confirmed to cross the blood-brain/blood-tumor barrier	No grade 4/5 treatment-related toxicity	NCT03020017	[785]
ADC	Sacituzumab govitecan	Breast cancer BrM and rGBM	25	Prospective window trial	Breast cancer BrM vs. rGBM PFS: 8 months vs. 2 months; OS: 35.2 months vs. 9.5 months; *ORR*: 38% vs. 29%	Grade ≥3 AEs: neutropenia (28%) and hypokalemia (8%)	NCT03995706	[633]

HCG. High-grade glioma; rHGG. Recurrent HCG; RP2D. Recommended phase 2 dose; IDH. Isocitrate dehydrogenase; DLT. Dose limiting toxicity; PFS. Progression-free survival; mPFS. Median progression-free survival; *ORR*. Objective response rate; MTD. Maximum tolerated dose; AEs. Adverse events; rGBM. Recurrent glioblastoma; BET. Bromodomain and extraterminal domain; LGG. Low-grade glioma; 2-HG. 2-hydroxyglutarate; CI. Confidence interval; NA. Not applicable; AUC. Area under curve; DCR. Disease control rate; PR. Partial response; SD. Stable disease; 2-OHOA. 2-hydroxyoleic acid; XPO1. Exportin 1; CR. Complete response; FGFR. Fibroblast growth factor receptor; BRAF. B-Raf proto-oncogene, serine/threonine kinase; MEK. Mitogen-activated protein kinase; PARP. Poly ADP-ribose polymerase; RT. Radiation therapy; MR. Minor responses; MAPK. Mitogen‑activated protein kinase; TMZ. Temozolomide; MGMT. O^6^-methylguanine-DNA methyltransferase; PD-L1. Programmed cell death-ligand 1; IPI. Ipilimumab; NIVO. Nivolumab; CNS. Central nervous system; SRS. Stereotactic radiosurgery; CXCL12. C-X-C motif chemokine ligand 12; PD-1. Programmed death 1; VDX. Veledimex; HFSRT. Hypofractionated stereotactic radiotherapy; re-RT. Re-irradiation; *HR*. Hazard ratio; OS. Overall survival; PT. Proton radiation therapy; TERT. Telomerase reverse transcriptase; CRT. Chemoradiotherapy; IMRT. Intensity modulated radiation therapy; GM-CSF. Granulocyte-macrophage colony-stimulating factor; CED. Convection-enhanced drug delivery; SNA. Spherical nucleic acid; ADC. Antibody-drug conjugates; DSF. Disulfiram; Cu. Copper; hrBMP4. Human recombinant bone morphogenetic protein 4; PFS-6. 6-month progression-free survival; ndGBM. Newly diagnosed GBM; NOX-A12. Olaptesed pegol (NOX-A12); ALT. Alanine aminotransferase; HSRT. Hypofractionated stereotactic radiotherapy; QTc. Corrected QT interval; Stupp. Radiotherapy with concomitant temozolomide followed by adjuvant temozolomide; OS-6. 6-month OS overall survival

## Future directions

### Multidisciplinary intersection and cooperation

Research on the BTME requires interdisciplinary collaboration [787], [788], [789]. Close cooperation among biologists, clinicians, engineers, and data scientists is essential to accelerate advancements. Advances in transcriptomics, metabolomics, microbiomics, and related technologies enable multidimensional interrogation [29], [512], [790], [791]. Improved mechanistic understanding of the BTME supports the development of potential treatment strategies and enables clinicians to better evaluate treatment efficacy [792].

### .1 5.2AI and big data analysis

Big data, cloud computing, and AI have broad applications in the study of the BTME [793], [794]. Integration of multiomics data and clinical information enables the construction of large-scale TME databases [124], [420], providing crucial resources for identifying new biomarkers and therapeutic targets. These advances also provide a foundation for data-driven models that support mechanistic investigation and translational research in the BTME.

Building on this foundation, AI-based approaches are increasingly being applied to the diagnosis, monitoring, and therapeutic evaluation of brain tumors. Machine learning approaches can predict patients’ response to treatments, supporting personalized treatment strategies [795], [796], [797]. An AI-driven image analysis facilitates the identification of key TME features [798]. Longitudinal imaging studies of large BrM cohorts revealed distinct growth dynamics between untreated and recurrent lesions [799], while deep and handcrafted feature extraction enables tumor classification and prognostic assessment [800]. Moreover, deep learning algorithms can be used to analyze cell distribution and morphology in pathological slides and identify features associated with prognosis [801], [802], [803], [804], [805]. Automated segmentation and classification from reconstructed microwave brain images [806], together with non-invasive modeling of brain iron homeostasis [801], [802], further highlight the expanding role of AI in diagnosis, monitoring, and treatment evaluation.

### Clinical trials and translational studies

Clinical trials remain essential for validating the safety and efficacy of new therapeutic strategies [561], [571]. The design and implementation of clinical trials should fully account for the dynamic complexity of the BTME. Integration of multiomics analyses within clinical trials can help assess treatment-induced TME changes and optimize therapeutic strategies [790], [791]. Translational research serves as a critical bridge between basic discoveries and clinical application, enabling preclinical validation of novel therapies and informing trial design [230], [620], [664]. Systems-level approaches, including gene regulatory network construction and dynamic modelling, facilitate the identification of key regulators and the prediction of treatment responses and resistance mechanisms. For example, TLS density has prognostic value in breast cancer BrM [308]. Despite extensive mechanistic research, clinical translation in brain tumors remains relatively limited. To clarify the current translational landscape, recent clinical trials in brain tumors are summarized in Table 1, Table 2, Table 3, Table 4, Table 5, Table 6.

## Conclusions

The BTME is a highly interconnected and plastic ecosystem that orchestrates tumor initiation, progression, therapeutic resistance, and clinical outcome [807]. Advances in multiomics, spatial technologies, and systems-level analyses have refined our understanding of its cellular heterogeneity and spatiotemporal structure [44], [150], [808]. Spatial multiomics enables mapping of cell-type distribution and spatial interaction networks [809]. Previous studies have collectively revealed the central roles of tumor-immune-neuronal communication [131], [810], [811], [812], immunosuppressive myeloid networks [30], [34], stromal and vascular remodeling [312], [813], [814], [815], [816], [817], and metabolic adaptation sustain tumor growth, immune escape, and microenvironmental remodeling [807], [818], [819]. Beyond tumor cells themselves [820], [821], neuronal signaling [811], [812], dysfunctional immunity [156], [665], [822], neutrophils [823], TLSs, microbiome-associated components [312], [813], [814], [815], [816], vasculature, and ECM dynamics [817] collectively contribute to BTME complexity.

Future therapeutic progress will depend on strategies that target the BTME as an integrated system rather than targeting isolated components. Reprogramming immunosuppressive myeloid populations (particularly TAMs and MDSCs) [34], [824], combining vascular normalization with immune activation through VEGF/VEGFR-directed approaches [817], and advancing T cell-based immunotherapies (such as CAR-T cells and tumor vaccines) may be promising for brain tumors [256], [257], [258], [259], [825]. Medium- to long-term opportunities lie in targeting signaling hubs shared across BTME compartments, such as STAT3, TGF-β, and CXCL12-CXCR4 axis, to achieve broader microenvironmental reprogramming. The clinical maturation of VEGF and PD-1/PD-L1 co-targeting in lung cancer [561], [562], [563], together with early-stage CNS-directed STAT3 inhibition (NCT01904123; NCT05689619), illustrates both the feasibility and the developmental gaps across BTME pathways.

Future progress in BTME-directed therapy will likely rely on the convergence of molecular profiling, dynamic monitoring, and targeted delivery strategies. Such integration may enable more precise patient stratification, more rational therapeutic combinations, and more effective clinical implementation in brain tumors. Accordingly, future BTME-targeted therapies will require aligning cell type-defined vulnerabilities, pathway-centric interventions, and rational combination strategies. Over the next decade, the most impactful advances are likely to arise from reprogramming immunosuppressive myeloid-stromal networks, combining vascular normalization with immune activation, and synergizing advanced T cell therapies with BTME modulation. As multiomics, AI-assisted modelling and innovative delivery platforms continue to mature, BTME-informed precision immunotherapy holds strong potential to transform brain tumor treatment and offer renewed hope for patients.

## Abbreviations

3DThree-dimensional

ACAT1Acetyl-CoA acetyltransferase 1

αCD40Anti-CD40 antibody

ADCsAntibody-drug conjugates

AktProtein kinase B

AIArtificial intelligence

AMPARsAlpha-amino-3-hydroxy-5-methyl-4-isoxazole propionic acid receptors

APCsAntigen-presenting cells

B2Mβ2-microglobulin

BBBBlood-brain barrier

BDNFBrain-derived neurotrophic factor

BrMsBrain metastases

BTMEBrain tumor microenvironment

C3Component 3

CAFsCancer-associated fibroblasts

CD Cluster of differentiation

cDC1Conventional dendritic cell type 1

cGAS-STINGCyclic GMP-AMP synthase-stimulator of interferon genes

CNSCentral nervous system

CSCsCancer stem cells

ctDNA Circulating tumor DNA

CTLs Cytotoxic T lymphocytes

DCsDendritic cells

DLG3Discs large homolog 3

ECMExtracellular matrix

E-MDSCsEarly progenitor MDSCs

EVsExtracellular vesicles

FOXP3Forkhead box P3

GABAGama-aminobutyric acid

GBMGlioblastoma

GPNMBGlycoprotein nonmetastatic melanoma protein B

GPX4Glutathione peroxidase 4

GSCGlioma stem cells

HEVs High endothelial venules

HEXBHexosaminidase B

HIF-1αHypoxia-inducible factor-1α

HSP47Heat shock protein 47

ICAM-1Intercellular cell adhesion molecule-1

ICIsImmune checkpoint inhibitors

IFITM1Interferon-induced transmembrane protein 1

IFN-γInterferon-γ

IGFInsulin like growth factor

ILInterleukin

IRF8Interferon regulatory factor-8

JAK/STATJanus kinase/signal transducer and activator of transcription

KDM6ALysine demethylase 6A

LAG3Lymphocyte activation gene 3

LGALS3Immunosuppressive galactose-specific lectin 3

LILRB2Leukocyte immunoglobulin like receptor B2

LLMsLipid-laden macrophages

MDMsMonocyte-derived macrophages

MDSCsMyeloid-derived suppressor cells

MHCMajor histocompatibility complex

MMPMatrix metalloproteinase

mTORMammalian target of rapamycin

ndGBMNewly diagnosed GBM

NgR1Nogo-66 receptor 1

NKNatural killer

NKCC1Sodium potassium chloride cotransporter 1

NRF2Nuclear factor erythroid 2-related factor 2

NSCsNeural stem cells

NSCLCNon-small cell lung cancer

oHSVOncolytic herpes simplex virus

OSOverall survival

OSMOncostatin M

PD-1Programmed death 1

PD-L1Programmed death ligand-1

PFSProgression-free survival

RBM15RNA binding motif protein 15

rGBMRecurrent glioblastoma multiforme

SCFAsShort-chain fatty acids

SCLCSmall cell lung cancer

SOX2SRY-box transcription factor 2

STAT3Signal transducer and activator of transcription 3

TCRT cell receptor

TGF-βTransforming growth factor-β

TAMsTumor-associated macrophages

TfRTransferrin receptor

TILsTumor-infiltrating lymphocytes

TIM-3T cell immunoglobulin and mucin domain-containing protein3

TLRToll-like receptor

TLSsTertiary lymphoid structures

TMETumor microenvironment

TMZTemozolomide

TNFTumor necrosis factor

TregsRegulatory T cells

TREM2Triggering receptor expressed on myeloid cells 2

VEGFVascular endothelial growth factor

VMVascular mimicry

## Ethics approval and consent to participate

Not applicable.

## Funding

This work was supported by the Advanced Lung Cancer Targeted Therapy Research Foundation of China (CTONG-YC20210303), the Chen Xiao-Ping Foundation for the Development of Science and Technology of Hubei Province (CXPJJH121005-01), the Health Research Project of Hunan Provincial Health Commission (W20242005), the National Clinical Research Center for Geriatric Disorders, Xiangya Hospital, Central South University (2022LNJJ10), the Hunan SJA Laboratory Animal Co., Ltd (20241205CY), and the National Multidisciplinary Cooperative Diagnosis and Treatment Capacity (lung cancer z027002).

## Data Availability

Not applicable.
